# Aryl diazonium salts and benzene derivatives bearing two electron-donor substituents: chemical and mechanistic behaviours†

**DOI:** 10.1039/d4ra00652f

**Published:** 2024-03-14

**Authors:** Gabriele Micheletti, Carla Boga

**Affiliations:** a Department of Industrial Chemistry, Alma Mater Studiorum – Università di Bologna Via Gobetti 85 40129 Bologna Italy gabriele.micheletti3@unibo.it

## Abstract

The reaction between benzene derivatives 1–4 and *p*-substituted benzenediazonium tetrafluoroborates 5a–c provided novel azo-coupling products in high yields under mild conditions. The monitoring of the reaction progress using ^1^H-NMR provided mechanistic information on both the relative reactivity of the reagents and the possibility to detect novel reaction intermediates.

## Abstract 2

Study of the electrophilic aromatic substitution reaction between benzenediazonium salts and neutral carbon nucleophiles.

## Graphical abstract

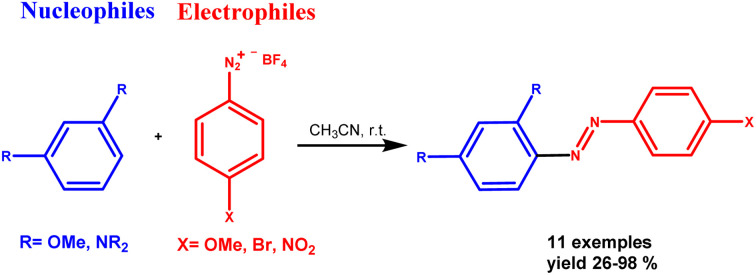

## Introduction

Electrophilic aromatic substitution (S_E_Ar) is one of the most widely studied types of reactions in organic chemistry. Numerous studies on this reaction have been carried out to obtain both new compounds and mechanistic information. In particular, a class of compounds used for mechanistic studies is that of 1,3,5-tris(*N*,*N*-dialkylamino)-benzenes belonging to neutral aromatic substrates rich in electrons due to the presence of three electron-donating groups on the ring. These compounds are able to react as carbon nucleophiles with a plethora of electrophiles such as, halogens,^1,2^ protons, and^3–12^ alkyl-,^13^ acyl-,^14–16^ and aryl-halides.^17–19^

Moreover, the reaction of 1,3,5-tris(*N*,*N*-dialkylamino)-benzenes with charged^20^ and neutral carbon electrophiles has recently been studied, providing interesting information on the relevant reaction intermediates.^21–23^

From long time our interest lies in the reaction between 1,3,5-tris(*N*,*N*-dialkylamino)-benzene derivatives and aryl diazonium salts from which it was possible to obtain evidence for the reversibility of the azo-coupling reaction and to detect the related Wheland-like reaction intermediates. Moreover, the reaction gave access to novel products of interest in applied chemistry.^24–26^ For example, the reaction between 1,3,5-tris(*N*,*N*-dialkylamino)-benzenes and 2 equivalents of *p*-substituted benzenediazonium salts provided dicationic species, which collapsed to new benzimidazole derivatives with the expulsion of *p*-substituted anilines.^27^

Unlike 1,3,5-tris(*N*,*N*-dialkylamino)-benzene derivatives, just a few examples are reported for the electrophilic aromatic substitution involving 1,3-*N*,*N*-dialkylamino benzenes.^28–31^

Based on these, we decided to study the azo-coupling reaction between these nucleophiles and *para*-substituted benzenediazonium salts to possibly obtain new compounds of interest for application in fields such as optoelectronics^32,33^ and dyes^34^ together with further mechanistic information on this kind of reaction.

## Results and discussion

**Scheme 1:**
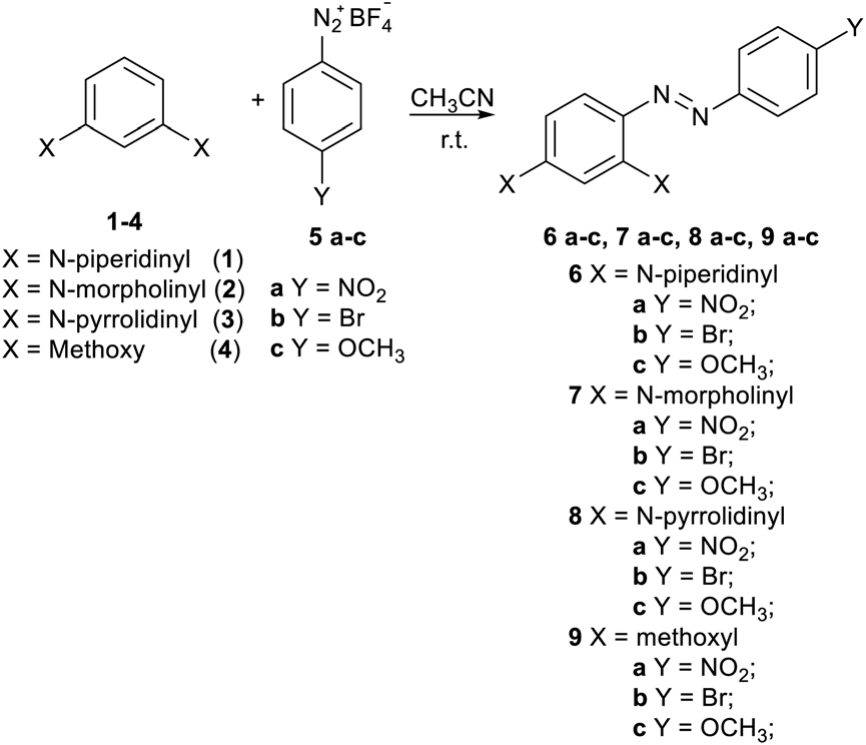
Reaction between di-substituted benzenes 1–4 and *p*-substituted benzenediazonium salts 5a–c.

A new series of azo-coupling compounds 6a–c, 7a–c, 8a–c, and 9a–c were synthesized *via* the reaction of 1,3-di-substituted benzenes 1–4, chosen as nucleophiles, and benzenediazonium salts 2a–c, chosen as electrophiles (Scheme 1).

The reactions were conducted at room temperature in a 1 : 1 molar ratio of reagents, and the final products were purified by chromatography on silica gel and fully characterized.

**Scheme 2:**
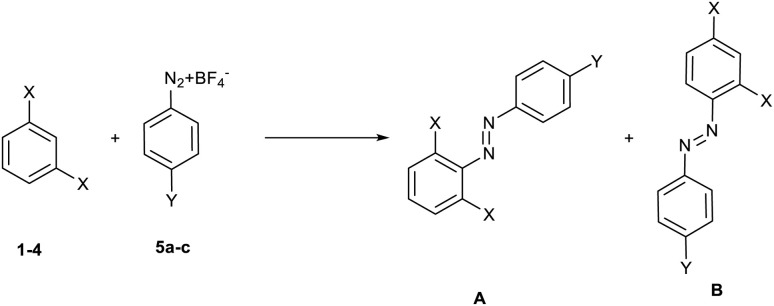
Possible regioisomers from the reaction between 1,3-disubstituted benzenes 1–4 and diazonium salts 5a–c.

The reaction can lead to the formation of two distinct isomeric products (A and B, Scheme 2), resulting from the attack of the diazonium salt in *ortho–ortho* (A) or in *ortho*–*para* (B) position with respect to the two substituents present on the nucleophilic reagent.

From the study of the ^1^H-NMR spectra of the purified final compounds, it has been possible to note (see ^1^H-NMR spectra in ESI†) that the nucleophilic moiety of the products shows three different signals. This behaviour is possible only for the product resulting from the attack of diazonium salt in the *ortho*–*para* position (case B), as it was expected on the basis of steric hindrance considerations.

From the data reported in Table 1, it is possible to note that the syntheses with 1,3-dimethoxybenzene (4) (entry 10–12) have required a very longer reaction time than that of all other reactions. Moreover, in the reaction with a *para*-methoxy benzene diazonium salt (5c), any product was obtained after a longer reaction time (72 h). These behaviours are due to the lower activation of the benzene ring towards a nucleophilic attack caused by the two methoxy substituents, compared with the amino groups and it is also similar to those reported previously.^35–37^

**Table 1:** Reactions between 1–4 and 5a–c

Entry	Nucleophile	Electrophile	Reaction time	Product	Yield^a^
1	1	5a	20 min	6a	98%
2	1	5b	20 min	6b	97%
3	1	5c	20 min	6c	78%
4	2	5a	30 min	7a	94%
5	2	5b	30 min	7b	80%
6	2	5c	30 min	7c	75%
7	3	5a	10 min	8a	95%
8	3	5b	10 min	8b	77%
9	3	5c	10 min	8c	73%
10	4	5a	24 h	9a	77%
11	4	5b	48 h	9b	26%
12	4	5c	72 h	9c	0%

a Yield after purification by chromatography on silica gel.

The reactions with di(pyrrolidinyl)benzene 3 (entries 7–9) show a lower reaction time, due to the strong activation of the benzene ring caused by the two pyrrolidinyl substituents, which makes reagent 3 the stronger nucleophile.

Moreover, it is possible to note, particularly comparing the results reported in entries 10–12 of Table 1, that the electrophilicity of the diazonium salts also influences the progress of the reaction. In fact, as reported in Mayr's electrophilicity scale,^38^ the values for 5a–c are −5.1, −6.6 and −8.4, respectively.

It is important to note that all reactions were conducted in an equimolar amount between the two reagents, and given the formation of tetrafluoroboric acid, we should have obtained a maximum of 50% yield in the reactions, since the released acid would have to react with the nucleophilic reagent deactivating it. On the contrary, except to case 11 and 12 in Table 1, the yields obtained were greater than 50%. A possible explanation for this finding is that the final product is more basic than the starting diaminobenzene reagent. If this were the case, only the protonated product would be found in the reaction crude and the protonated diaminobenzene reagent would be absent.

Given that no data are reported in the literature for the protonation of dialkylaminobenzenes, we decided to carry out the reaction between 1,3-di(pyrrolidinyl) benzene (3) and tetrafluoroboric acid directly in the NMR spectroscopy tube.

It is reported in the literature^3,12,15^ that the protonation of 1,3,5-tris(*N*,*N*-dialkylamino) benzenes manifests the formation of both Wheland intermediates from the C-attack and ammonium salts from the proton attack on the nitrogen atom. Only in the case of protonation on 1,3,5-tripyrrolidinyl benzene, the attack on the carbon atom was detected.

**Scheme 3:**
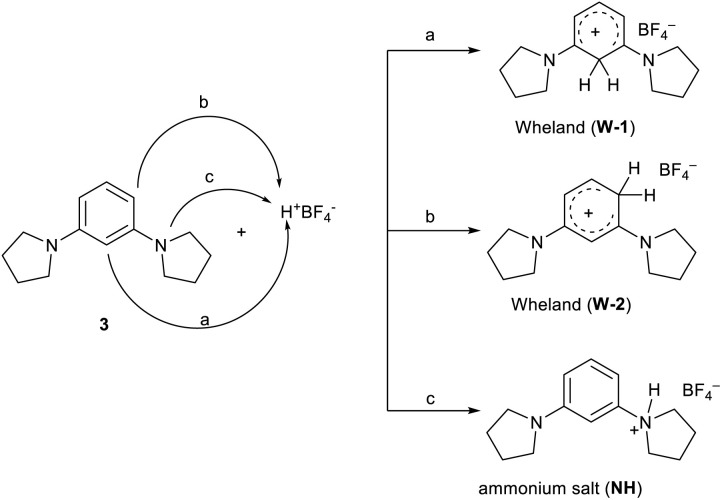
Possible attacks in the protonation reaction of compound 3.

In the present case involving dialkylamino benzene derivatives, there are three possibilities for the attack of the proton (Scheme 3): (i) in the *ortho–ortho* position (W-1), (ii) in the *ortho-para* position (W-2), and (iii) on one of the nitrogen atoms (ammonium salt NH).

**Fig. 1:**
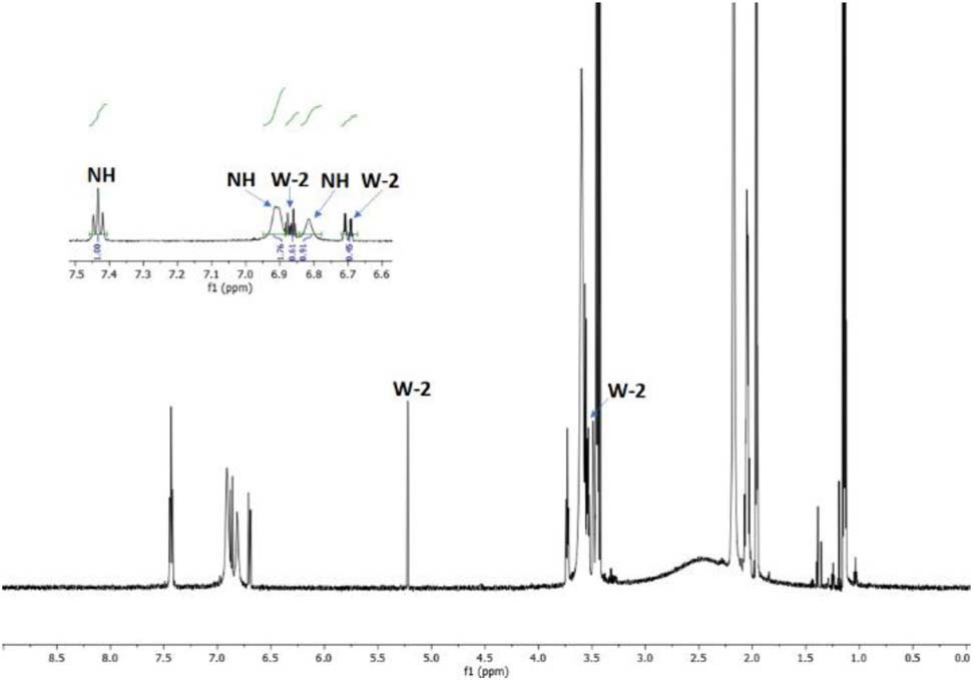
^1^H-NMR spectrum of the product mixture resulting from the protonation reaction of 3, with enlarged view.

The reaction was carried out in CD_3_CN by adding an equimolar amount of a solution of HBF_4_ in Et_2_O to a solution of 3. The mixture was analysed by ^1^H-NMR and 2D-NMR (g-COSY and g-HSQC) experiments. The ^1^H-NMR spectrum (Fig. 1) shows the presence of two sets of signals. In particular, by studying the *g*-COSY spectrum, it is possible to observe that the signals at 7.43, 6.90 and 6.81 ppm are attributed to the same species.

These signals are shifted at a lower field than those of compound 3 (6.98, 5.91 and 5.75, see Fig. S35†); this finding confirms the presence of ammonium salt NH. Theoretically, for the salt NH, the number of signals should be four, but the rapid movement of the proton from one nitrogen atom to another makes the molecule appear symmetrical, in the NMR scale time. This agrees with the relative integration 1 : 2 : 1 of the signals, analogously to what has been previously observed for the protonation of triaminobenzenes.^12^ Furthermore, from the g-COSY experiment, the signals at 6.87, 6.70, 5.21 and 3.50 ppm are correlated with each other. These signals are compatible with the formation of the Wheland-like intermediate. In particular, the signal at 3.50 ppm is very diagnostic, because it shifted in the sp^3^ region of the spectrum. This is also confirmed by the HSQC experiment, which indicates that the signals at 3.50 ppm in the ^1^H-MNR spectrum are related to the signal at about 50 ppm in the ^13^C-NMR spectrum. The presence of four signals for this molecule is consistent with the structure of the W-2 intermediate.

**Fig. 2:**
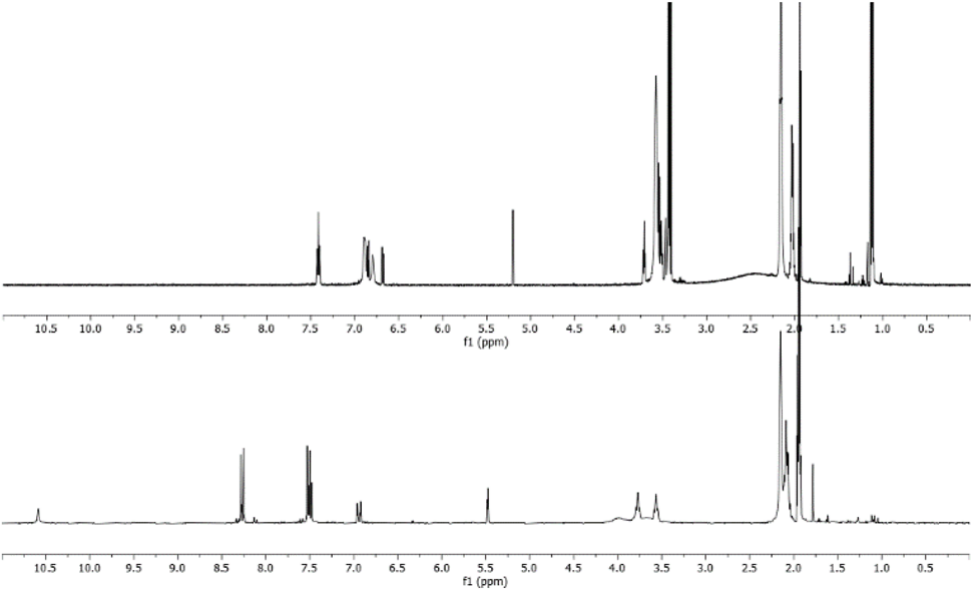
Comparison between the raw spectrum for the reaction between 3 and 5c (lower) and the protonation reaction spectrum of 3 (upper).

This experiment furnished us the possibility to verify if compounds W-2 and/or NH are present in the ^1^H-NMR spectra of the raw reaction mixture between equimolar amounts of 3 and 5c, which implies the absence of the signals belonging to the protonated spices of 3 (Fig. 2).

**Fig. 3:**
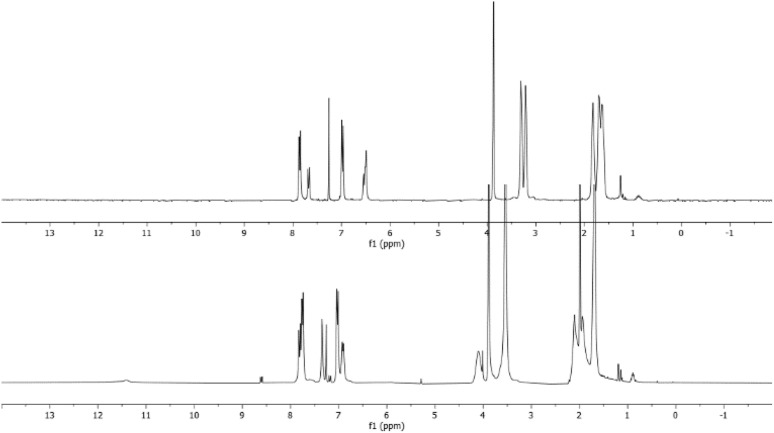
Comparison between the raw reaction spectrum (lower) and the spectrum of purified product (upper) for the reaction between 1 and 5c.

Furthermore, comparison of the spectra of the raw reaction and that of the purified product indicates that the broad signals of the crude reaction mixture shifted to lower fields than those of the purified products (Fig. 3). This implies that the obtained products are in salt form. A similar behaviour was reported in the literature in the reaction between tris-dialkylamino benzene derivatives and electrophiles such as benzofuroxan^19^ and benzofurazan^18^ derivatives.

The only explanation for this is that the neutralization process might have occurred during the chromatographic purification, since this was the only work-up step to which the reactions were subjected.

It was reported in the literature^24–26^ that during the reaction between 1,3,5-tris(*N*,*N*-dialkylamino)-benzene derivatives and benzenediazonium salts, the formation of the relevant Wheland intermediates can be confirmed by NMR analyses at low temperatures. For this reason, we performed some reactions directly in the NMR tube, using deuterated acetonitrile at −35 °C or deuterated dichloromethane at −80 °C, but the ^1^H-NMR spectra did not confirm the presence of the Wheland intermediate, as only the signals belonging to the products in the salt form were present.

These results allow us to affirm that Wheland intermediates, in the azo-coupling reaction, are sufficiently stable, in the NMR time scale, to allow their identification by NMR spectroscopy, only when three strong electron-donor substituents are present on the benzene ring, such as in the case of 1,3,5-tris(*N*,*N*-dialkylamino)-benzenes.

## Conclusion

Novel azo-compounds have been synthesized from benzenediazonium tetrafluoroborates and 1,3-disubstituted benzenes 1–4 in good yields under mild reaction conditions.

The data enabled us to obtain information on the relative reactivity of the studied 1,3-disubstituted benzenes, that is: 1,3-di(pyrrolidinyl)benzene (3) > 1,3-dipiperidinyl benzene (1) > 1,3-di(morpholinyl)benzene (2) > 1,3-di(methoxybenzene) (4).

The study of the progress of the reaction at low temperatures using NMR spectroscopy did not highlight the presence of Wheland intermediates, in contrast to the azo-coupling reaction with 1,3,5-tris(dialkylamino)benzenes. This suggests that the Wheland intermediates are stable enough to be detected by NMR spectroscopy, only when three strong electron-donating substituents are present on the aromatic ring.

## Experimental section

### Material and methods

The reagents used, unless stated otherwise, were purchased from Sigma-Aldrich. Chromatographic purifications (FC) were carried out using glass columns packed with silica gel Geduran Si 60, 0.063–0.200 mm) or neutral alumina at a medium pressure. Thin-layer chromatography (TLC) was performed on silica gel 60 F254-coated aluminium foils. ^1^H-NMR spectra were recorded at 25 °C using Varian spectrometers Inova 300, Mercury 400 or Inova 600 operating at 300, 400 or 600 MHz, respectively. ^13^C-NMR spectra were recorded at 25 °C using Varian spectrometers Mercury 400 or Inova 600 operating at 100.56 or 150.80 MHz, respectively. Chemical shifts were measured in ppm with reference to the solvent CDCl_3_ = 7.26 ppm for ^1^H-NMR and 77.0 ppm for ^13^C-NMR. The *J*-values are given in Hz. Electrospray ionization (ESI)-MS spectra and ESI high-resolution HRMS spectra were recorded using a Waters ZQ 4000 and a Xevo instrument, respectively. For mass analyses, the samples were dissolved in methanol. UV/Vis spectra were recorded using a PerkinElmer Lambda 12 spectrophotometer, at 20 °C in CHCl_3_ and quartz cells (path length of the cell: 1 cm). Melting points (m.p.) were measured using a Büchi 535 apparatus and were uncorrected. ^1^H-NMR, ^13^C-NMR, ESI-MS, and UV/Vis spectra of the synthesized compounds are reported in the ESI.† Syntheses of the dialkylamino benzene derivatives were reported in the literature.^31^ Compounds 5a–c and tetrafluoroboric acid diethyl ether complex are commercially available.

### General procedure

To a solution of 1,3 di-substituted benzene derivative (0.1 mmol, corresponding to 24.4 mg of 1, 24.8 mg of 2, 21.6 mg of 3 and 13.8 mg of 4) in CH_3_CN (5 mL), the selected benzenediazonium salt (0.1 mmol, corresponding to 23.7 mg of 5a, 27.1 mg of 5b and 22.2 mg of 5c) was added at room temperature. The mixture was magnetically stirred, and the reaction progress was monitored through TLC analysis. After solvent removal, the reaction products were purified by column chromatography on silica gel (FC) using appropriate eluents (reported in the characterization).

#### 1,1′-(4-((4-Nitrophenyl)diazenyl)-1,3-phenylene)dipiperidine (6a)

Violet solid, m.p.: dec. > 150 °C; eluents for chromatography: dichloromethane/ethyl acetate = 8 : 2; yield = 98% (38.5 mg); ^1^H-NMR (600 MHz, CDCl_3_, 25 °C) *δ*, ppm: 8.30 (d, *J* = 8.9 Hz, 2H), 7.89 (d, *J* = 8.9 Hz, 2H), 7.80 (d, *J* = 9.2 Hz, 1H), 6.94 (dd, *J*_1_ = 9.2 Hz, *J*_2_ = 2.3 Hz 1H), 6.36 (d, *J* = 2.3 Hz, 1H), 3.43–3.39 (m, 4H), 3.31 (t, *J* = 4.6 Hz, 4H), 1.82 (q, *J* = 4.6 Hz, 4H), 1.74–1.63 (m, 8H, three signals overlapped); ^13^C-NMR (150 MHz, CDCl_3_, 25 °C) *δ*, ppm: 157.3 (C), 155.5 (C, two signals overlapped), 146.7 (C), 136.6 (C), 124.8 (CH), 122.4 (CH), 118.8 (CH), 108.2 (CH), 102.4 (CH), 54.5 (CH_2_), 48.7 (CH_2_), 26.4 (CH_2_), 25.5 (CH_2_), 24.4 (CH_2_), 24.3 (CH_2_); ESI MS (ES^+^) *m*/*z*: 394 [M + H]^+^, 416 [M + Na]^+^; HRMS (ES^+^) *m*/*z*: [M + H]+ calculated for C_22_H_28_N_5_O_2_ 394.2243; found 394.2246.

#### 1,1′-(4-((4-Bromophenyl)diazenyl)-1,3-phenylene)dipiperidine (6b)

Orange solid, m.p.: 122.4–125.7 °C, eluents for chromatography: ethyl acetate/hexane = 9 : 1; yield = 97% (41.4 mg); ^1^H-NMR (600 MHz, CDCl_3_, 25 °C) *δ*, ppm: 7.72 (m, 3H, Two signals overlapped), 7.58 (d, *J* = 8.1 Hz, 2H), 6.52 (s, 1H), 6.43 (s, 1H), 3.35 (s, 4H), 3.24 (s, 4H), 1.81 (s, 4H), 1.73–1.67 (m, 4H), 1.67–1.59 (m, 4H); _13_C-NMR (150 MHz, CDCl_3_, 25 °C) *δ*, ppm: 154.8 (C), 154.0 (C), 152.3 (C), 136.5 (C), 132.1 (CH), 123.8 (CH), 122.8 (C), 118.2 (CH), 108.5 (CH), 103.6 (CH), 54.6 (CH_2_), 49.1 (CH_2_), 26.4 (CH_2_), 25.5 (CH_2_), 24.3 (CH_2_), 22.7 (CH_2_); ESI MS (ES^+^) *m*/*z*: 427 (^79^Br) [M + H]^+^, 429 (^81^Br) [M + H]^+^, 449 (^79^Br) [M + Na]^+^, 451 (^81^Br) [M + Na]^+^; HRMS (ES^+^) *m*/*z*: [M + H]^+^ calculated for C_22_H_28_BrN_4_ 427.1497 (^79^Br) and 429.1477 (^81^Br); found 427.1498 (^79^Br) and 429.1479 (^81^Br).

#### 1,1′-(4-((4-Methoxyphenyl)diazenyl)-1,3-phenylene)dipiperidine (6c)

Orange grease solid; eluents for chromatography: dichloromethane/ethyl acetate = 8 : 2; yield = 78% (29.5 mg); ^1^H-NMR (300 MHz, CDCl_3_, 25 °C) *δ*, ppm: 7.86 (d, *J* = 8.4 Hz, 2H), 7.68 (d, *J* = 8.4 Hz, 1H), 6.98 (d, *J* = 8.8 Hz, 2H), 6.58–6.45 (m, 2H, two signals overlapped), 3.87 (s, 3H), 3.30 (br. s, 4H), 3.21 (br. s, 4H), 1.88–1.77 (m, 4H), 1.77–1.55 (m, 8H); ^13^C-NMR (150 MHz, CDCl_3_, 25 °C) *δ*, ppm: 160.8 (C), 154.3 (C), 153.0 (C), 147.8 (C), 136.9 (C), 124.0 (CH), 117.8 (CH), 114.1 (CH), 108.7 (CH), 104.3 (CH), 55.5 (CH_3_), 54.6 (CH_2_), 49.4 (CH_2_), 26.4 (CH_2_), 25.6 (CH CH_2_, two signals overlapped), 24.3; ESI MS (ES^+^) *m*/*z*: 379 [M + H]^+^, 401 [M + Na]^+^; HRMS (ES^+^) *m*/*z*: [M + H]^+^ calculated for C_23_H_31_N_4_O 379.2498; found 379.2500.

#### 4,4′-(4-((4-Nitrophenyl)diazenyl)-1,3-phenylene)dimorpholine (7a)

Dark violet solid, m.p.: dec > 210 °C; eluents for chromatography: ethyl acetate/dichloromethane = 6 : 4; yield = 97% (38.5 mg); ^1^H-NMR (400 MHz, CDCl_3_, 25 °C) *δ*, ppm: 8.33 (d, *J* = 8.9 Hz, 2H), 7.88 (d, *J* = 8.9 Hz, 2H), 7.82 (s, 1H), 6.55 (dd, *J*_1_ = 9.6 Hz, *J*_2_ = 2.6 Hz 1H), 6.39 (d, *J* = 2.6 Hz, 1H), 3.98–3.92 (m, 4H), 3.90–3.84 (m, 4H), 3.41–3.32 (m, 8H, two signals overlapped); ^13^C-NMR (150 MHz, CDCl_3_, 25 °C) *δ*, ppm: 156.8 (C), 155.3 (C), 153.8 (C), 147.4 (C), 137.4 (C), 124.8 (CH), 122.6 (CH), 118.9 (CH), 108.3 (CH), 102.5 (CH), 67.1 (CH_2_), 66.5 (CH_2_), 53.4 (CH_2_), 47.5 (C CH_2_H2); ESI MS (ES^+^) *m*/*z*: 398 [M + H]^+^, 420 [M + Na]^+^; HRMS (ES^+^) *m*/*z*: [M + H]^+^ calculated for C_20_H_24_N_5_O4 398.1828; found 398.1829.

#### 4,4′-(4-((4-Bromophenyl)diazenyl)-1,3-phenylene)dimorpholine (7b)

Orange-red solid, m.p.: 142.4–147.5 °C; eluents for chromatography: ethyl acetate/dichloromethane = 6 : 4; yield = 96% (41.5 mg); ^1^H-NMR (300 MHz, CDCl_3_, 25 °C) *δ*, ppm: 7.76 (d, *J* = 9.1 Hz, 1H), 7.69 (d, *J* = 9.1 Hz, 2H), 7.59 (d, *J* = 8.5 Hz, 2H), 6.56 (d, *J* = 8.5 Hz, 1H), 6.43 (s, 1H), 3.95 (s, 4H), 3.87 (t, *J* = 5.05, 4H), 3.35–3.25 (m, 8H, two signals overlapped); ^13^C-NMR (150 MHz, CDCl_3_, 25 °C) *δ*, ppm: 154.4 (C), 152.5 (C), 151.9 (C), 137.2 (C), 132.3 (CH), 123.8 (CH), 123.7 (C), 118.5 (CH), 108.6 (CH), 103.2 (CH), 67.1 (CH_2_), 66.6 (CH_2_), 53.4 (CH_2_), 47.9 (CH_2_); ESI MS (ES^+^) *m*/*z*: 431 (^79^Br) [M + H]^+^, 433 (^81^Br) [M + H]^+^, 453 (^79^Br) [M + Na]^+^, 455 (^81^Br) [M + Na]^+^; HRMS (ES^+^) *m*/*z*: [M + H]^+^ calculated for C_20_H_24_BrN_4_O_2_ 431.1083 (^79^Br) and 433.1062 (^81^Br); found 31.1086 (^79^Br) and 433.1064 (^81^Br).

#### 4,4′-(4-((4-Methoxyphenyl)diazenyl)-1,3-phenylene)dimorpholine (7c)

Orange solid, m.p.: 136.4–138.8 °C; yield = 78% (29.8 mg); eluents for chromatography: ethyl acetate/dichloromethane = 7 : 3; ^1^H-NMR (600 MHz, CDCl_3_, 25 °C) *δ*, ppm: 7.82 (d, *J* = 8.8 Hz, 2H), 7.71 (d, *J* = 8.8 Hz, 1H), 6.99 (d, *J* = 8.5 Hz, 2H), 6.57 (d, *J* = 8.5 Hz, 1H), 6.47 (s,1H), 3.95 (t, *J* = 4.44, 4H), 3.88–3.86 (m, 7H, two signals overlapped), 3.30–3.26 (m, 8H, Two signals overlapped): ^13^C-NMR (150 MHz, CDCl_3_, 25 °C) *δ*, ppm: 161.1 (C), 153.7 (C), 151.6 (C), 147.6 (C), 137.7 (C), 124.1 (CH), 118.1 (CH), 114.2 (CH), 108.8 (CH), 103.6 (CH), 67.2 (CH_2_), 66.7 (CH_2_), 55.5 (CH_3_), 53.4 (CH_2_), 48.3 (CH_2_); ESI MS (ES^+^) *m*/*z*: 383 [M + H]^+^, 405 [M + Na]^+^; HRMS (ES^+^) *m*/*z*: [M + H]^+^ calculated for C_21_H_27_N_4_O_3_ 383.2083; found 383.2085.

#### 1,1′-(4-((4-Nitrophenyl)diazenyl)-1,3-phenylene)dipyrrolidine (8a)

Green solid, m.p.: dec. > 195 °C; eluents for chromatography: dichloromethane/ethyl acetate = 8 : 2; yield = 95% (34.7 mg); ^1^H-NMR (600 MHz, CDCl_3_, 25 °C) *δ*, ppm: 8.24 (d, *J* = 8.2 Hz, 2H), 7.99 (s, 1H), 7.69 (s, 2H), 6.14 (s, 1H), 5.63 (s, 1H), 3.72 (s, 4H), 3.43 (s, 4H), 2.09–2.01 (m, 8H, two signals overlapped); ^13^C-NMR (150 MHz, CDCl_3_, 25 °C) *δ*, ppm: 158.4 (C), 152.1 (C), 150.5 (C), 145.0 (C), 134.5 (C), 124.8 (CH), 121.4 (CH), 119.0 (CH), 105.5 (CH), 94.5 (CH), 52.6 (CH_2_), 47.8 (CH_2_), 25.9 (CH_2_), 25.4 (CH_2_); ESI MS (ES^+^) *m*/*z*: 366 [M + H]^+^, 388 [M + Na]^+^; HRMS (ES^+^) *m*/*z*: [M + H]^+^ calculated for C_20_H_24_N_5_O_2_ 366.1930; found 366.1932.

#### 1,1′-(4-((4-Bromophenyl)diazenyl)-1,3-phenylene)dipyrrolidine (8b)

Orange-red solid, m.p.: 189.4–192.4 °C; eluents for chromatography: dichloromethane/ethyl acetate = 9 : 1; yield = 77% (30.7 mg); ^1^H-NMR (300 MHz, CDCl_3_, 25 °C) *δ*, ppm: 7.92 (d, *J* = 8.7 Hz, 1H), 7.57 (d, *J* = 8.6 Hz, 2H), 7.52 (d, *J* = 8.6 Hz, 2H), 6.09 (d, *J* = 8.7 Hz, 1H), 5.72 (s, 1H), 3.69 (t, *J* = 6.3 Hz, 4H), 3.39 (t, *J* = 6.3 Hz, 4H), 2.04–1.98 (m, 8H); ^13^C-NMR (150 MHz, CDCl_3_, 25 °C) *δ*, ppm: 153.0 (C), 151.2 (C), 149.5 (C), 133.0 (C), 131.8 (CH), 123.1 (CH), 120.4 (C), 118.5 (CH), 104.0 (CH), 95.1 (CH), 52.5 (CH_2_), 47.7 (C CH_2_H2), 25.9 (CH_2_), 25.4 (CH_2_); ESI MS (ES^+^) *m*/*z*: 399 (^79^Br) [M + H]^+^, 401 (^81^Br) [M + H]^+^, 421 (^79^Br) [M + Na]^+^, 423 (^81^Br) [M + Na]^+^; HRMS (ES^+^) *m*/*z*: [M + H]^+^ calculated for C_20_H_24_BrN_4_ 399.1184 (^79^Br) and 401.1164 (^81^Br); found 399.1187 (^79^Br) and 401.1166 (^81^Br).

#### 1,1′-(4-((4-Methoxyphenyl)diazenyl)-1,3-phenylene)dipyrrolidine (8c)

Orange-red solid, m.p.: 158.2–161.4 °C; eluents for chromatography: dichloromethane/ethyl acetate = 9 : 1; yield = 73% (25.5 mg); ^1^H-NMR (600 MHz, CDCl_3_, 25 °C) *δ*, ppm: 7.90 (s, 1H), 7.71 (d, *J* = 8.9 Hz, 2H), 6.96 (d, *J* = 8.9 Hz, 2H), 6.09 (s, 1H), 5.76 (s, 1H), 3.85 (s, 3H), 3.69 (t, *J* = 6.6 Hz, 4H), 3.38 (t, *J* = 6.6 Hz, 4H), 2.03–1.98 (m, 8H); ^13^C-NMR (150 MHz, CDCl_3_, 25 °C) *δ*, ppm: 159.2 (C), 150.6 (C), 149.0 (C), 148.3 (C), 132.8 (C), 123.0 (CH), 118.3 (CH), 114.0 (CH), 103.4 (CH), 95.5 (CH), 55.4 (CH_3_), 52.4 (CH_2_), 47.6 (CH_2_), 25.9 (CH_2_), 25.4 (CH_2_); ESI MS (ES^+^) *m*/*z*: 351 [M + H]^+^; HRMS (ES^+^) *m*/*z*: [M + H]^+^ calculated for C_21_H_27_N4O 351.2185; found 351.2187.

#### 1-(2,4-Dimethoxyphenyl)-2-(4-nitrophenyl)diazene (9a)

Orange-red solid, m.p.: dec. > 190 °C; petroleum ether/diethyl ether = 1 : 1; yield = 77% (22.1 mg); ^1^H-NMR (600 MHz, CDCl_3_, 25 °C) *δ*, ppm: 8.34 (d, *J* = 8.4 Hz, 2H), 7.95 (d, *J* = 8.4 Hz, 2H), 7.81 (d, *J* = 8.7 Hz, 1H), 6.61 (s, 1H), 6.58 (d, *J* = 8.7 Hz, 1H), 4.04 (s, 3H), 3.92 (s, 3H); ^13^C-NMR (150 MHz, CDCl_3_, 25 °C) *δ*, ppm: 165.2 (C), 159.9 (C), 156.5 (C), 147.9 (C), 136.9 (C), 124.7 (CH), 123.1 (CH), 118.3 (CH), 106.2 (CH), 98.9 (CH), 56.4 (CH_3_), 55.7 (CH_3_); ESI MS (ES^+^) *m*/*z*: 288 [M + H]^+^, 310 [M + Na]^+^; HRMS (ES^+^) *m*/*z*: [M + H]^+^ calculated for C_14_H_14_N_3_O_4_ 288.0984; found 288.0985.

#### 1-(4-Bromophenyl)-2-(2,4-dimethoxyphenyl)diazene (9b)

Yellow solid, m.p.: 81.2–84.4 °C; eluents for chromatography: dichloromethane/petroleum ether = 2 : 1; yield = 26% (8.4 mg); ^1^H-NMR (400 MHz, CDCl_3_, 25 °C) *δ*, ppm: 7.77 (d, *J* = 8.9 Hz, 1H), 7.75 (d, *J* = 8.8 Hz, 2H) 7.60 (d, *J* = 8.8 Hz, 2H), 6.59 (d, *J* = 2.4 Hz, 1H), 6.55 (dd, *J*_1_ = 8.8 Hz, *J*_2_ = 2.4 Hz, 1H), 4.02 (s, 3H), 3.89 (s, 3H); ^13^C-NMR (150 MHz, CDCl_3_, 25 °C) *δ*, ppm: 164.1 (C), 159.0 (C), 151.9 (C), 136.7 (C), 132.2 (CH), 124.2 (C), 124.1(CH), 118.2 (CH), 105.8 (CH), 99.0 (CH), 56.4 (CH_3_), 55.7 (CH_3_); ESI MS (ES^+^) *m*/*z*: 321 (^79^Br) [M + H]^+^, 323 (^81^Br) [M + H]^+^, 343 (^79^Br) [M + Na]^+^, 345 (^81^Br) [M + Na]^+^, 359 (^79^Br) [M + K]^+^, 361 (^81^Br) [M + K]^+^; HRMS (ES^+^) *m*/*z*: [M + H]^+^ calculated for C_14_H_14_BrN_2_O_2_ 321.0239 (^79^Br) and 323.0218 (^81^Br); found 321.0241 (^79^Br) and 323.0220 (^81^Br).

### Variable temperature NMR experiments

#### Reaction between 1,3-dipiperidinyl benzene 1 and *para*-nitrobenzenediazonium salt 5a in CD_3_CN

The reaction was conducted directly in an NMR tube. First, 13.1 mg (5.4 × 10^−2^ mol) of compound 1 was dissolved in 0.7 mL of deuterated acetonitrile and the ^1^H-NMR spectrum was recorded. Then the probe temperature was moved to −35 °C and the spectrum of the reagent was recorded at this temperature. Then, in the NMR tube, 12.8 mg (5.4 × 10^−2^ mol) of salt 5a was quickly added and the spectrum was immediately recorded at −35 °C. Then the probe temperature was raised from −35 °C to 25 °C and ^1^H NMR spectra were recorded every 10 °C.

#### Reaction between 1,3-dipyrrolidinyl benzene 3 and *para*-methoxybenzenediazonium salt 5c in CH_2_Cl_2_

The reaction was carried out directly in an NMR tube. First, 11 mg (5.1 × 10^−2^ mol) of compound 3 was dissolved in 0.7 mL of deuterated dichloromethane and the ^1^H-NMR spectrum was recorded. Then the probe temperature was moved to −80 °C and the spectrum of the reagent was recorded. In the tube, 11.2 mg (5.1 × 10^−2^ mol) of salt 5c was quickly added and spectra were recorded every 10 °C raising the temperature from −80 °C to 25 °C.

#### Reaction between 1,3-dipyrrolidinyl benzene 3 and tetrafluoroboric acid

The reaction was conducted directly in an NMR tube. Compound 3 (5 mg, 2.3 × 10^−5^ mol) was dissolved in 0.7 mL of deuterated acetonitrile and 3 μL (2.3 × 10^−5^ mol) of etherate tetrafluoroboric acid was added at room temperature. The solution was analysed by ^1^H-NMR spectroscopy and by 2D-NMR experiments (g-COSY and g-HSQC).

## Author contributions

Gabriele Micheletti: conceptualization, investigation, and writing – original draft. Carla Boga: conceptualization, funding acquisition, supervision, and writing - original draft.

## Conflicts of interest

There are no conflicts to declare.

## Supplementary Material

RA-014-D4RA00652F-s001
