# Supplementary material for: Aryl diazonium salts and benzene derivatives bearing two electron-donor substituents: chemical and mechanistic behaviours
Source: RSC Adv. 2024 Mar 14;14(13):8665–70. doi: 10.1039/d4ra00652f (PMC10938375; doi:10.1039/d4ra00652f)
Supplement: RA-014-D4RA00652F-s001 [file RA-014-D4RA00652F-s001.pdf]

Electronic Supplementary Material (ESI) for RSC Advances.
This journal is © The Royal Society of Chemistry 2024

Aryl diazonium salts and benzene derivatives bearing two electron-donor substituents: chemical and mechanistic behaviour

Gabriele Micheletti,*[a] Carla Boga[a]

[a].Department of Industrial Chemistry, Alma Mater Studiorum – Università di Bologna, Via Gobetti 85, 40129 Bologna, Italy

* Corresponding authors: Gabriele Micheletti gabriele.micheletti3@unibo.it.

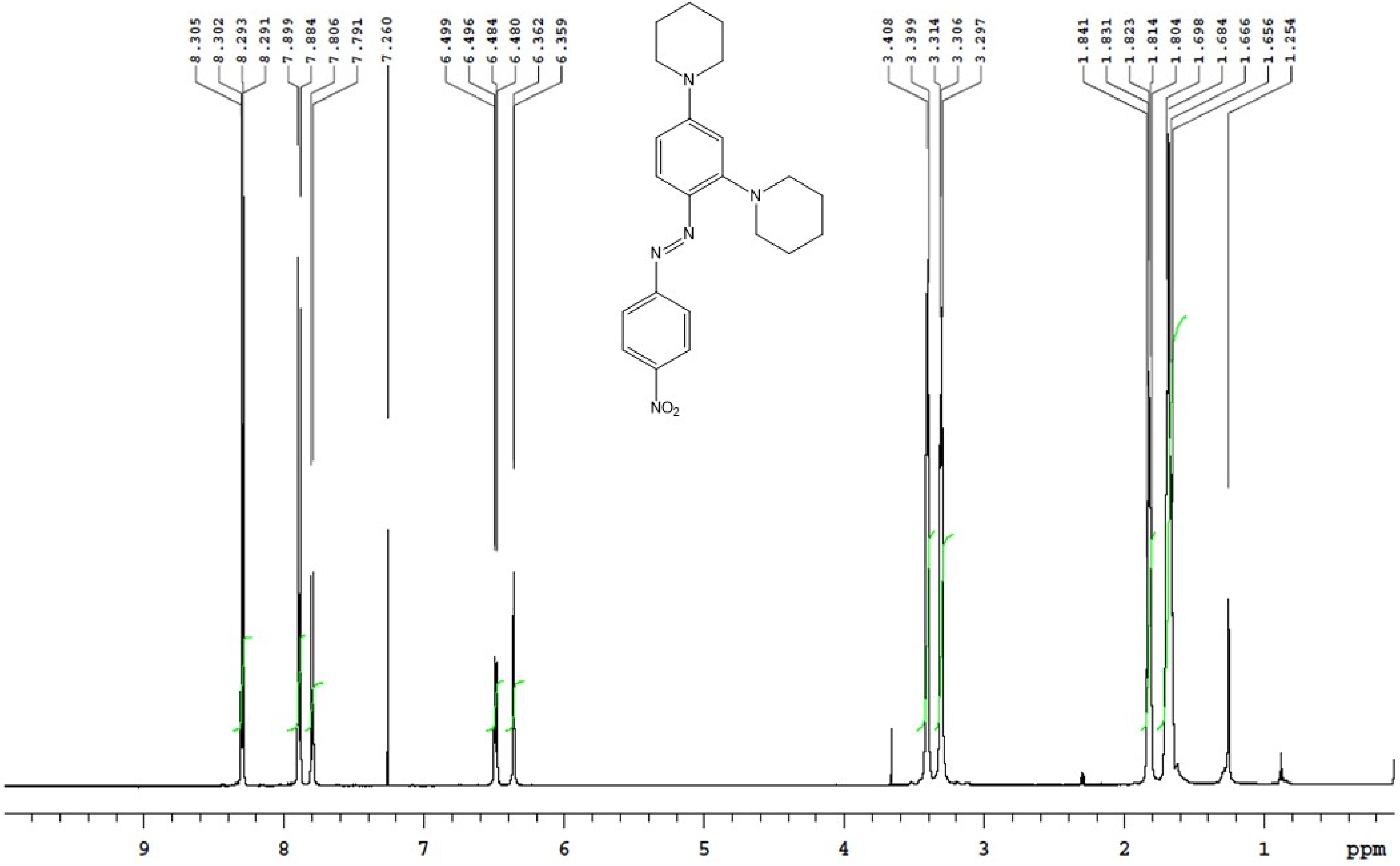

Fig. S1: ^{1}H-NMR spectrum of compound **6a** in $CDCl_3$.

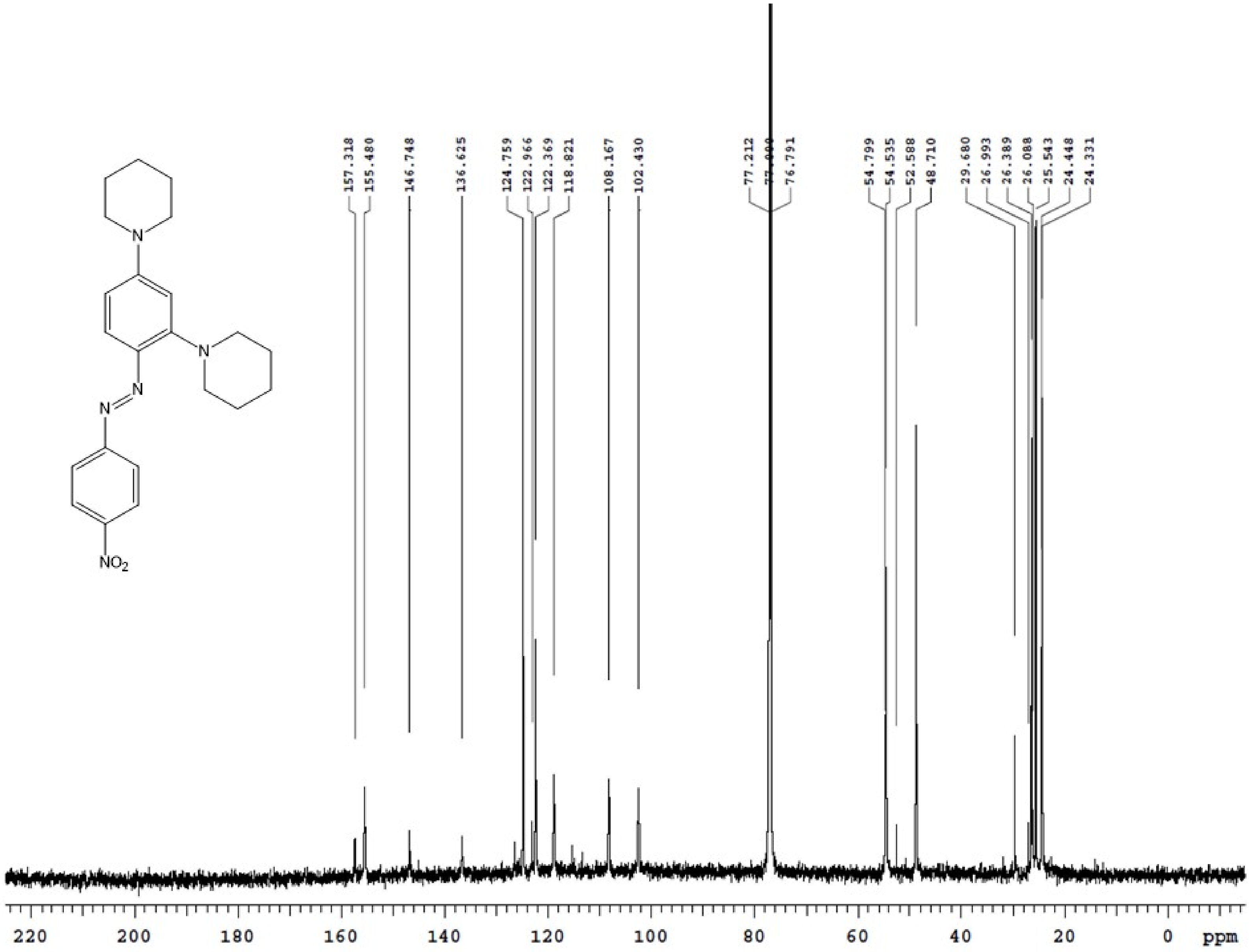

Fig. S2: ^{13}C-NMR spectrum of compound **6a** in $CDCl_3$.

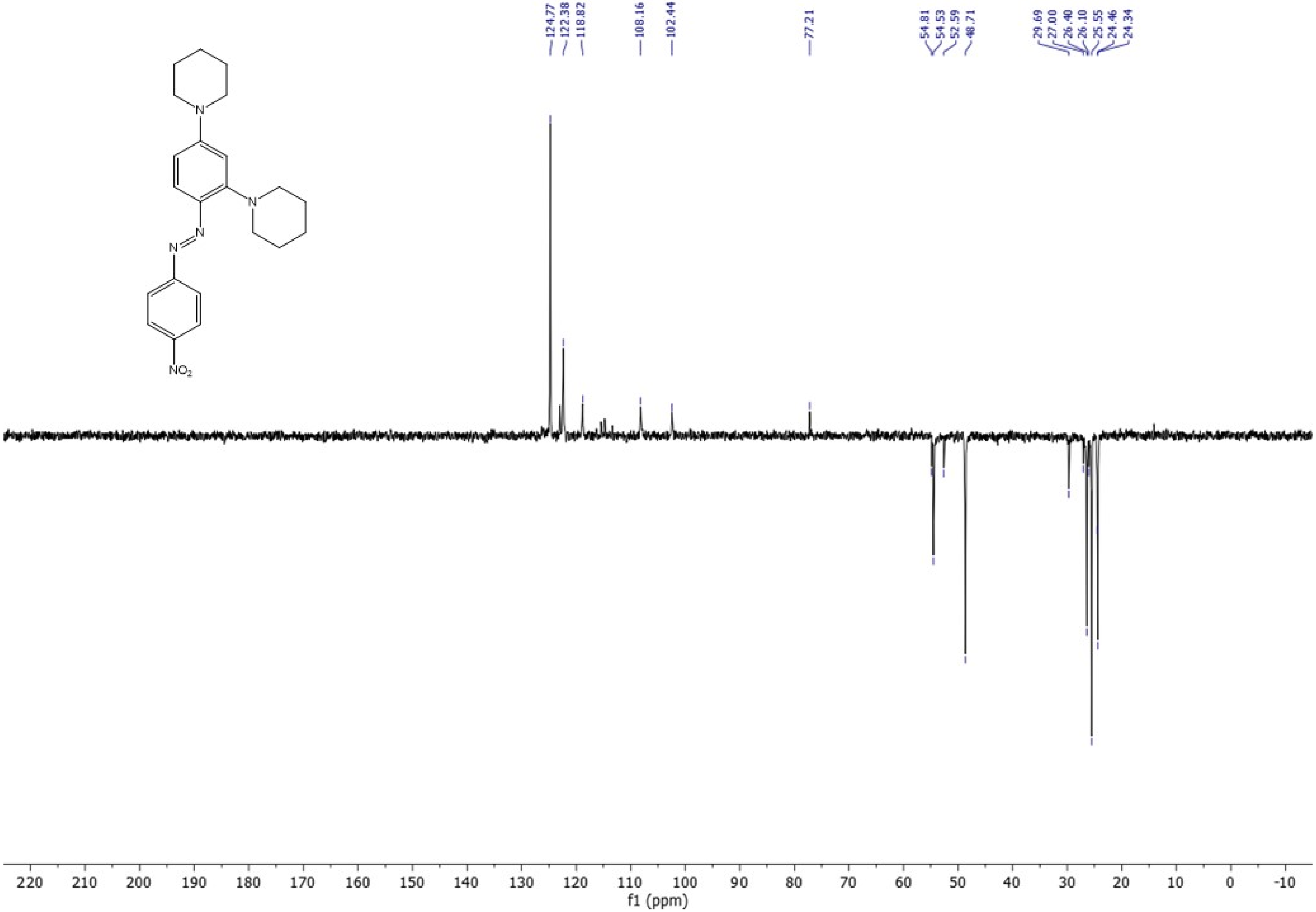

Fig. S3: DEPT spectrum of compound **6a** in $CDCl_3$.

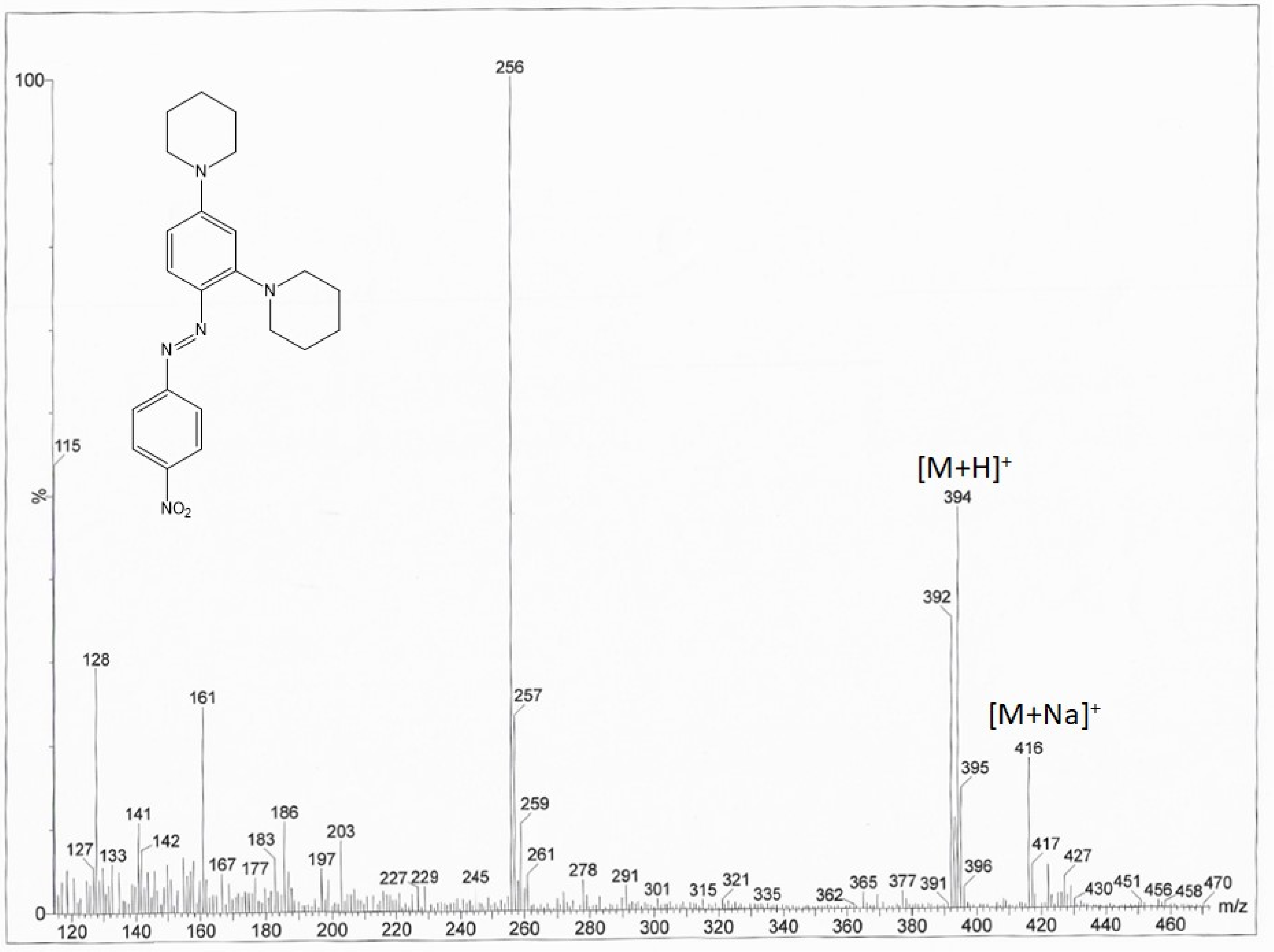

Fig. S4: ESI-MS+ (*m/z*) spectrum of compound **6a**.

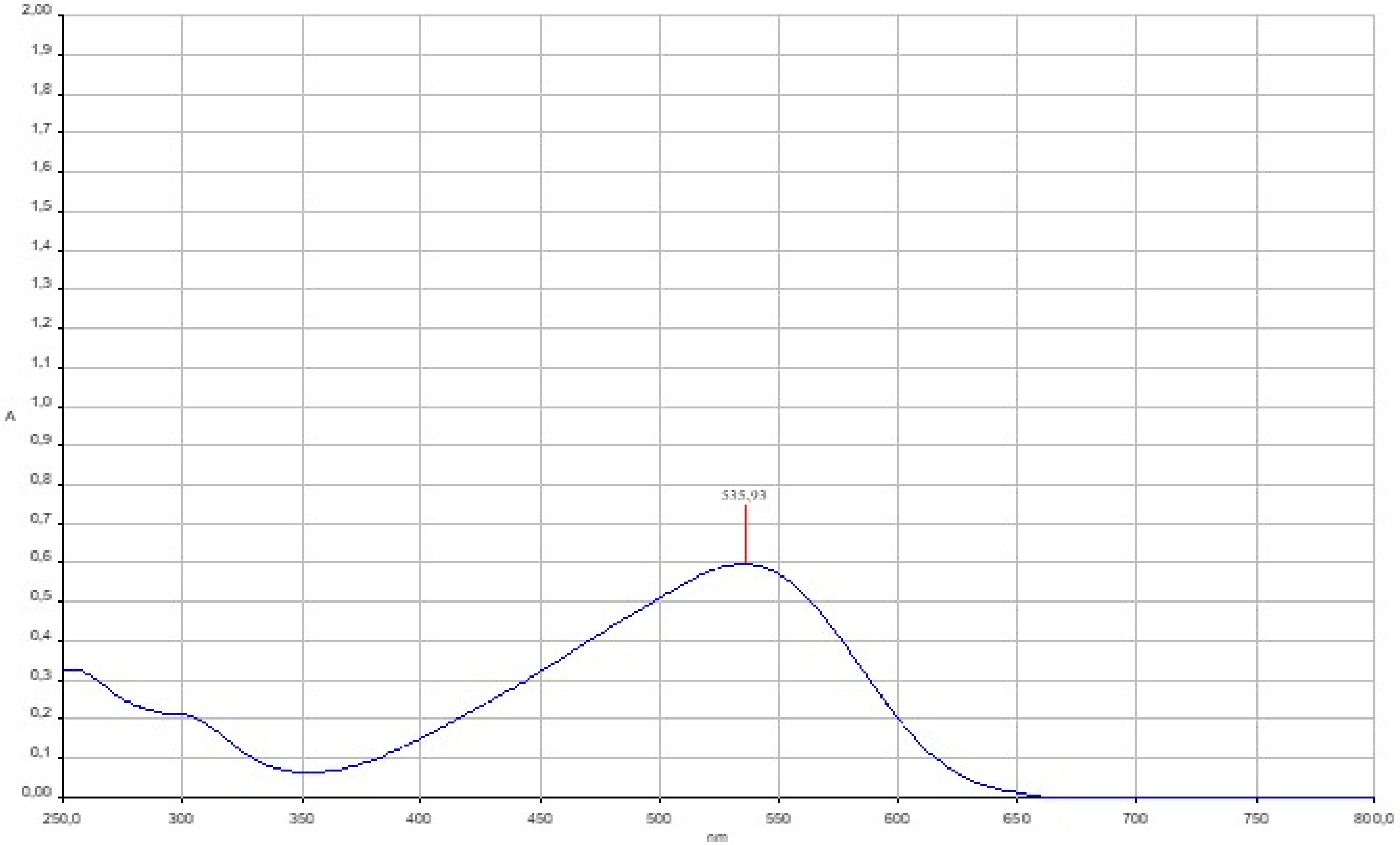

Fig. S5: UV-Vis spectrum of compound **6a** in $CHCl_3$.

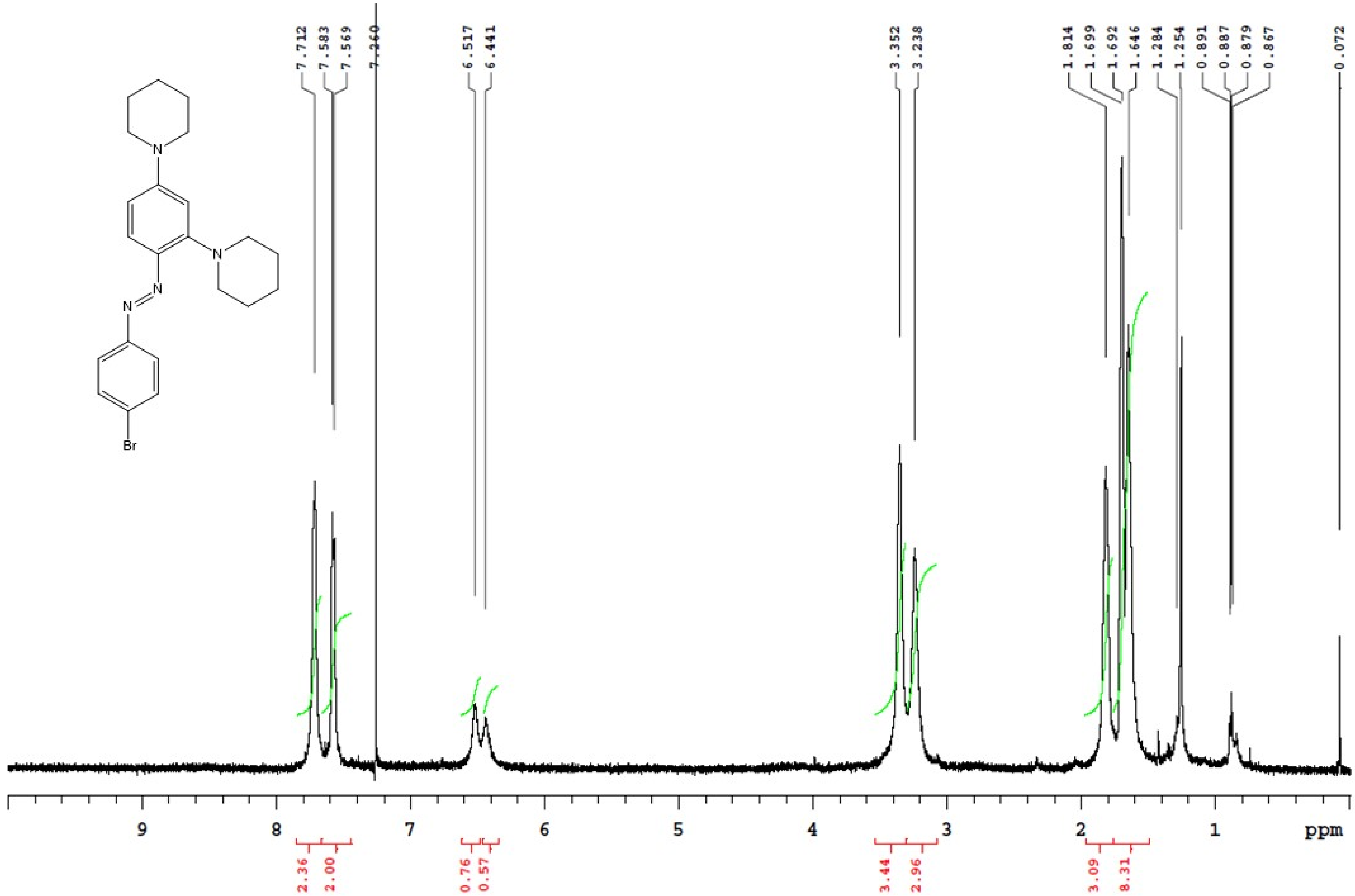

Fig. S6: ^{1}H-NMR spectrum of compound **6b** in $CDCl_3$.

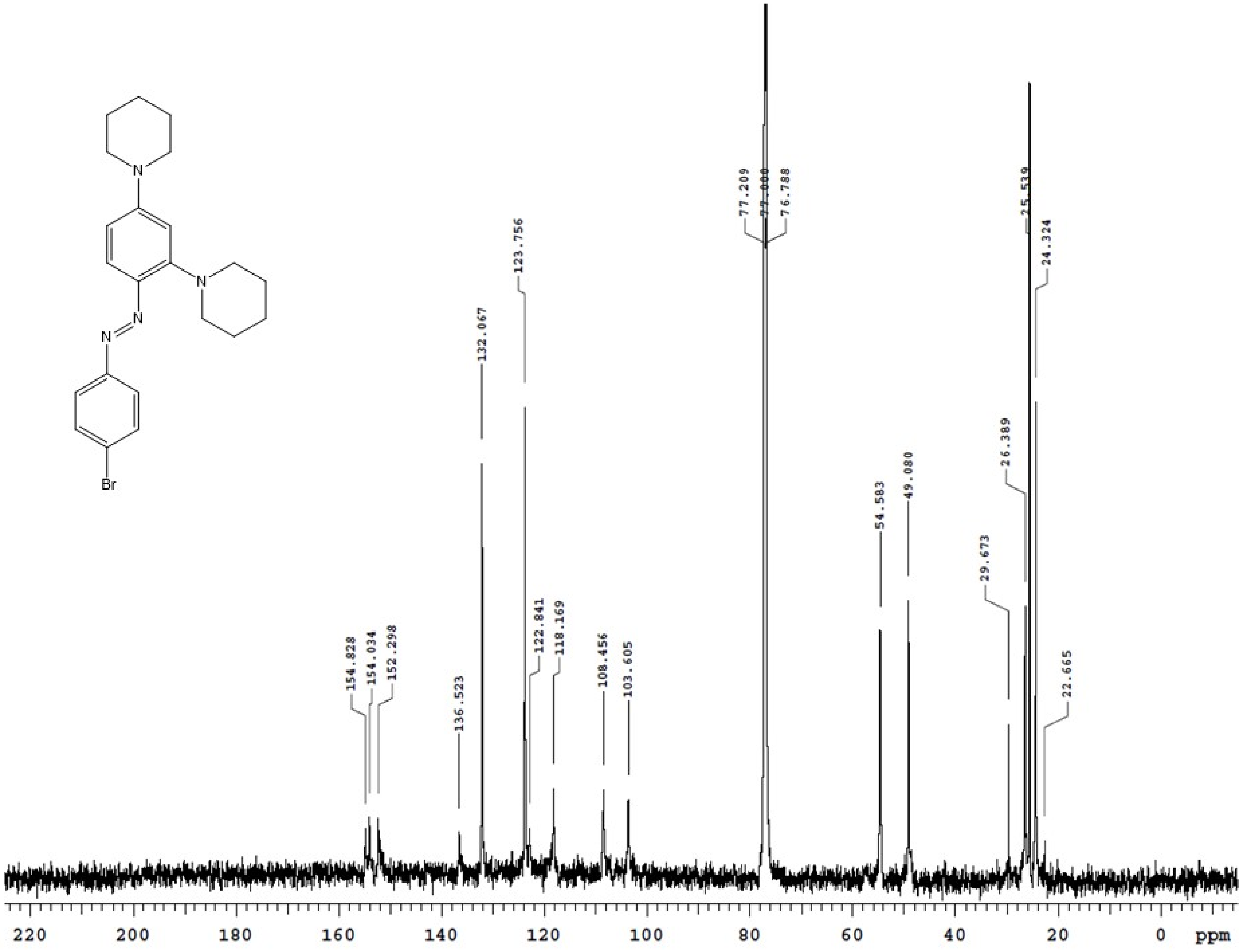

Fig. S7: ^{13}C-NMR spectrum of compound **6b** in $CDCl_3$.

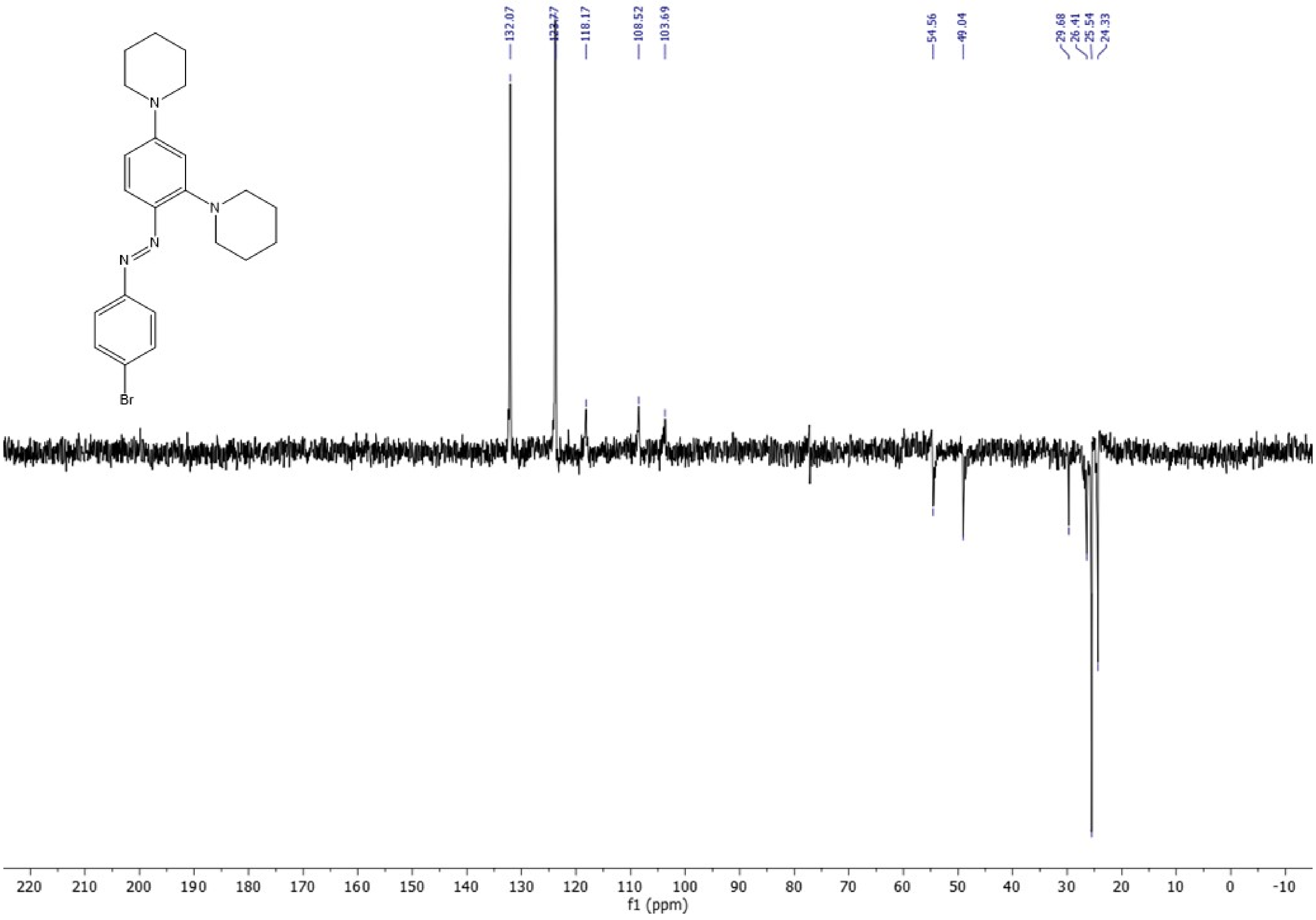

Fig. S8: DEPT spectrum of compound **6b** in $CDCl_3$.

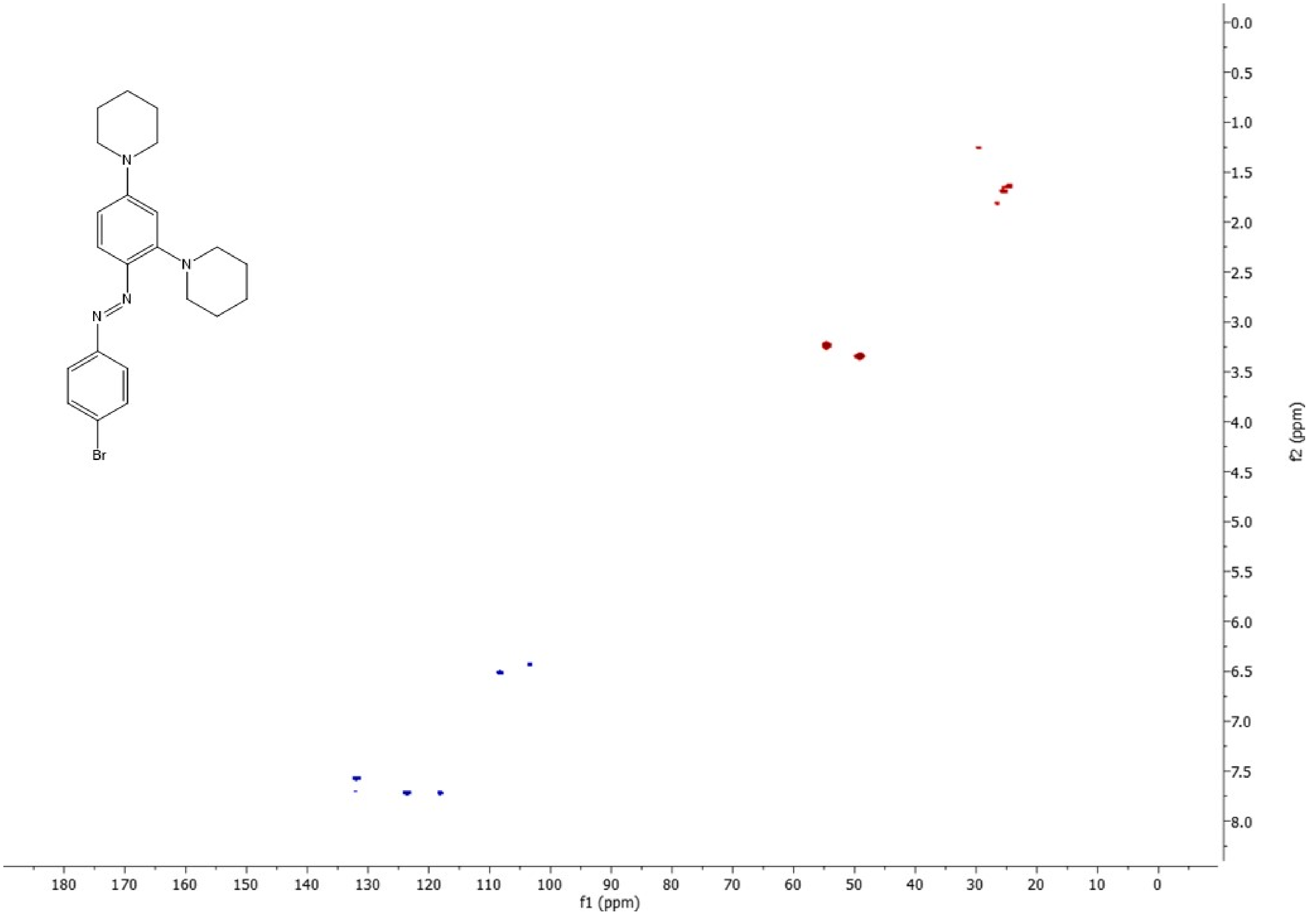

Fig. S9: HSQC spectrum of compound **6b** in $CDCl_3$.

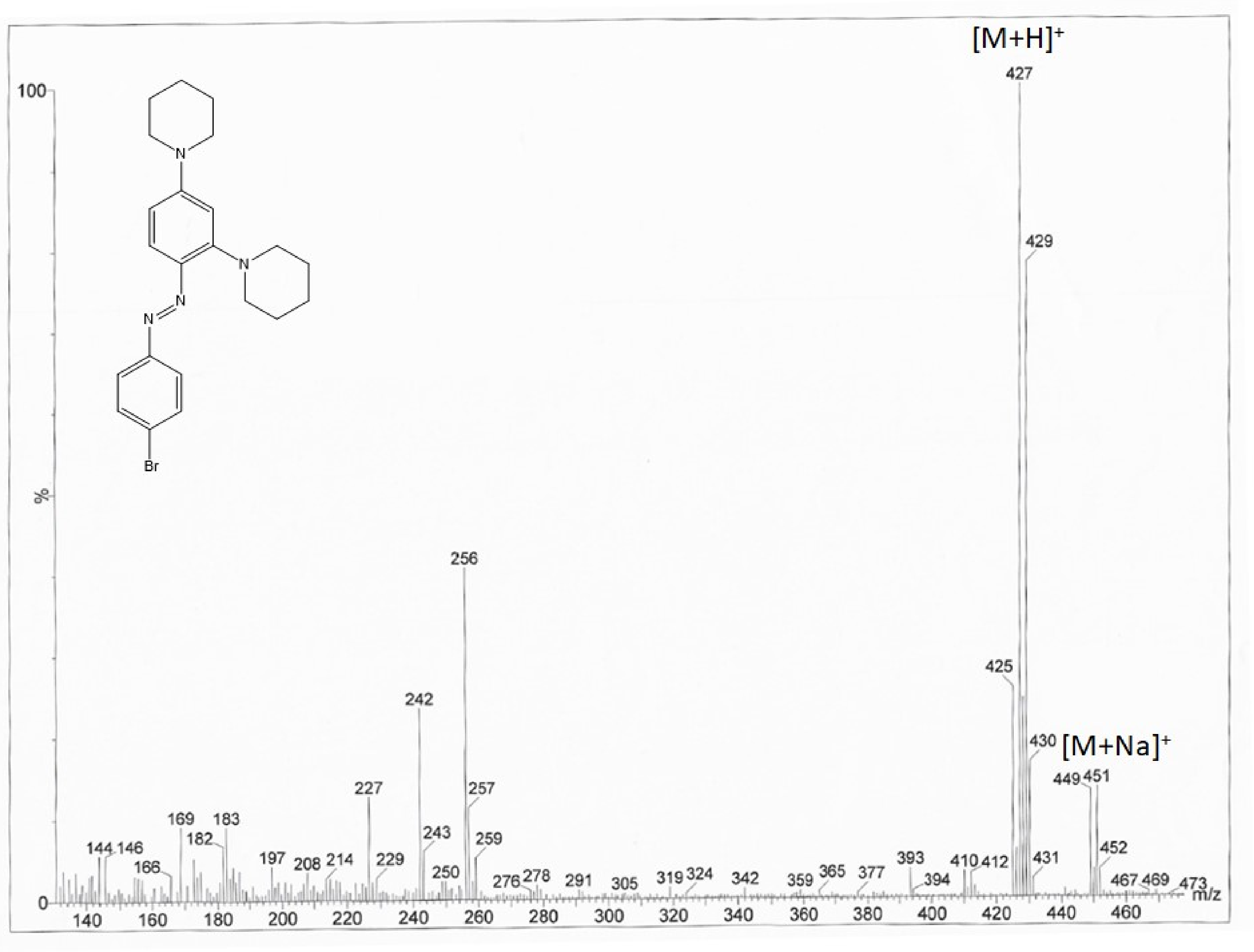

Fig. S10: ESI-MS$^+$ (*m/*z) spectrum of compound **6b**.

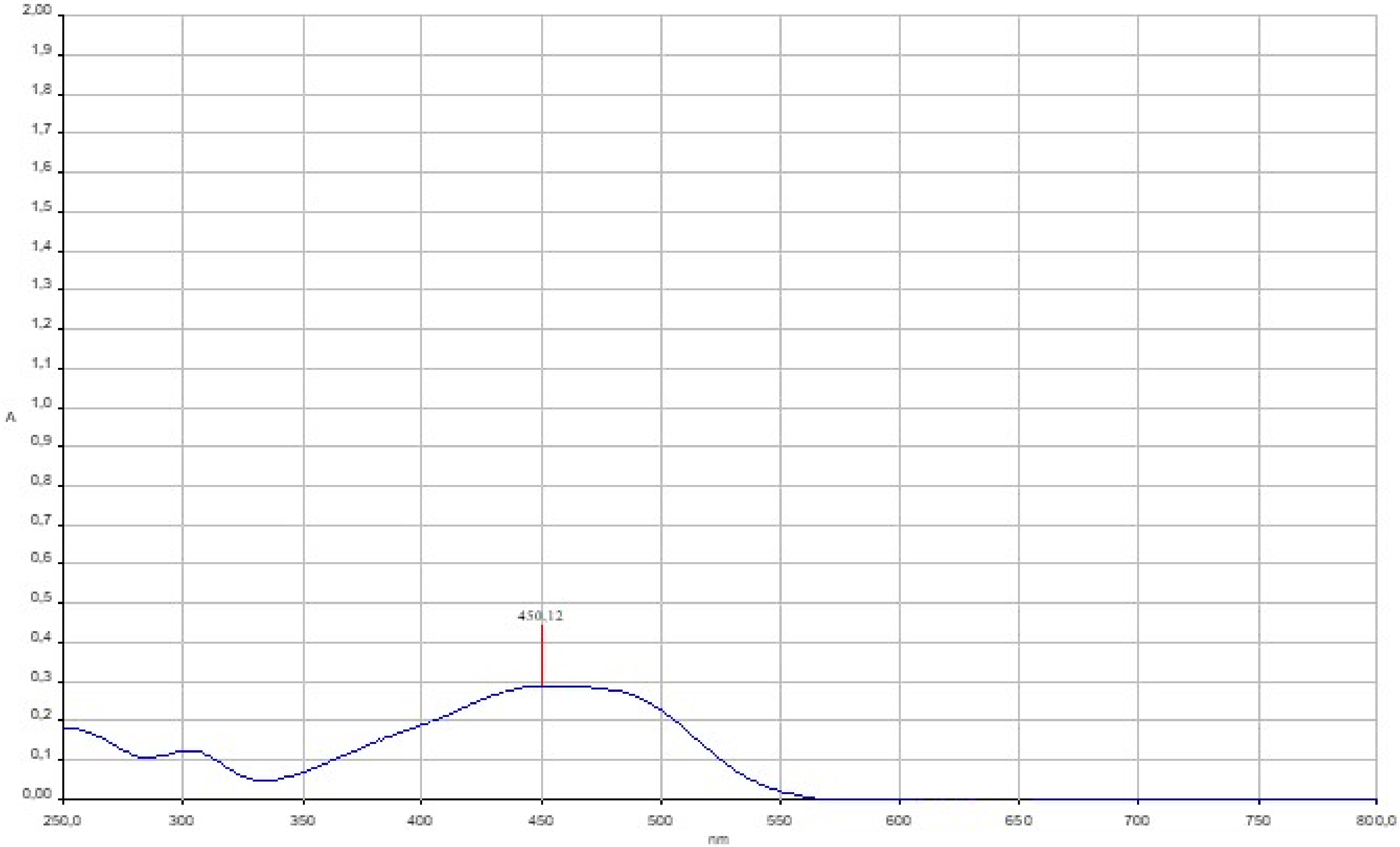

Fig. S11: UV-Vis spectrum of compound **6b** in $CHCl_3$.

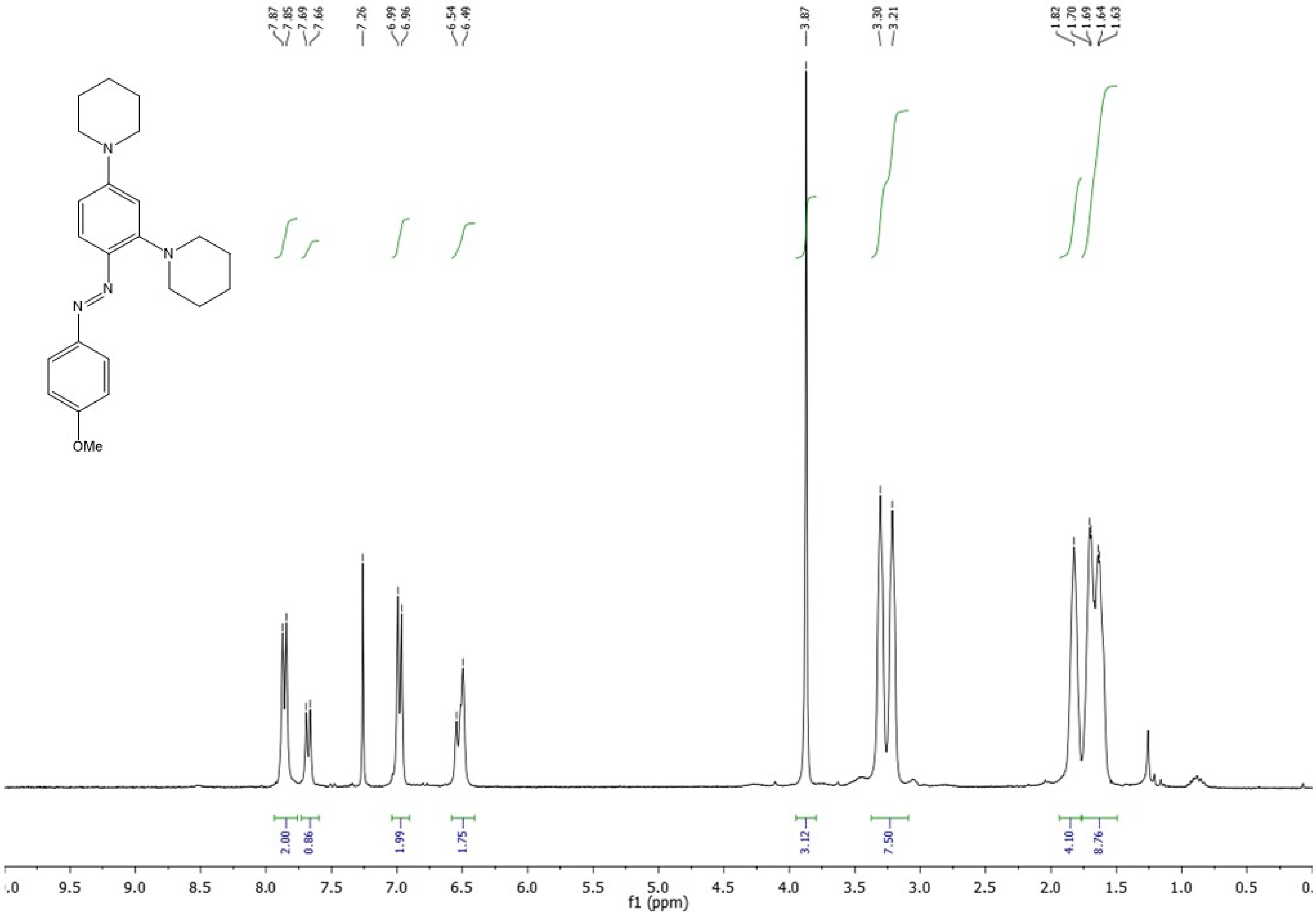

Fig. S12: ^{1}H-NMR spectrum of compound **6c** in $CDCl_3$.

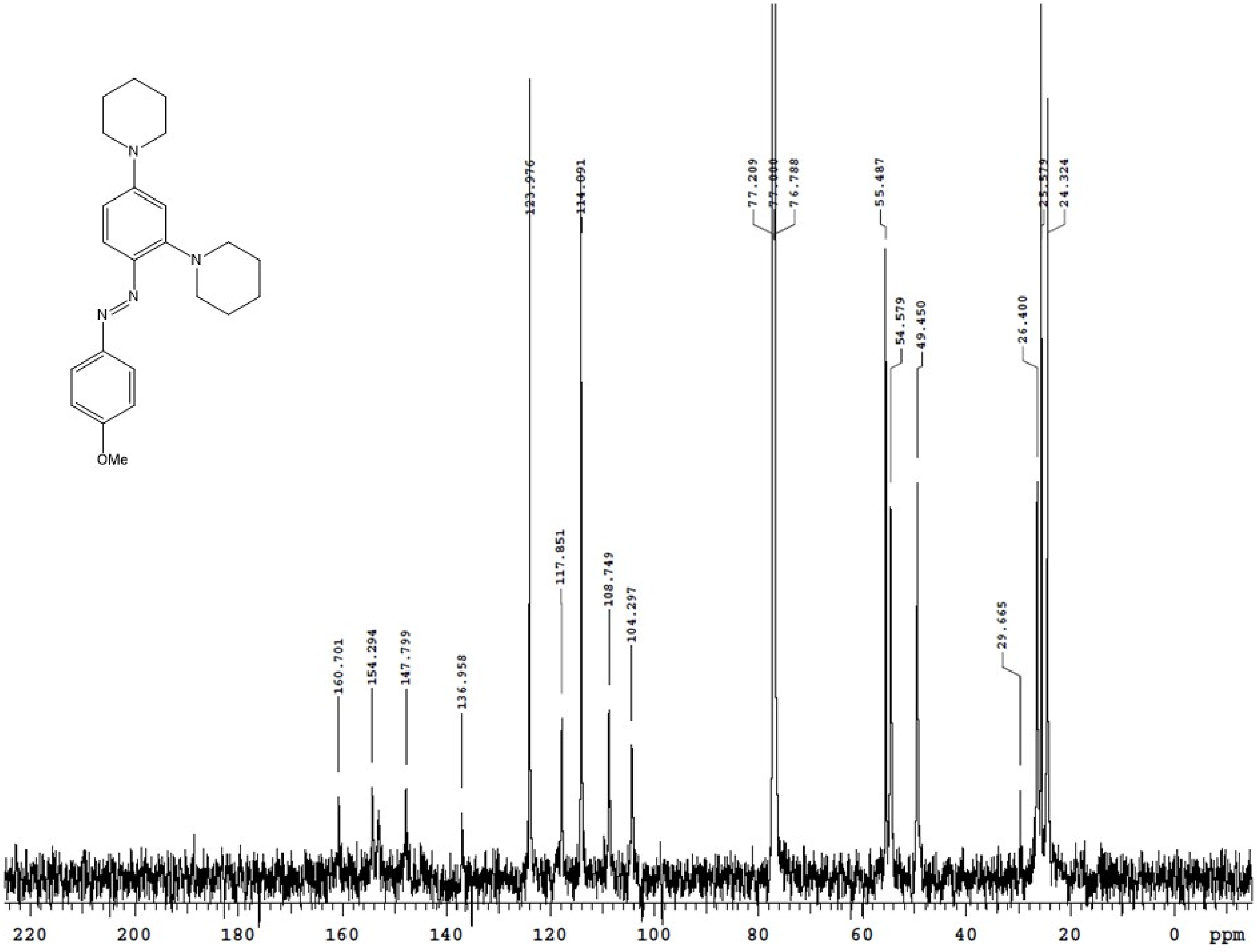

Fig. S13: ^{13}C-NMR spectrum of compound **6c** in $CDCl_3$.

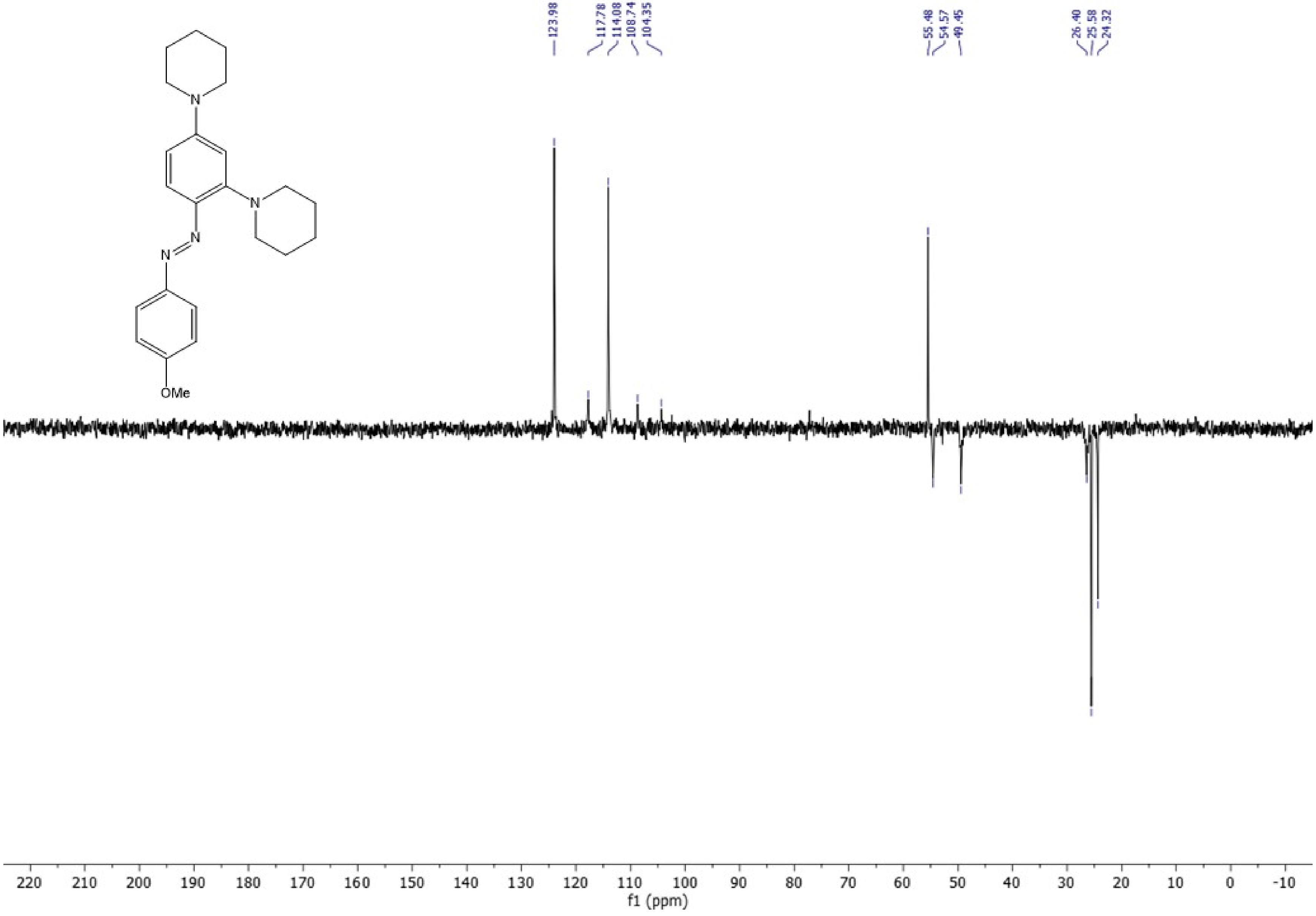

Fig. S14: DEPT spectrum of compound **6c** in $CDCl_3$.

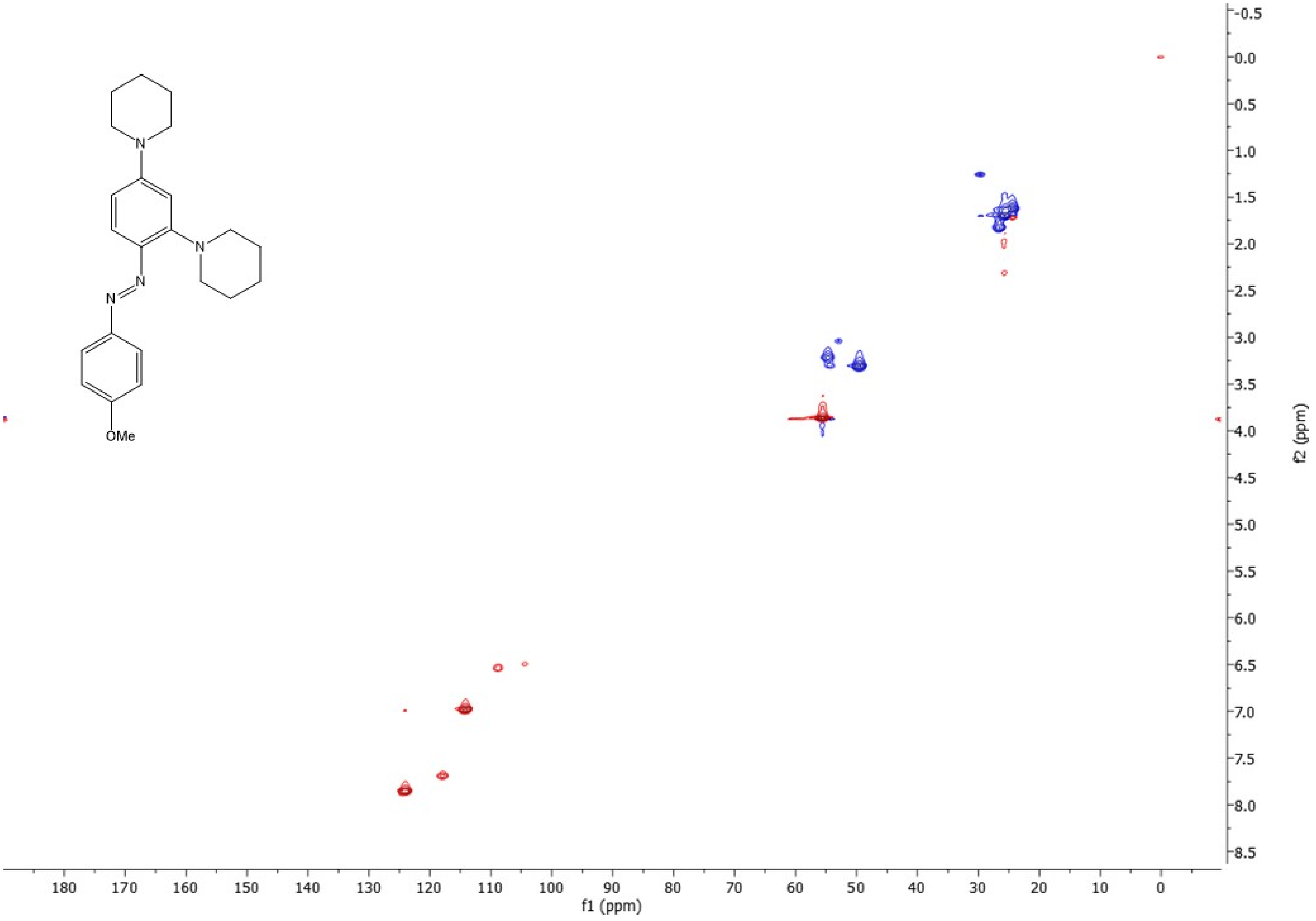

Fig. S15: HSQC-NMR spectrum of compound **6c** in $CDCl_3$.

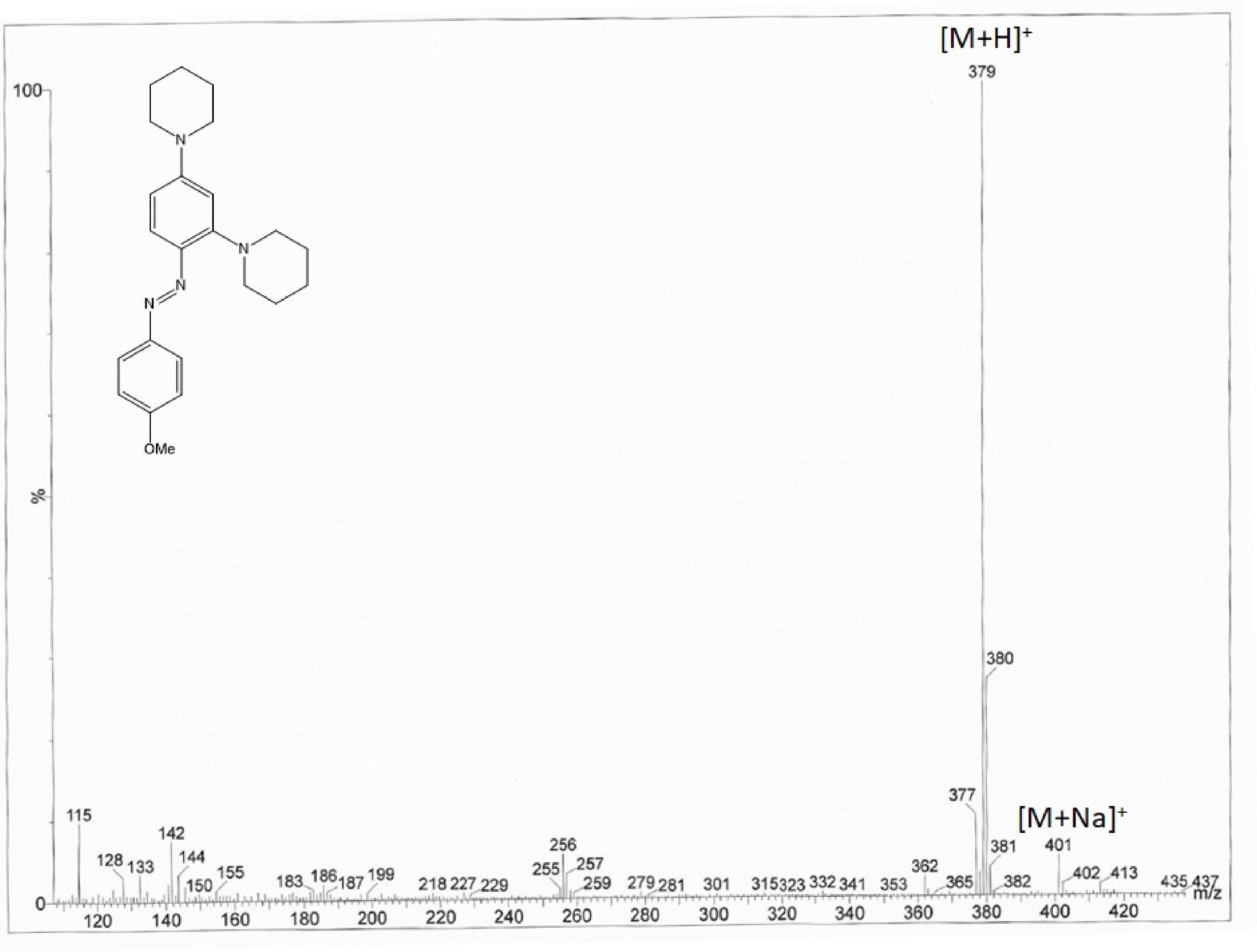

Fig. S16: ESI-MS+ (*m/*z) spectrum of compound **6c**.

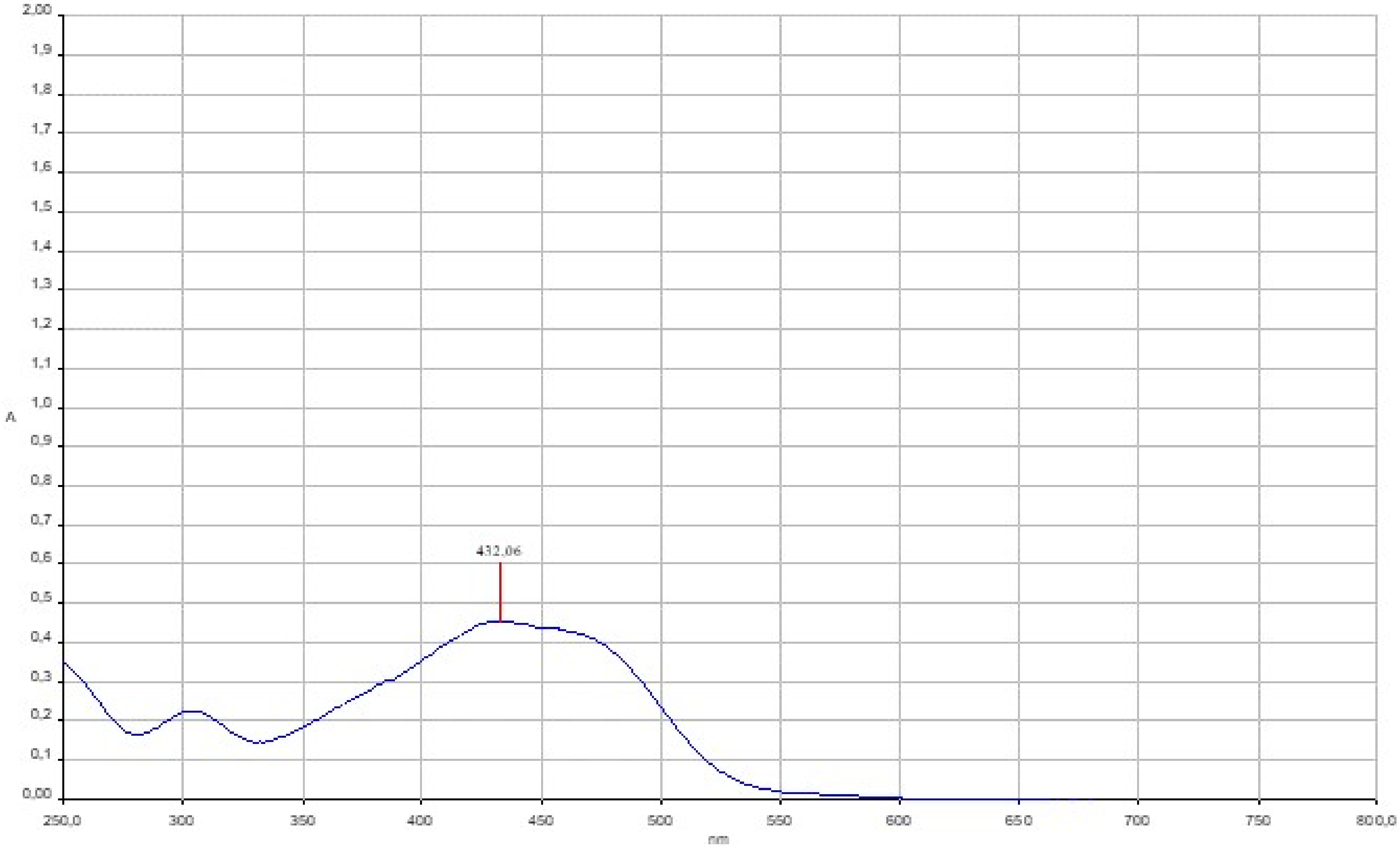

Fig. S17: UV-Vis spectrum of compound **6c** in $CHCl_3$.

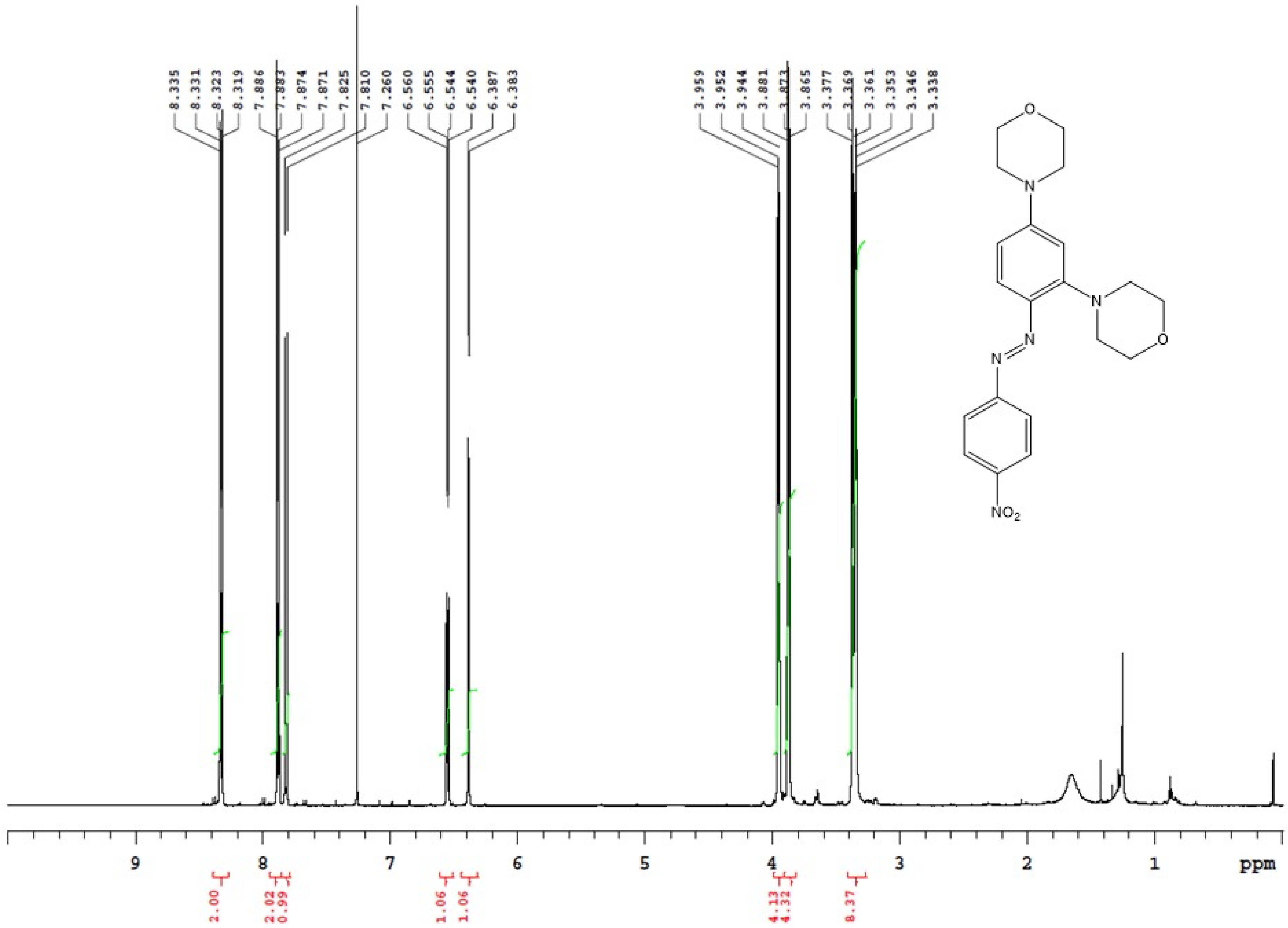

Fig. S18: ^{1}H-NMR spectrum of compound **7a** in $CDCl_3$.

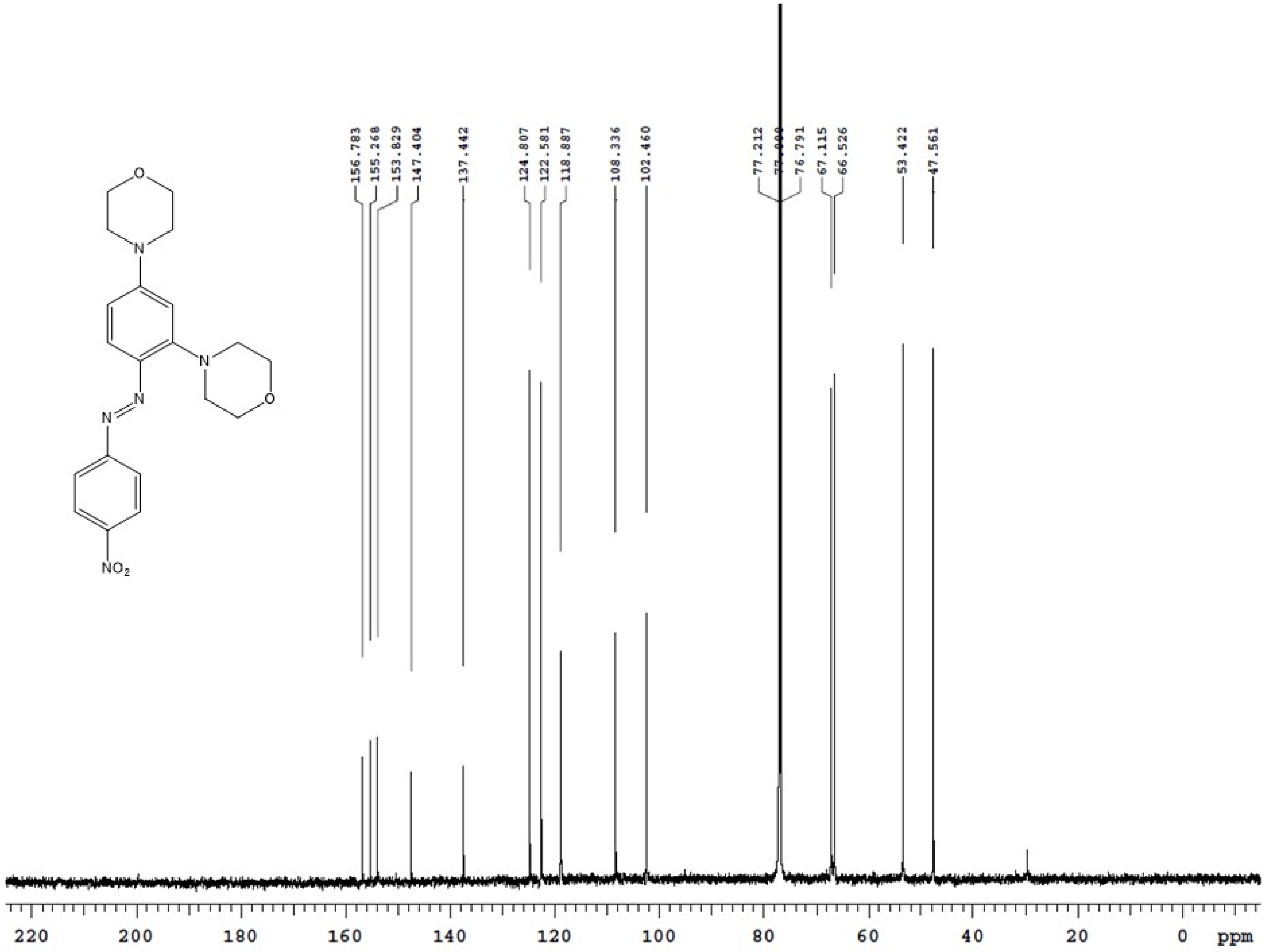

Fig. S19: ^{13}C-NMR spectrum of compound **7a** in $CDCl_3$.

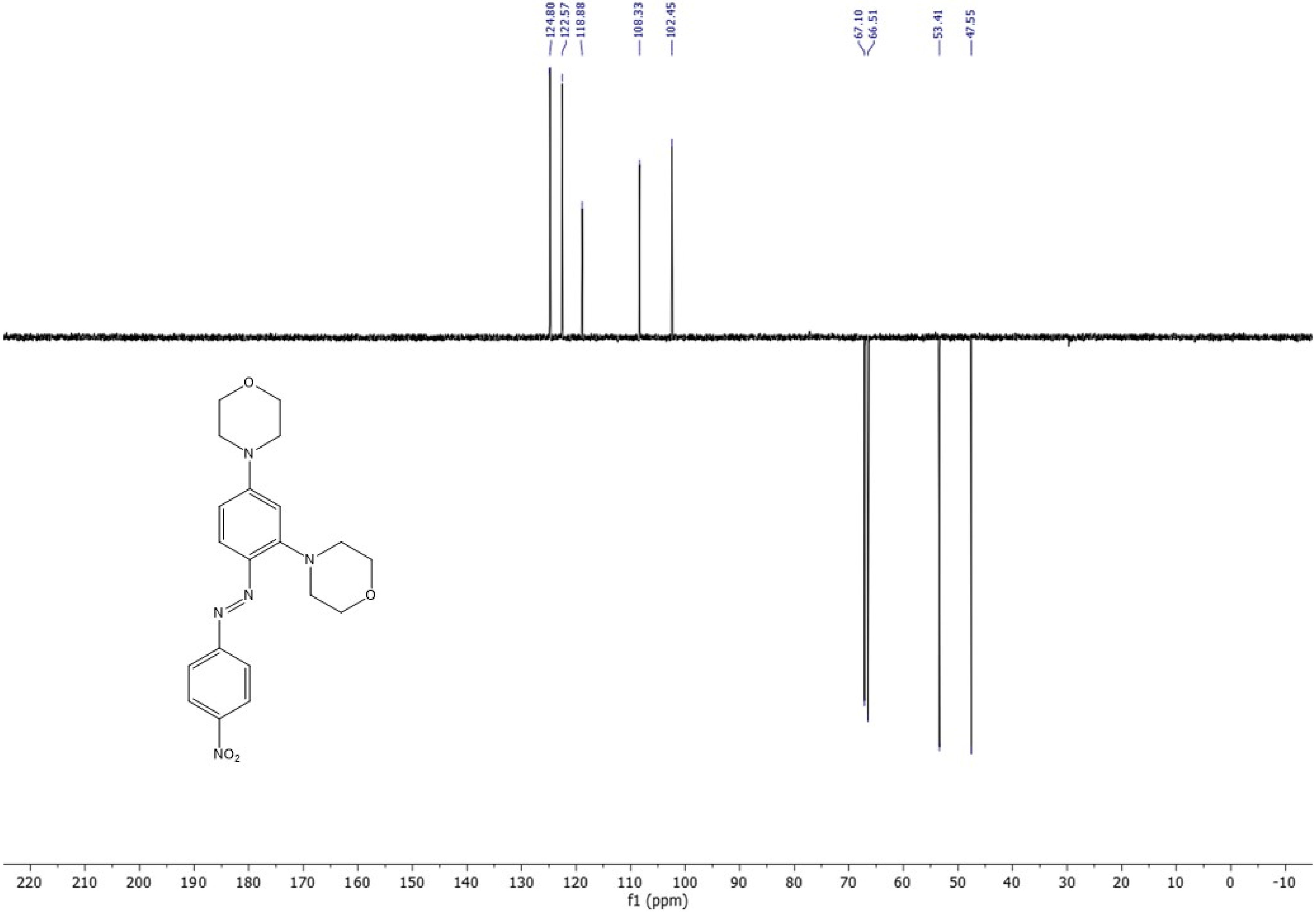

Fig. S20: DEPT spectrum of compound **7a** in $CDCl_3$.

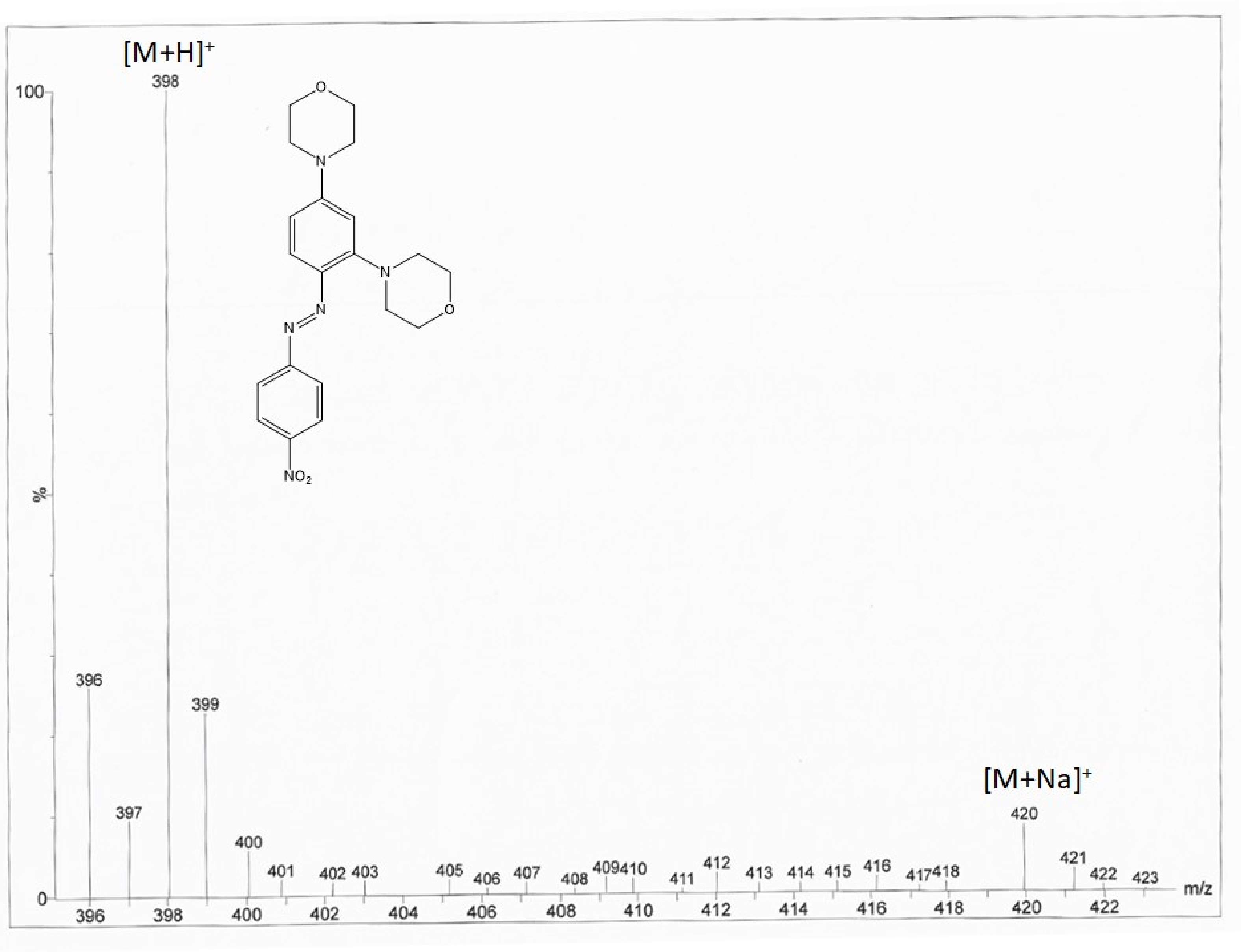

Fig. S21: ESI-MS+ (*m/*z) spectrum of compound **7a**.

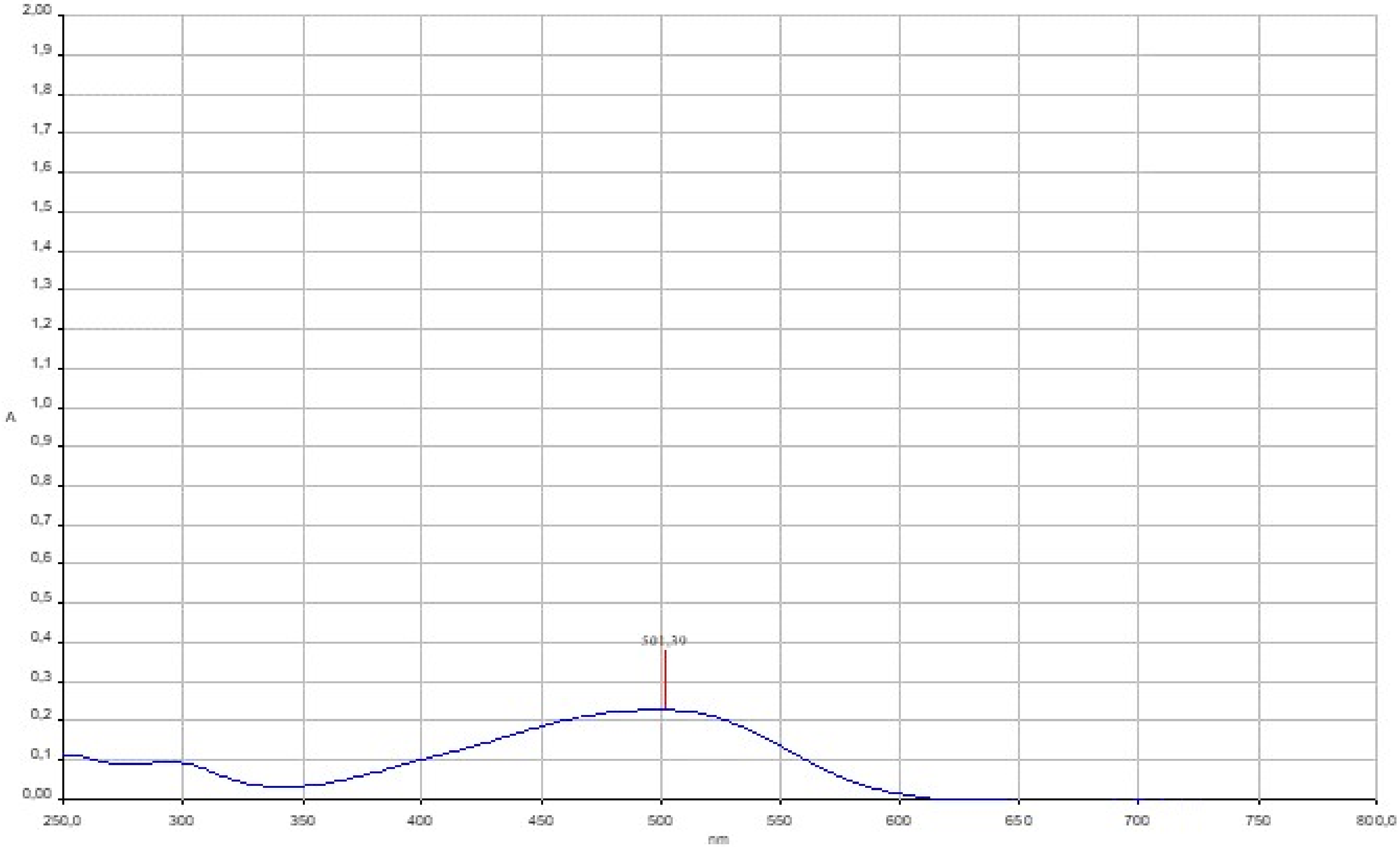

Fig. S22: UV-Vis spectrum of compound **7a** in $CHCl_3$.

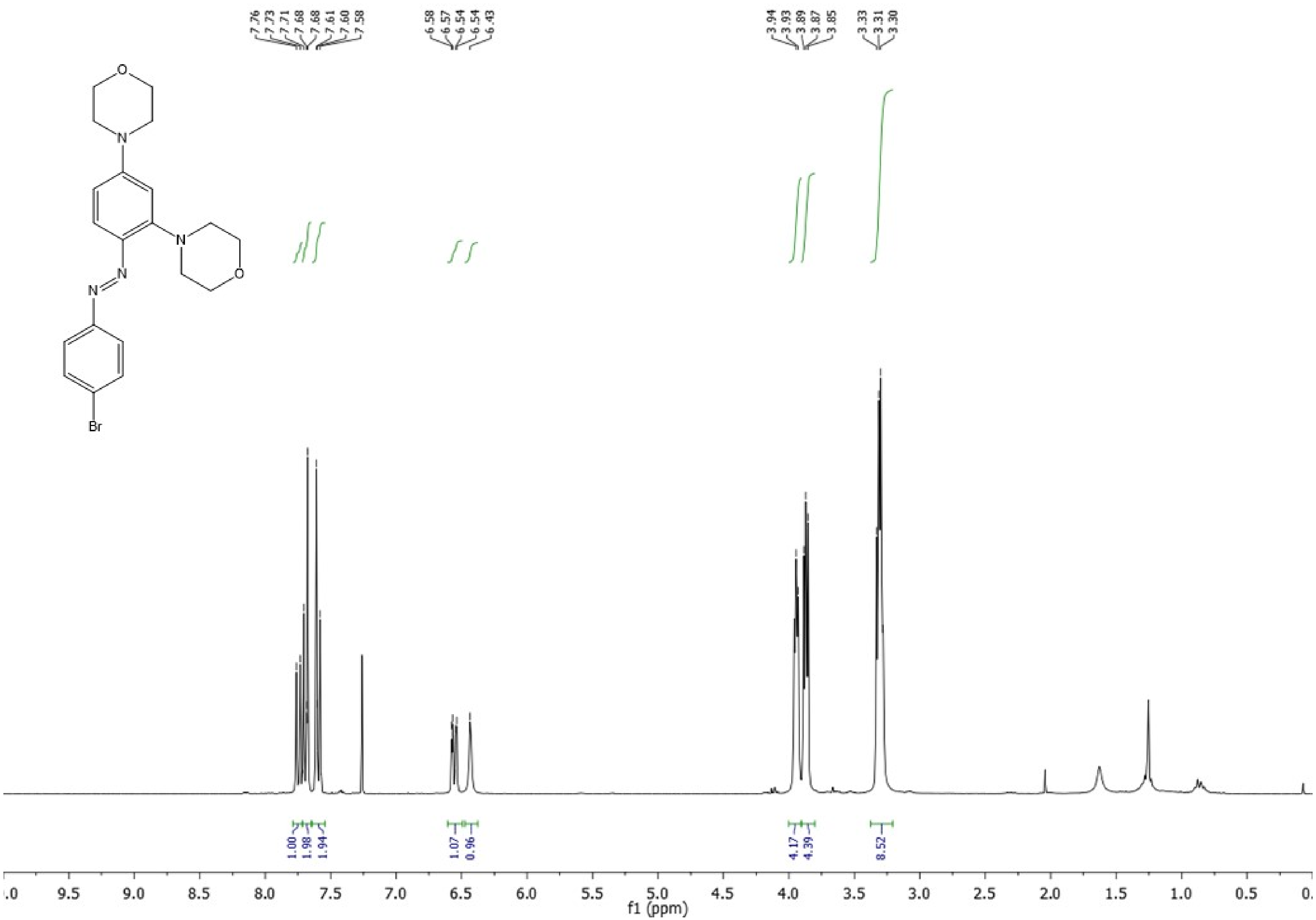

Fig. S23: ^{1}H-NMR spectrum of compound **7b** in $CDCl_3$.

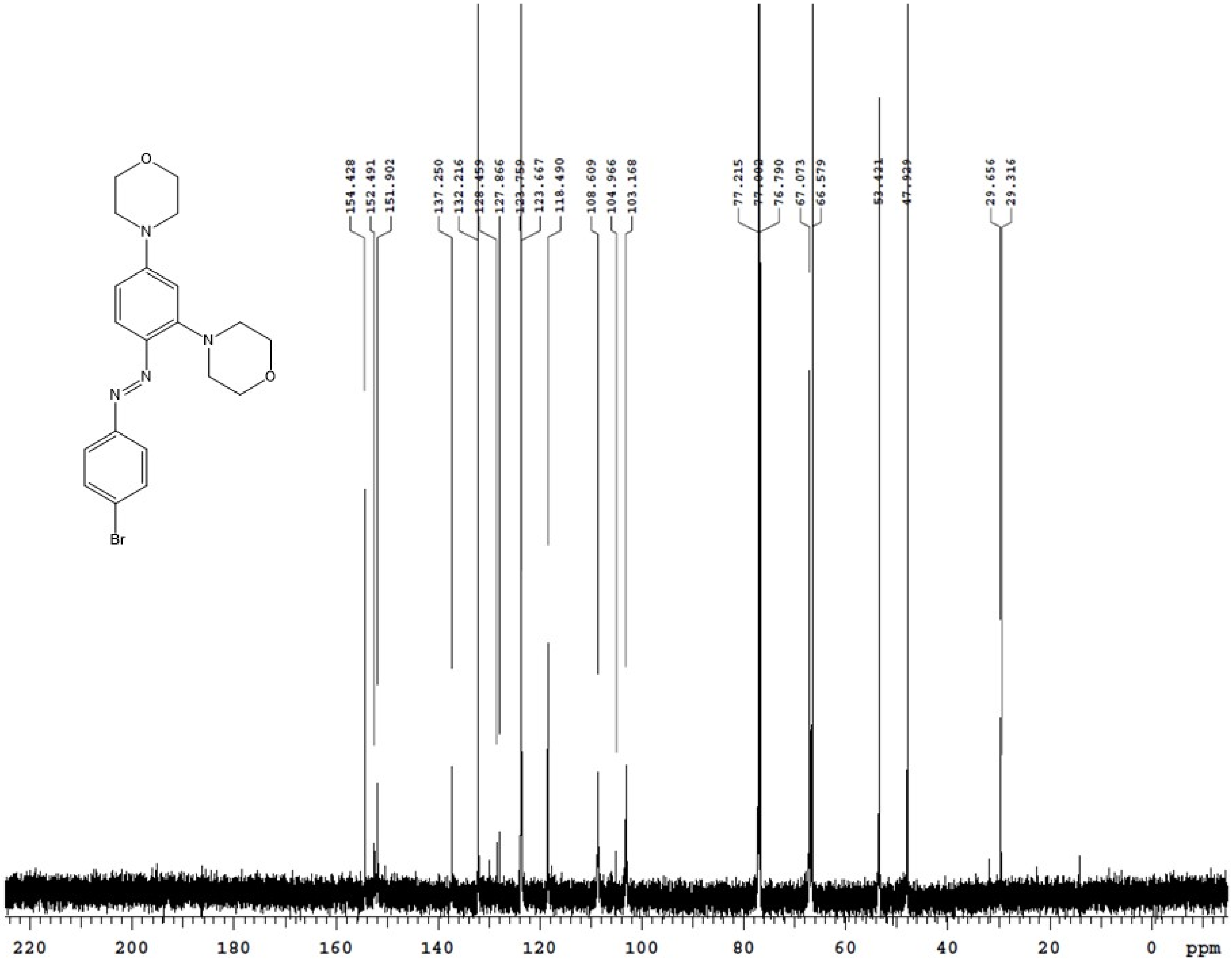

Fig. S24: ^{13}C-NMR spectrum of compound **7b** in $CDCl_3$.

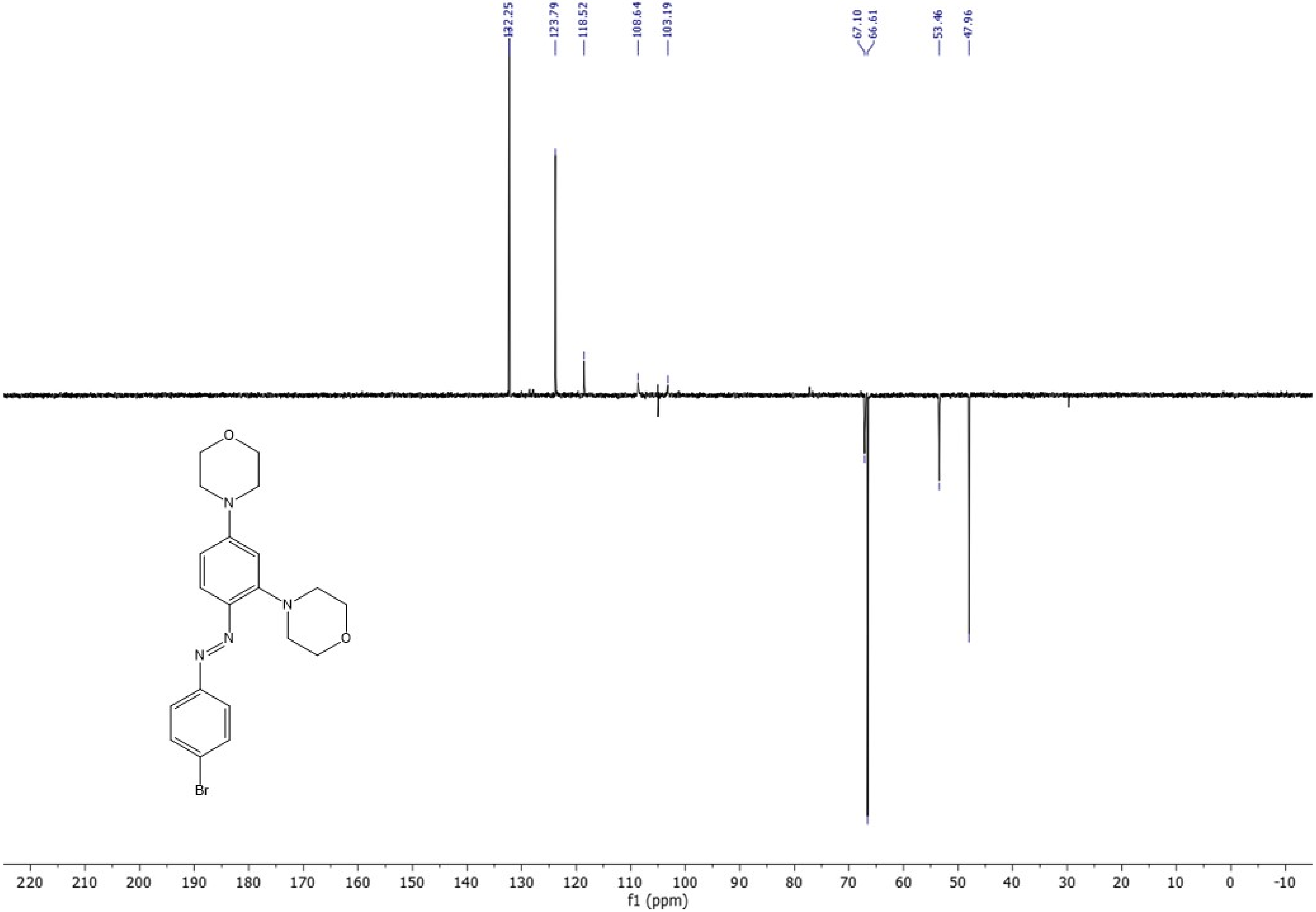

Fig. S25: DEPT spectrum of compound **7b** in $CDCl_3$.

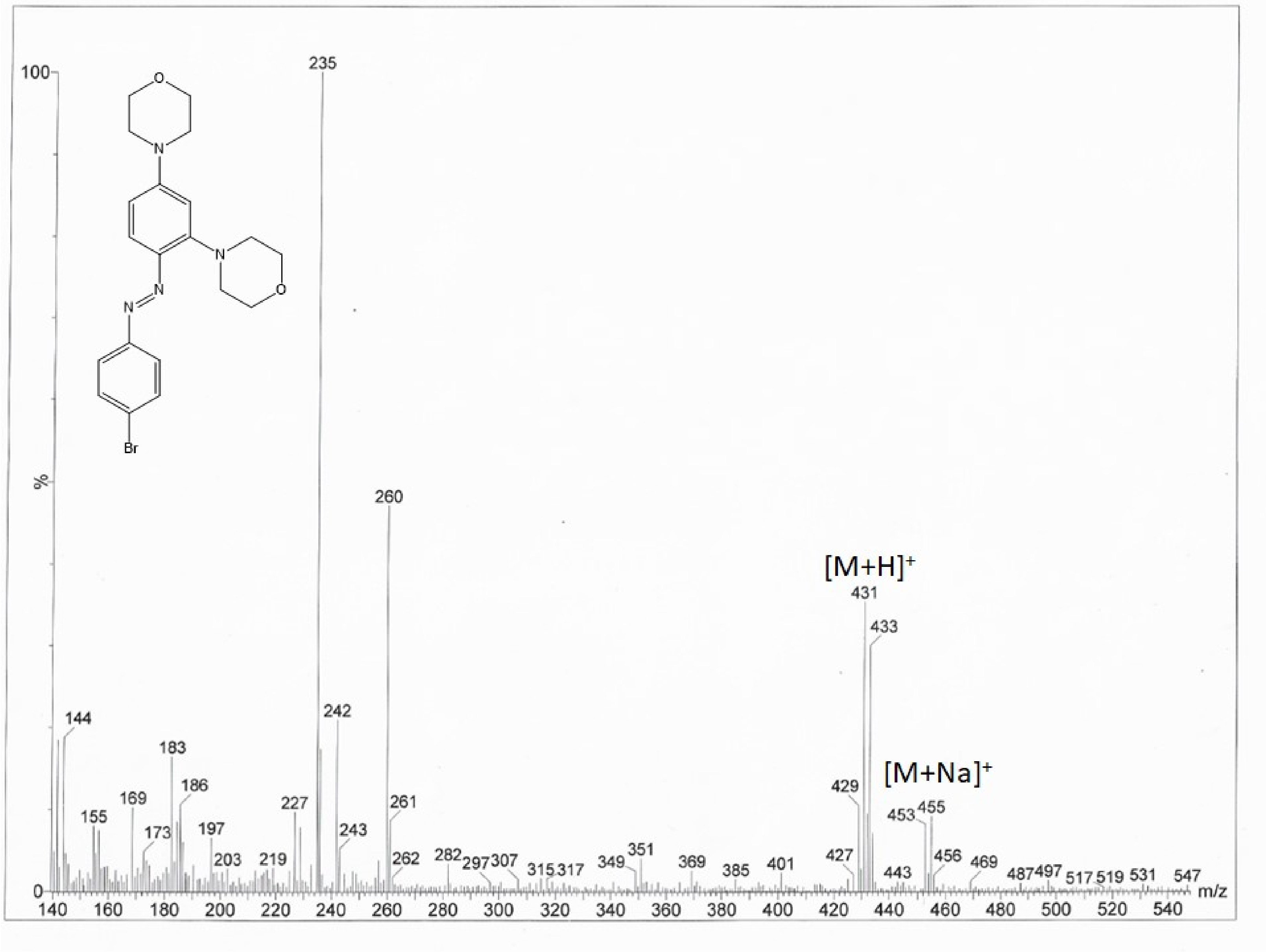

Fig. S26: ESI-MS+ (*m/*z) spectrum of compound **7b**.

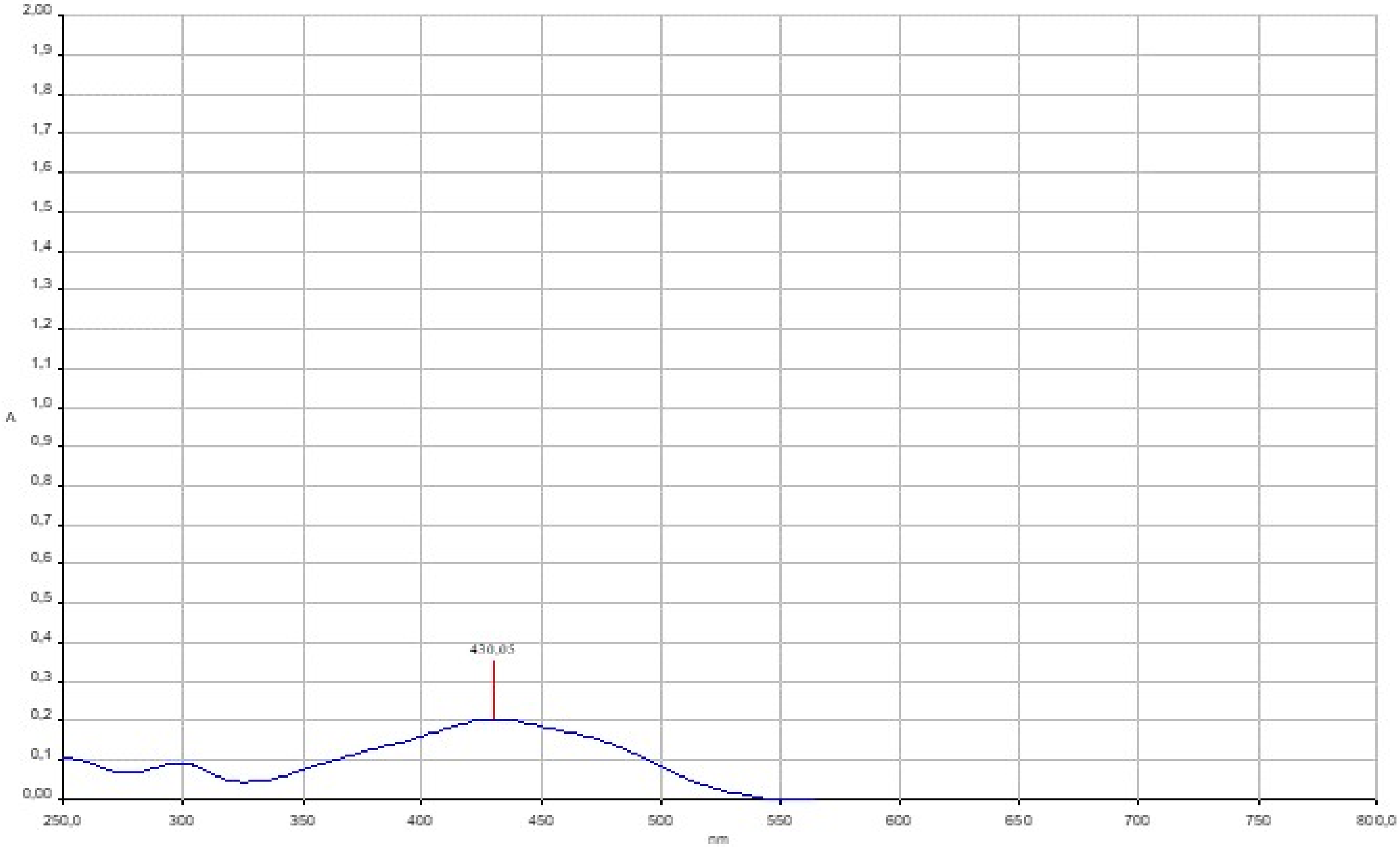

Fig. S27: UV-Vis spectrum of compound **7b** in $CHCl_3$.

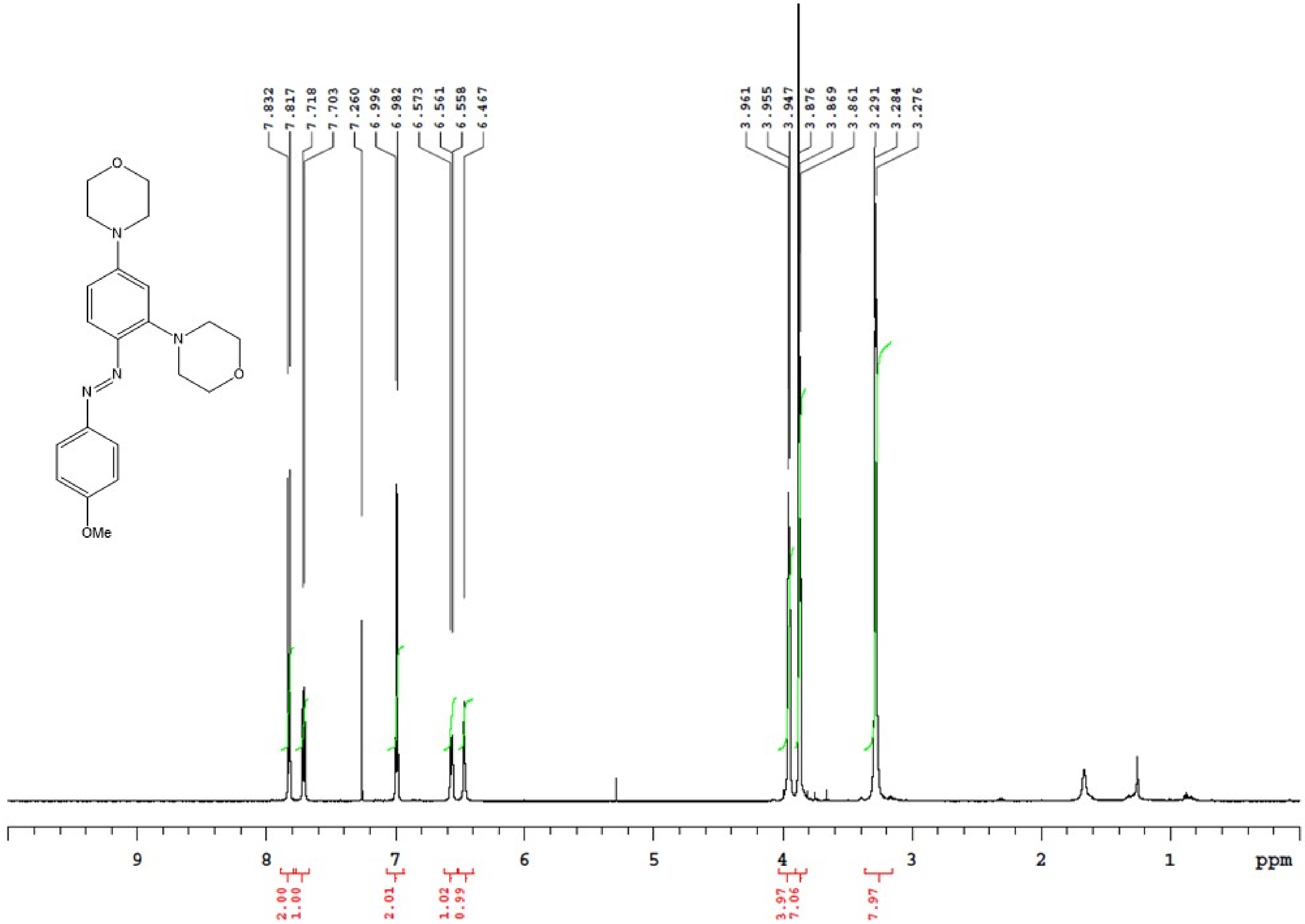

Fig. S28: ^{1}H-NMR spectrum of compound **7c** in $CDCl_3$.

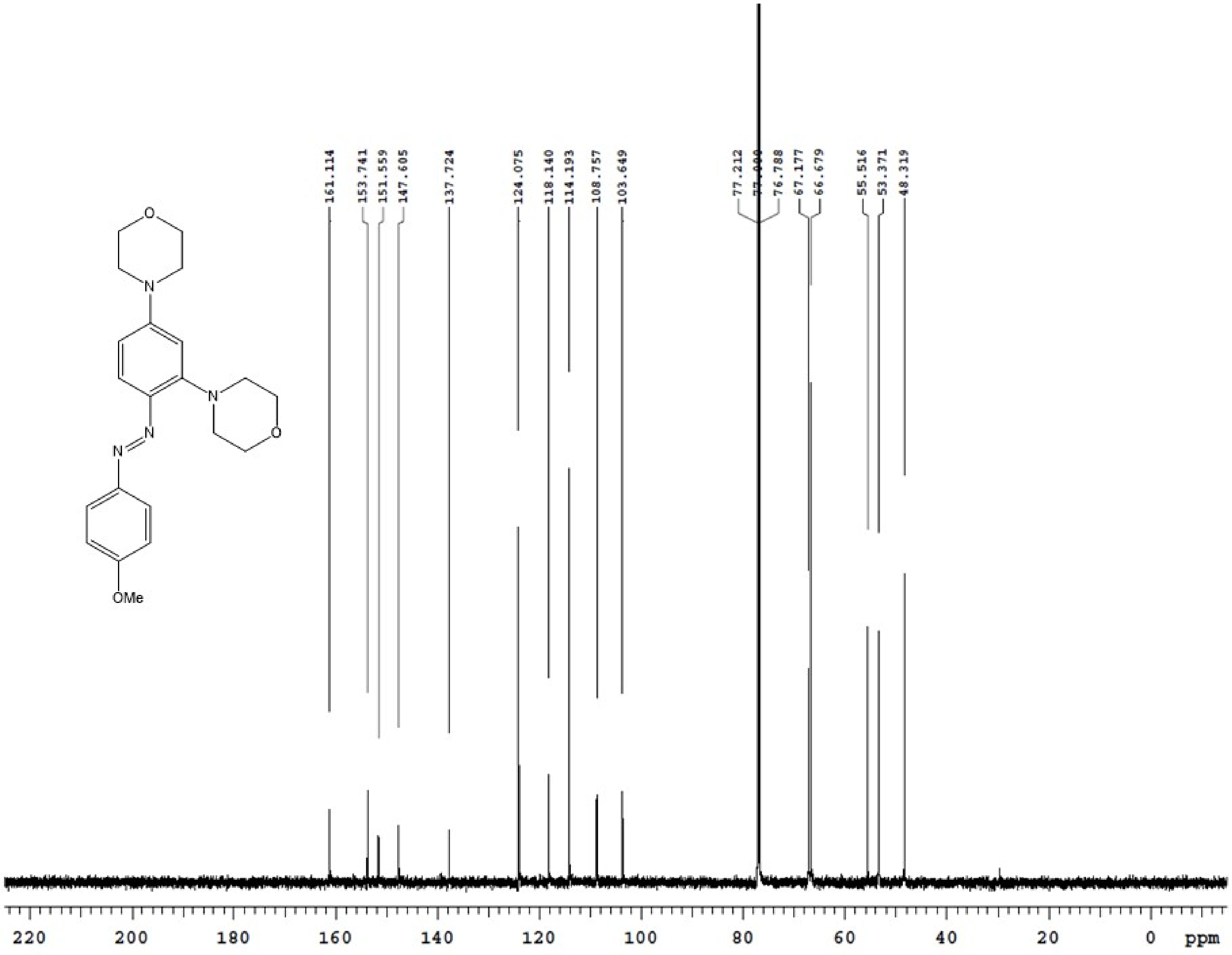

Fig. S29: ^{13}C-NMR spectrum of compound **7c** in $CDCl_3$.

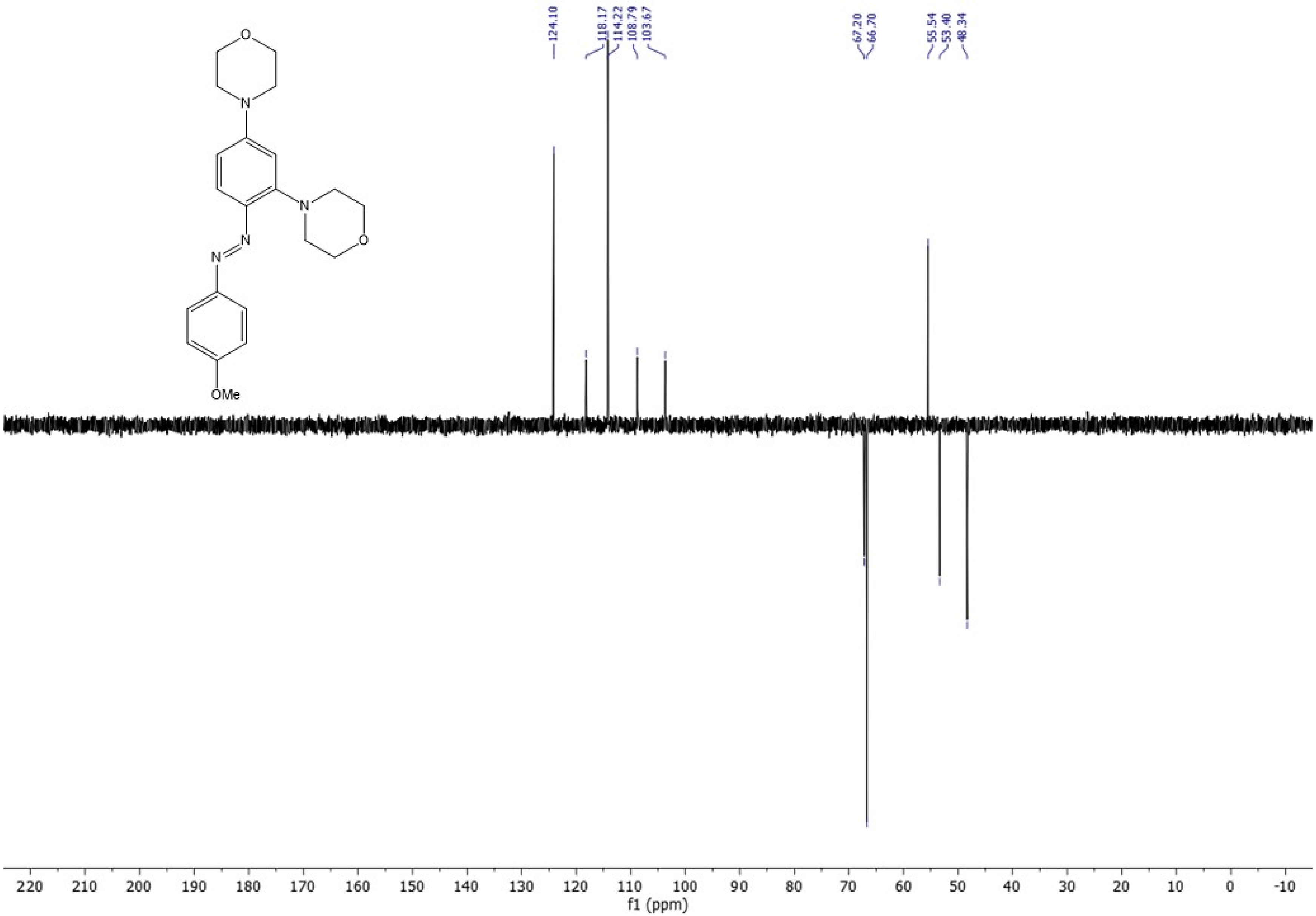

Fig. S30: DEPT spectrum of compound **7c** in $CDCl_3$.

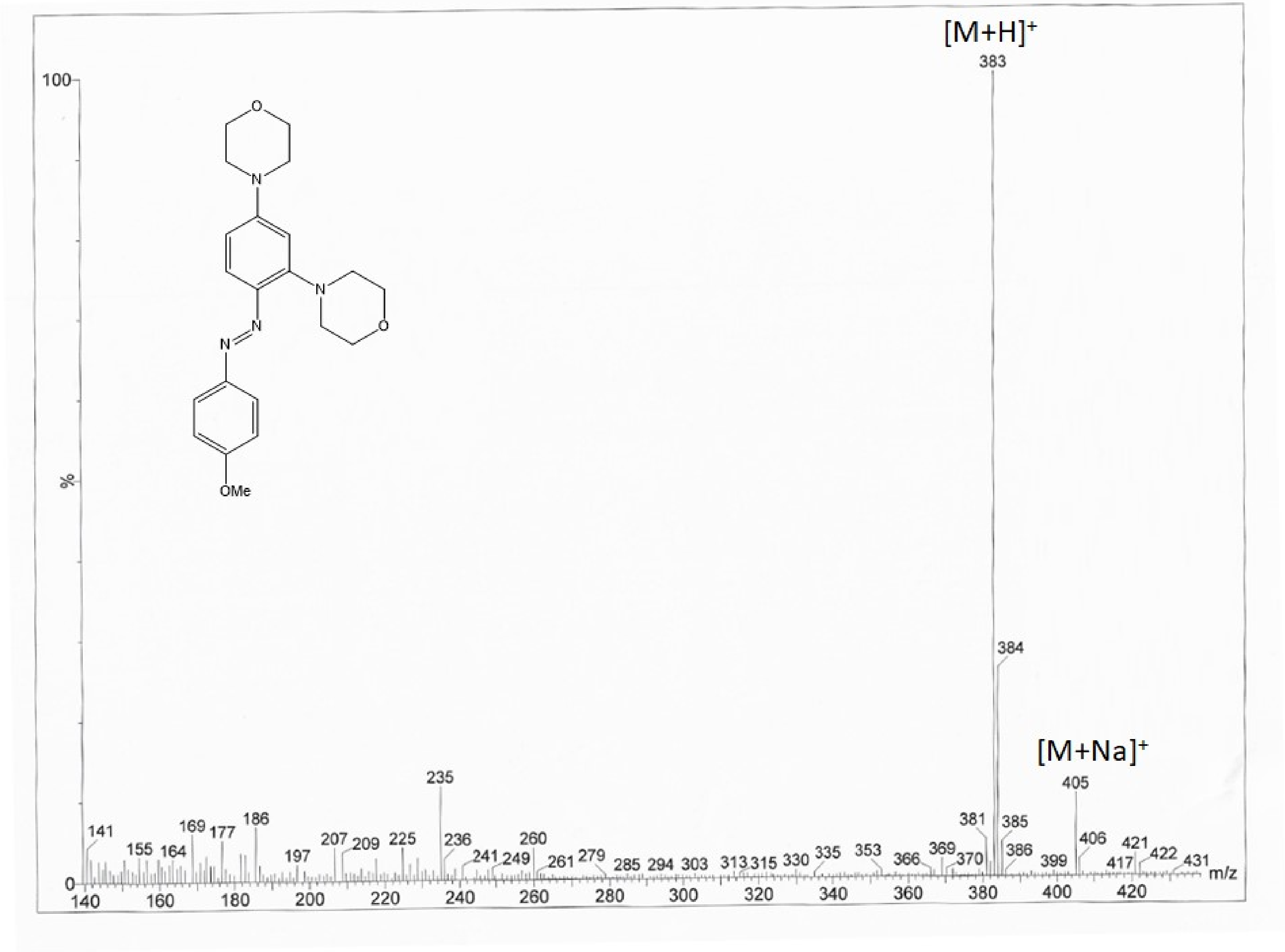

Fig. S31: ESI-MS+ (*m/*z) spectrum of compound **7c**.

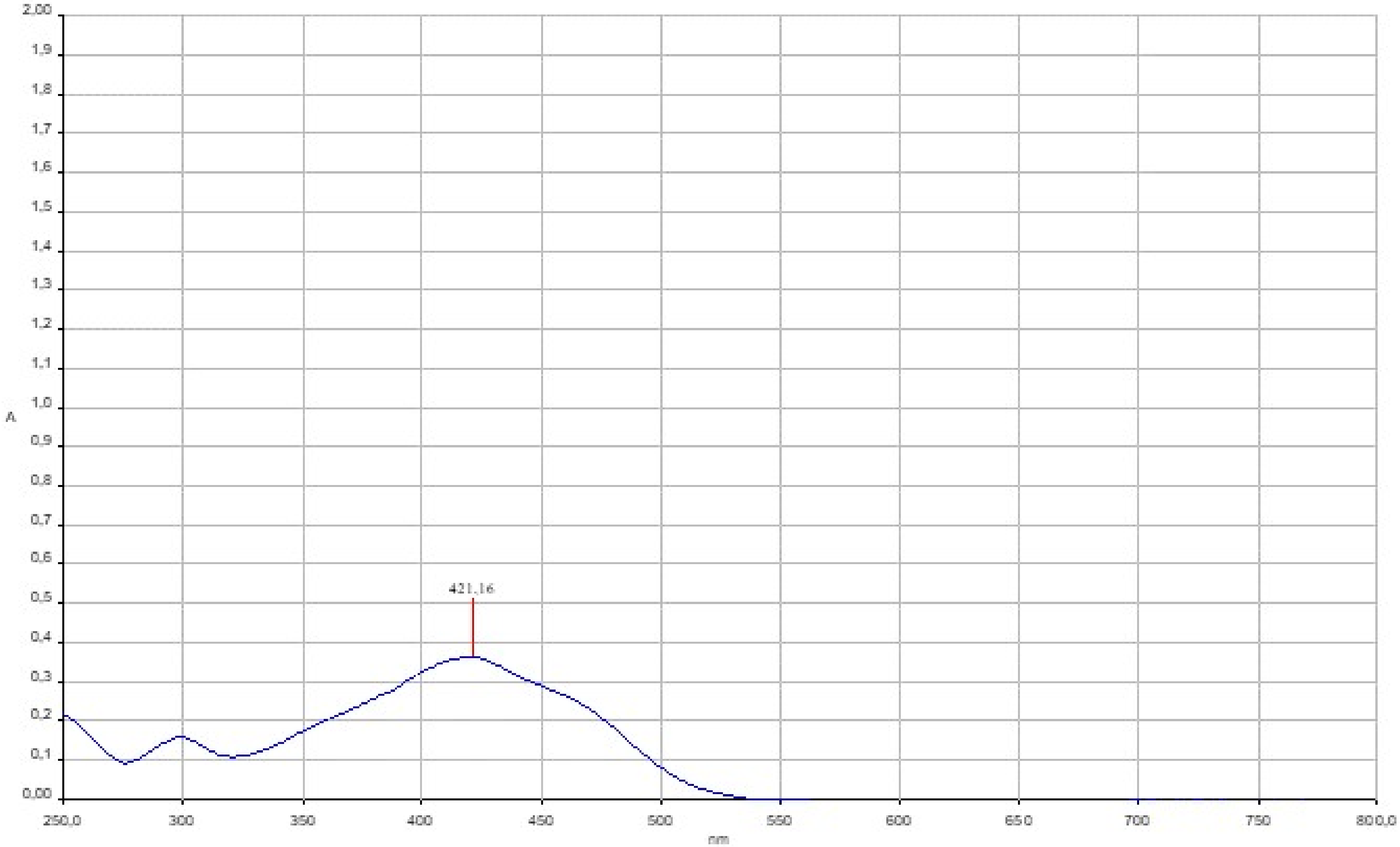

Fig. S32: UV-Vis spectrum of compound **7c** in $CHCl_3$.

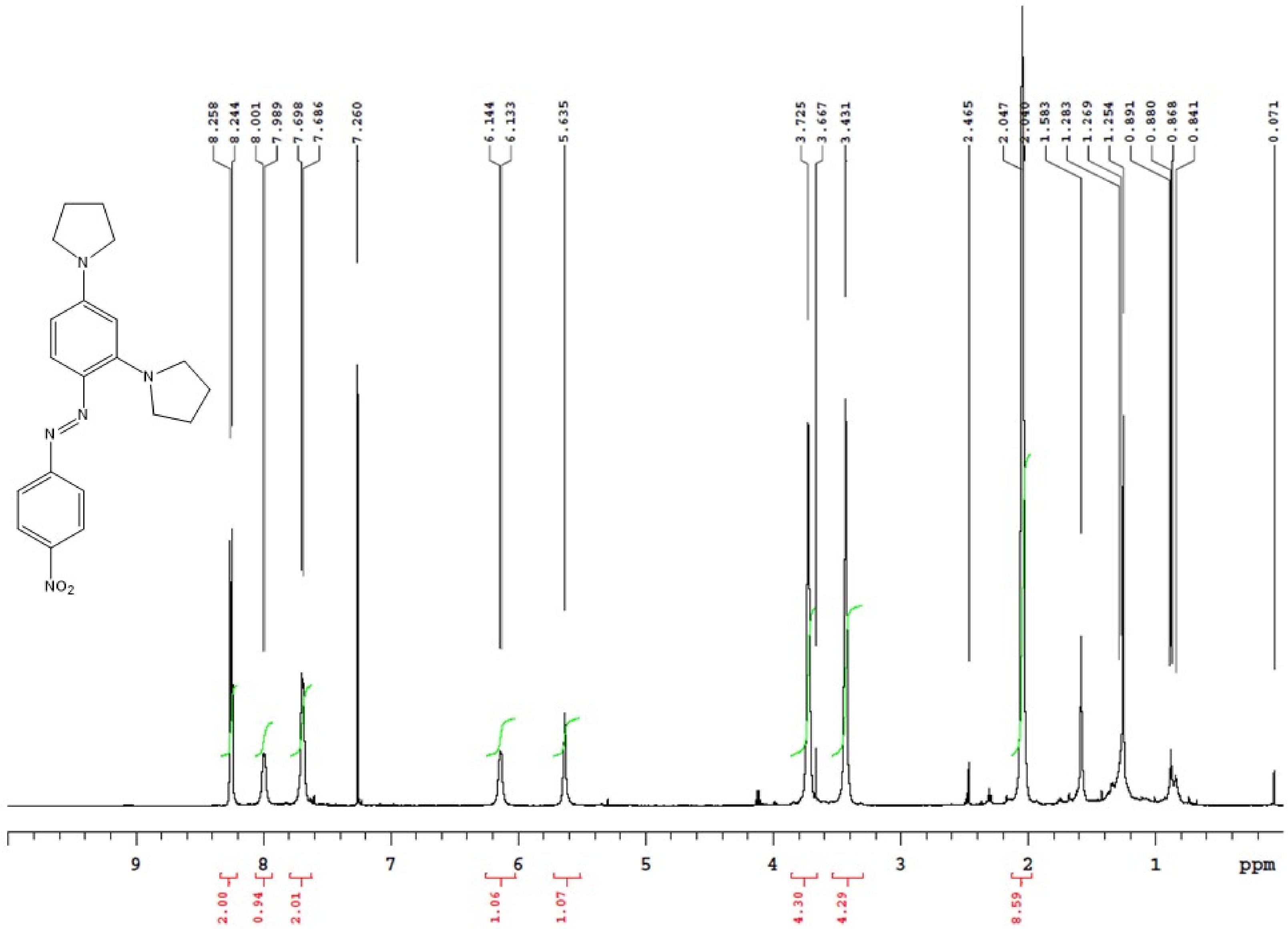

Fig. S33: ¹H-NMR spectrum of compound **8a** in $CDCl_3$.

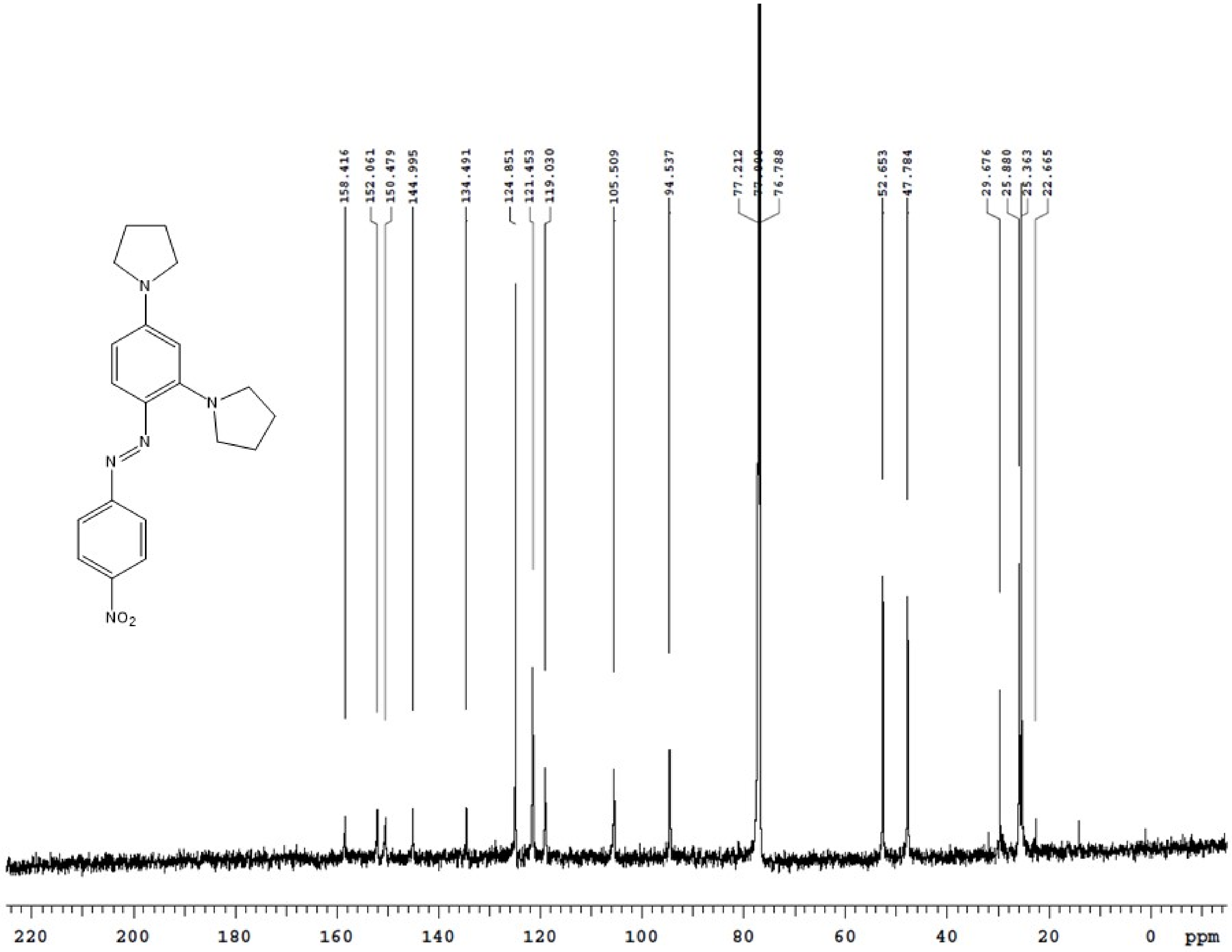

Fig. S34: ^{13}C-NMR spectrum of compound **8a** in $CDCl_3$.

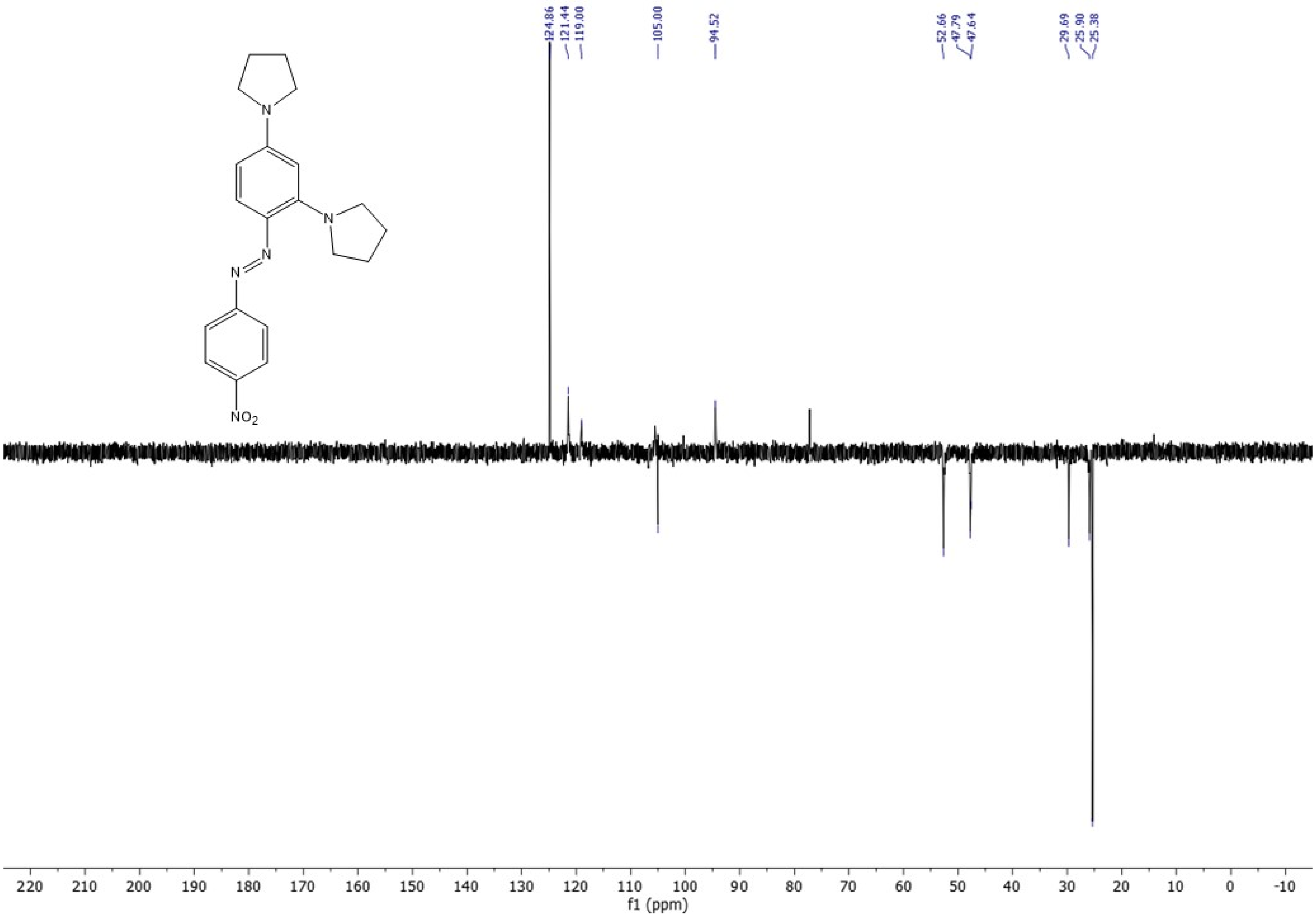

Fig. S35: DEPT spectrum of compound **8a** in $CDCl_3$.

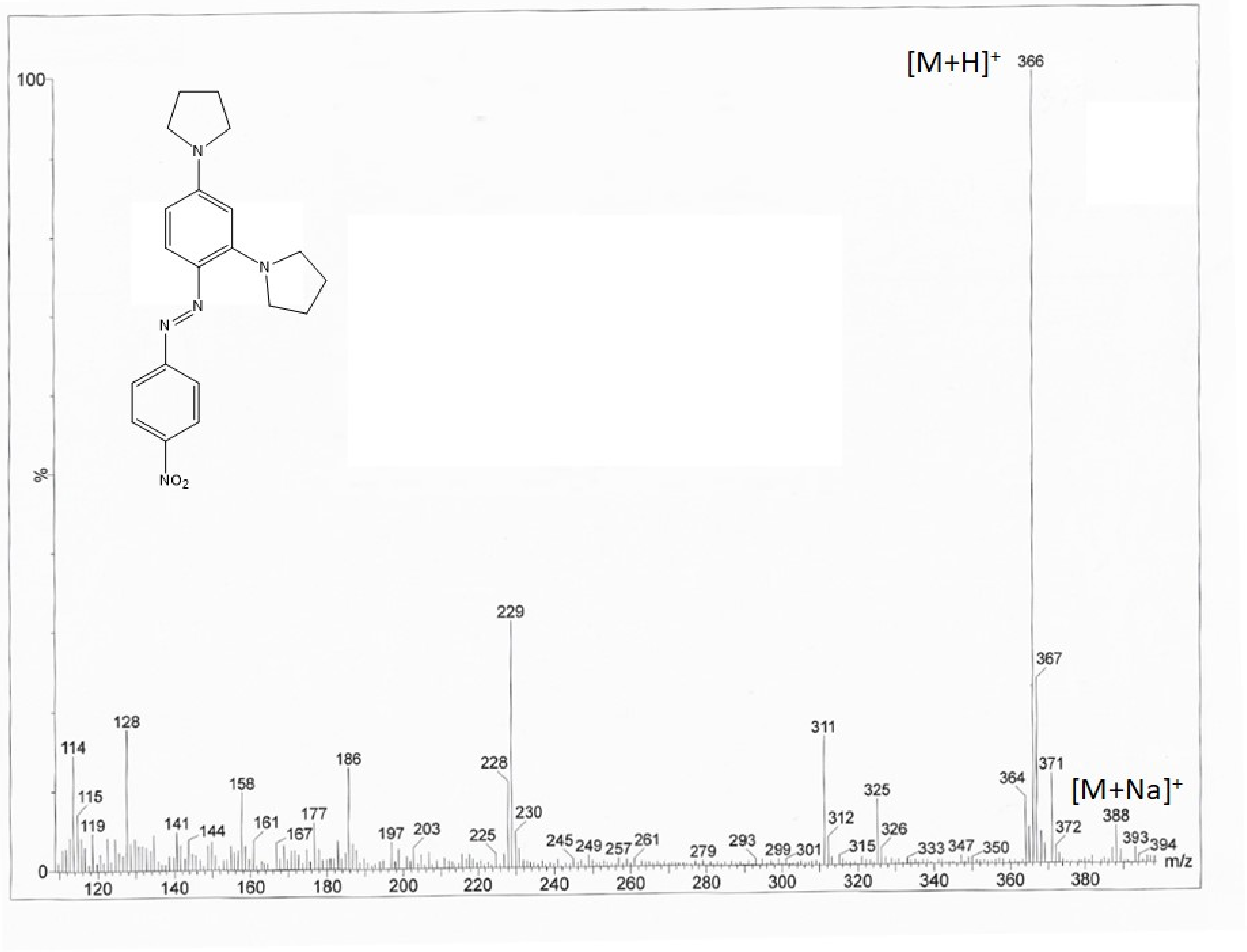

Fig. S36: ESI-MS+ (*m/z*) spectrum of compound **8a**.

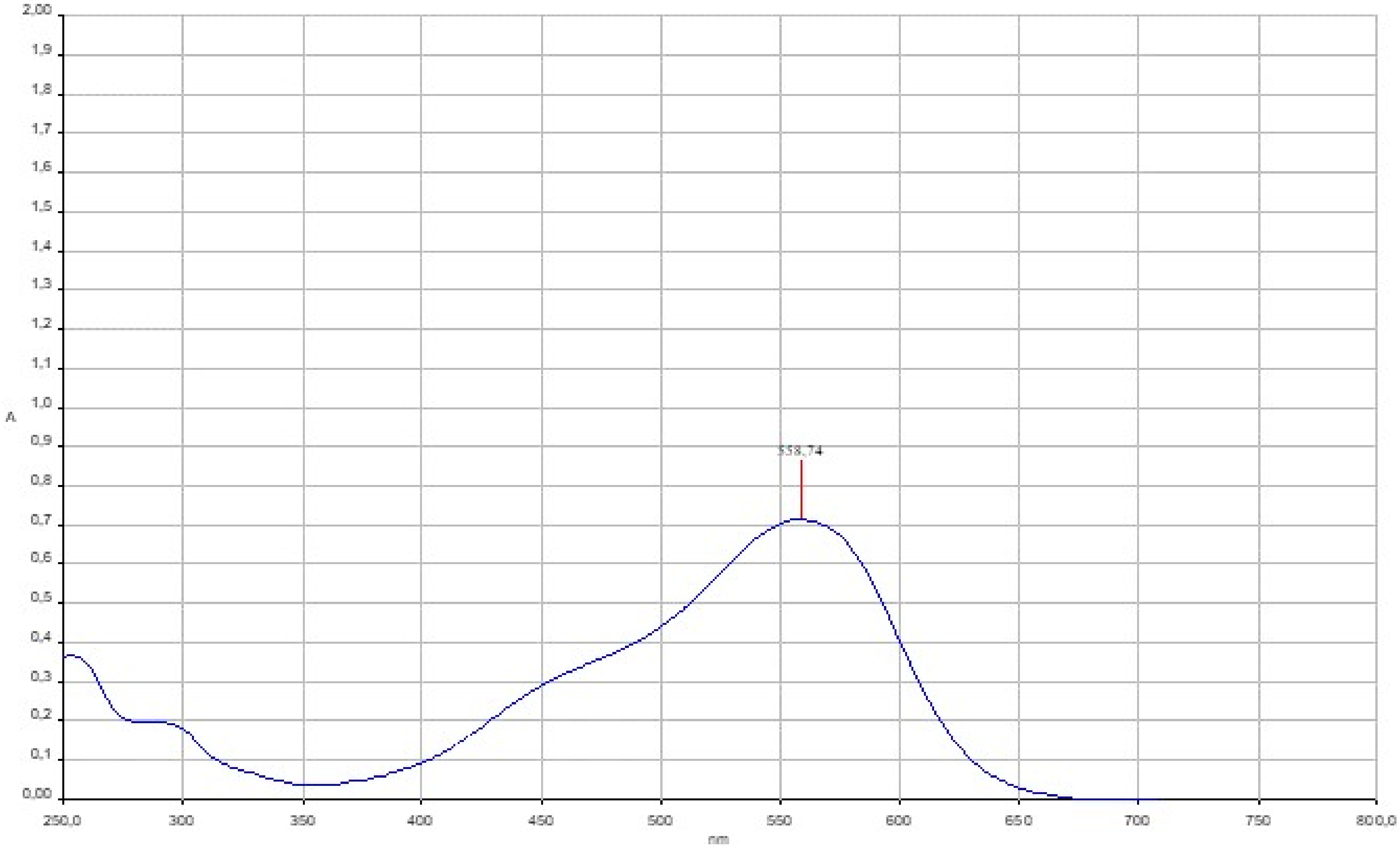

Fig. S37: UV-Vis spectrum of compound **9a** in $CHCl_3$.

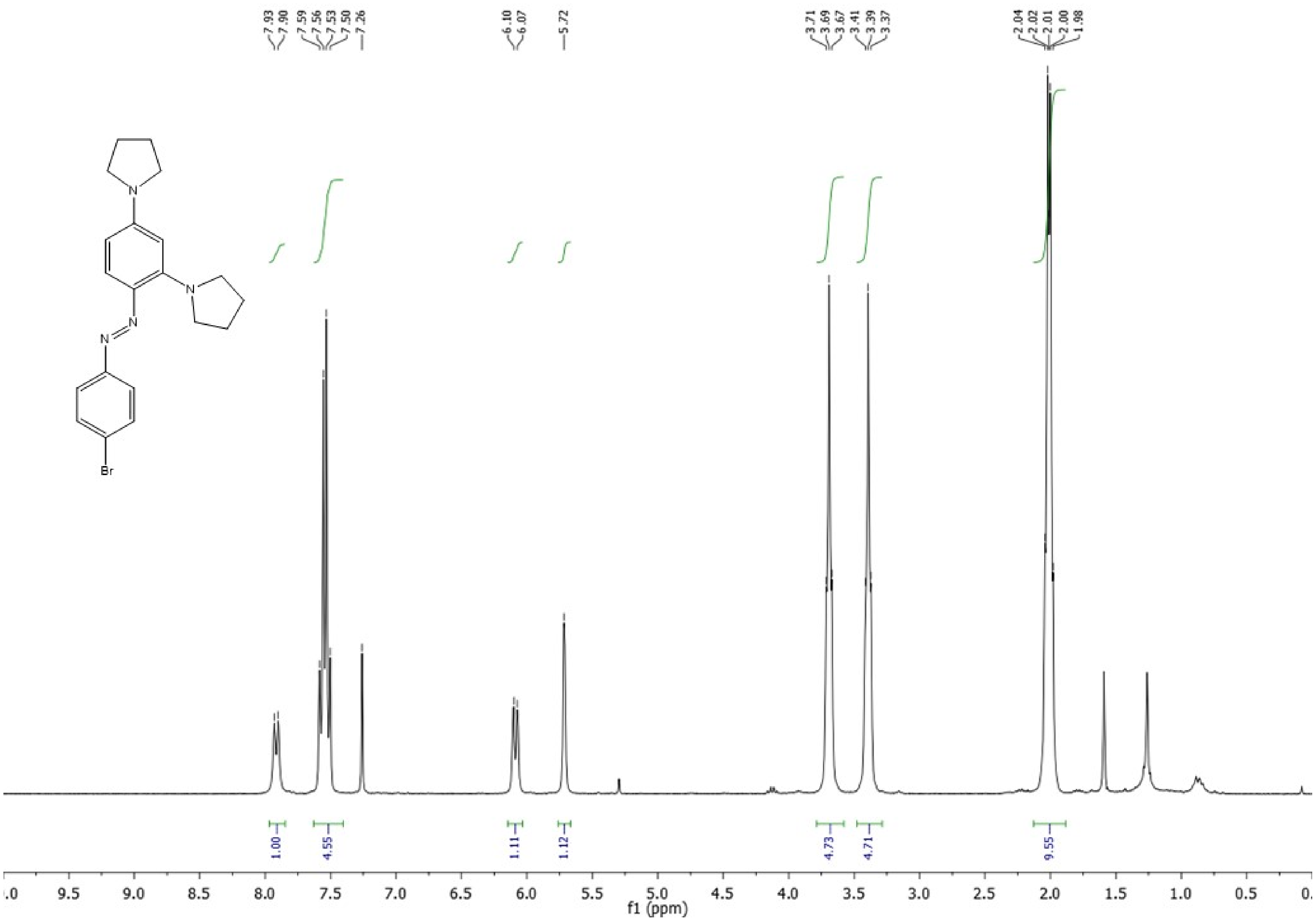

Fig. S38: ^{1}H-NMR spectrum of compound **8b** in $CDCl_3$.

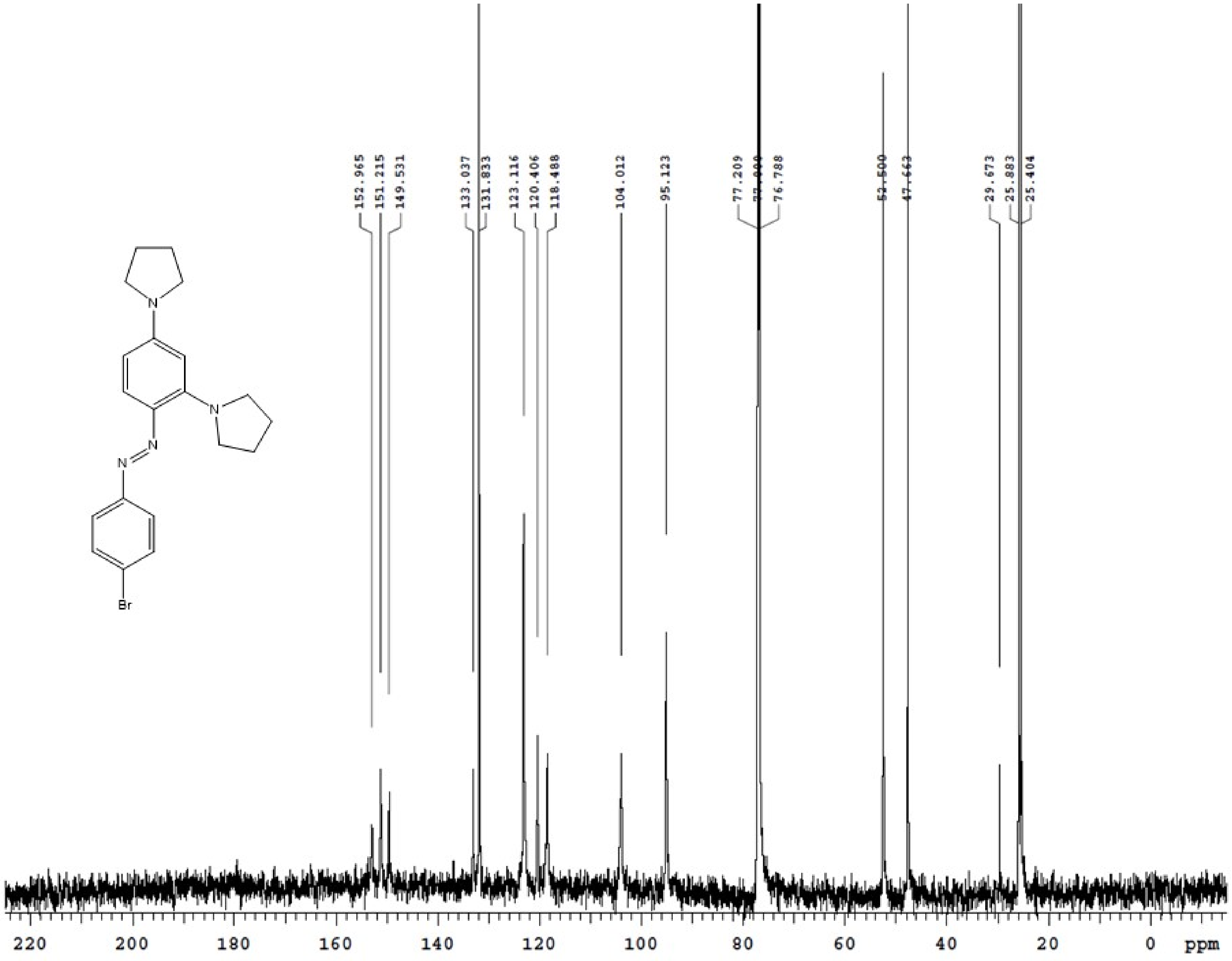

Fig. S39: ^{13}C-NMR spectrum of compound **8b** in $CDCl_3$.

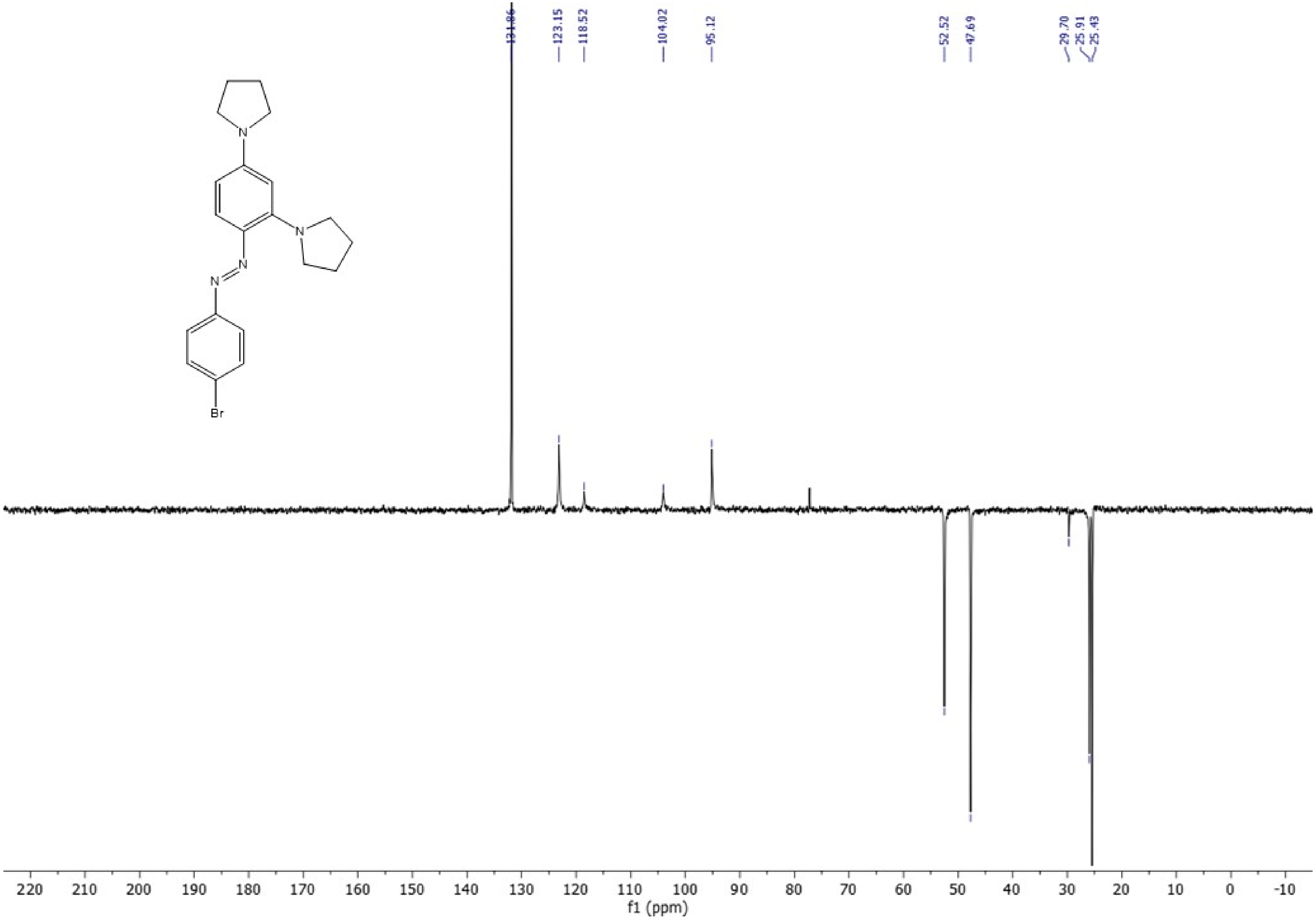

Fig. S40: DEPT spectrum of compound **8b** in $CDCl_3$.

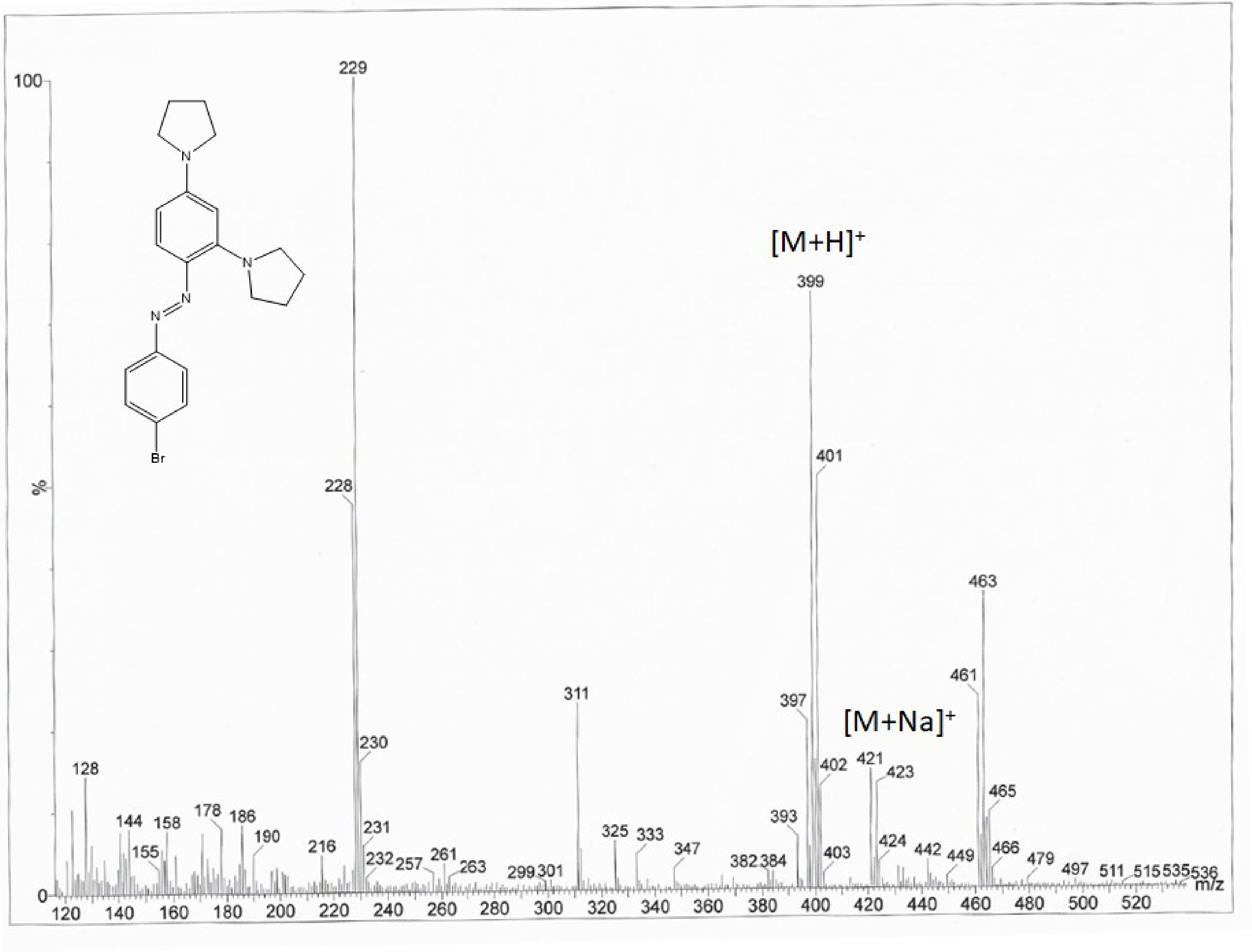

Fig. S41: ESI-MS+ (*m/*z) spectrum of compound **8b**.

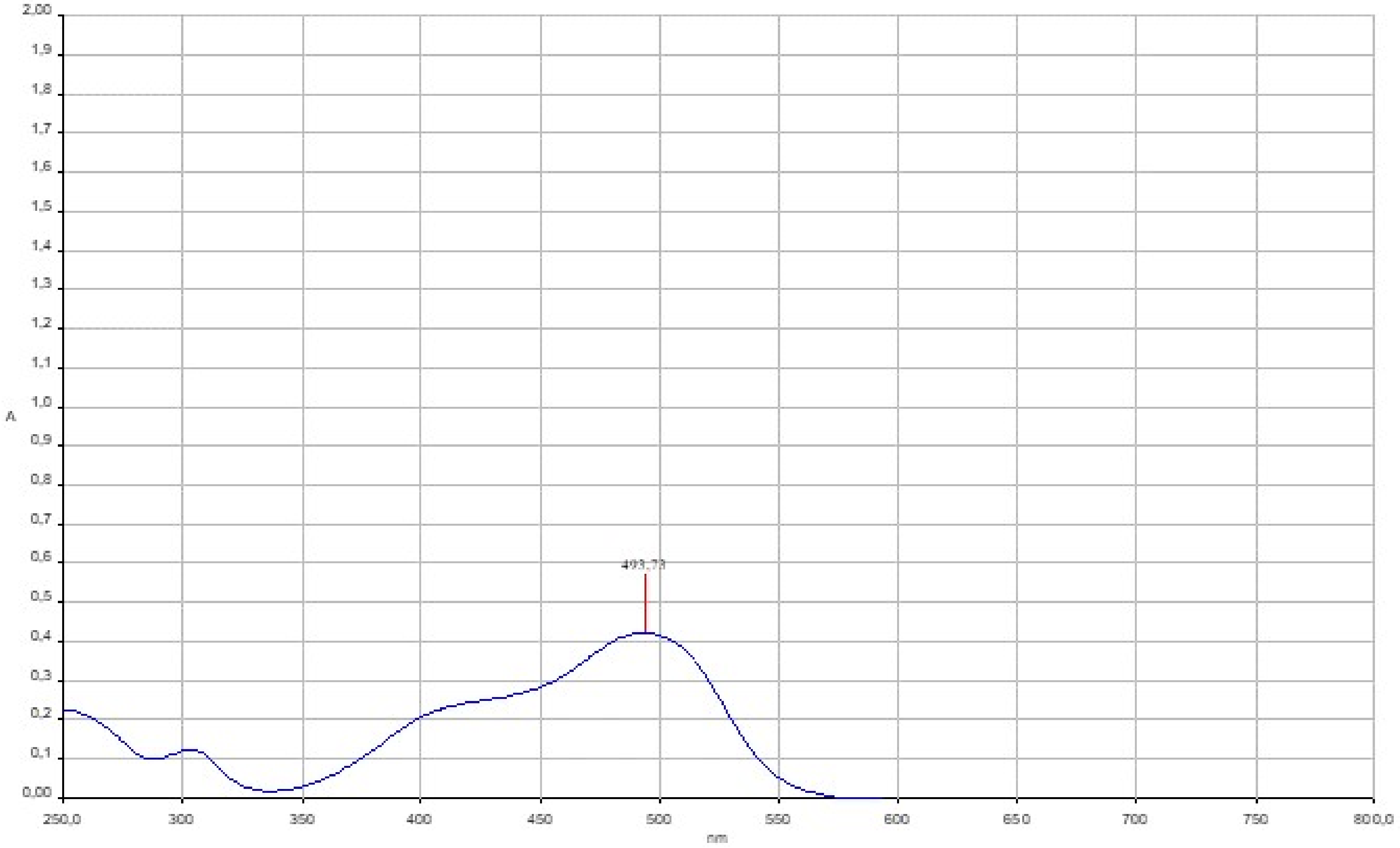

Fig. S42: UV-Vis spectrum of compound **8b** in $CHCl_3$.

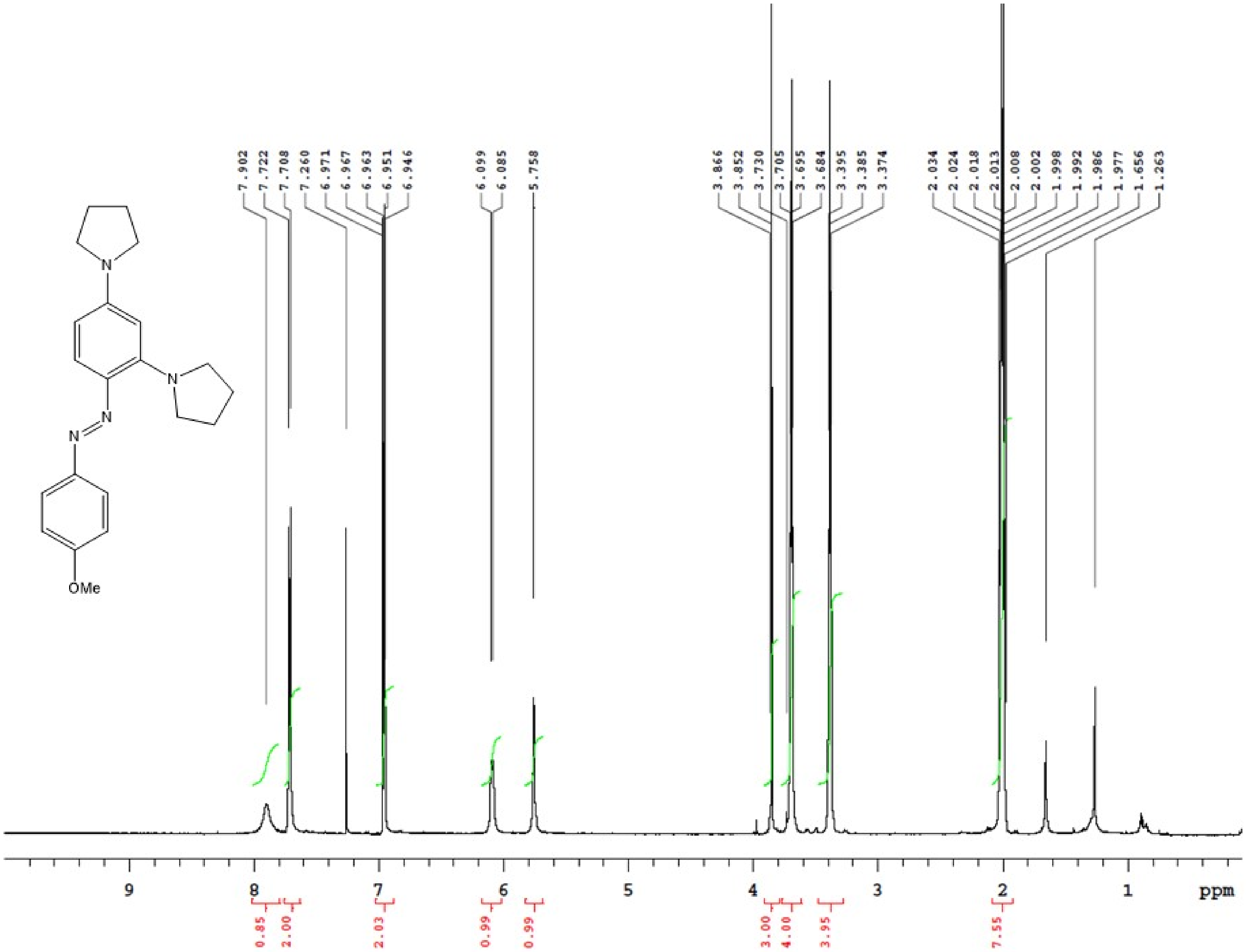

Fig. S43: ^{1}H-NMR spectrum of compound **8c** in $CDCl_3$.

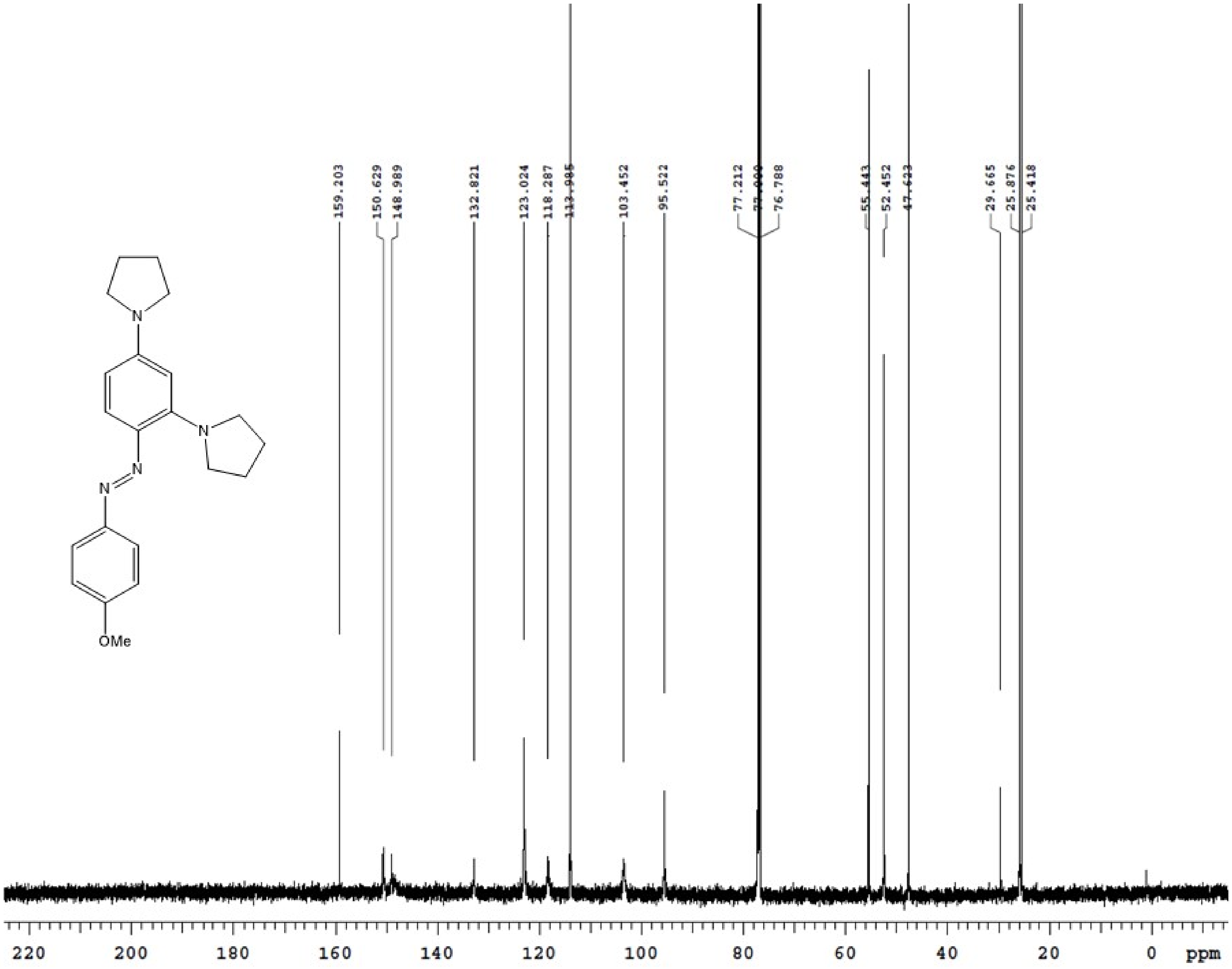

Fig. S44: ^{13}C-NMR spectrum of compound **8c** in $CDCl_3$.

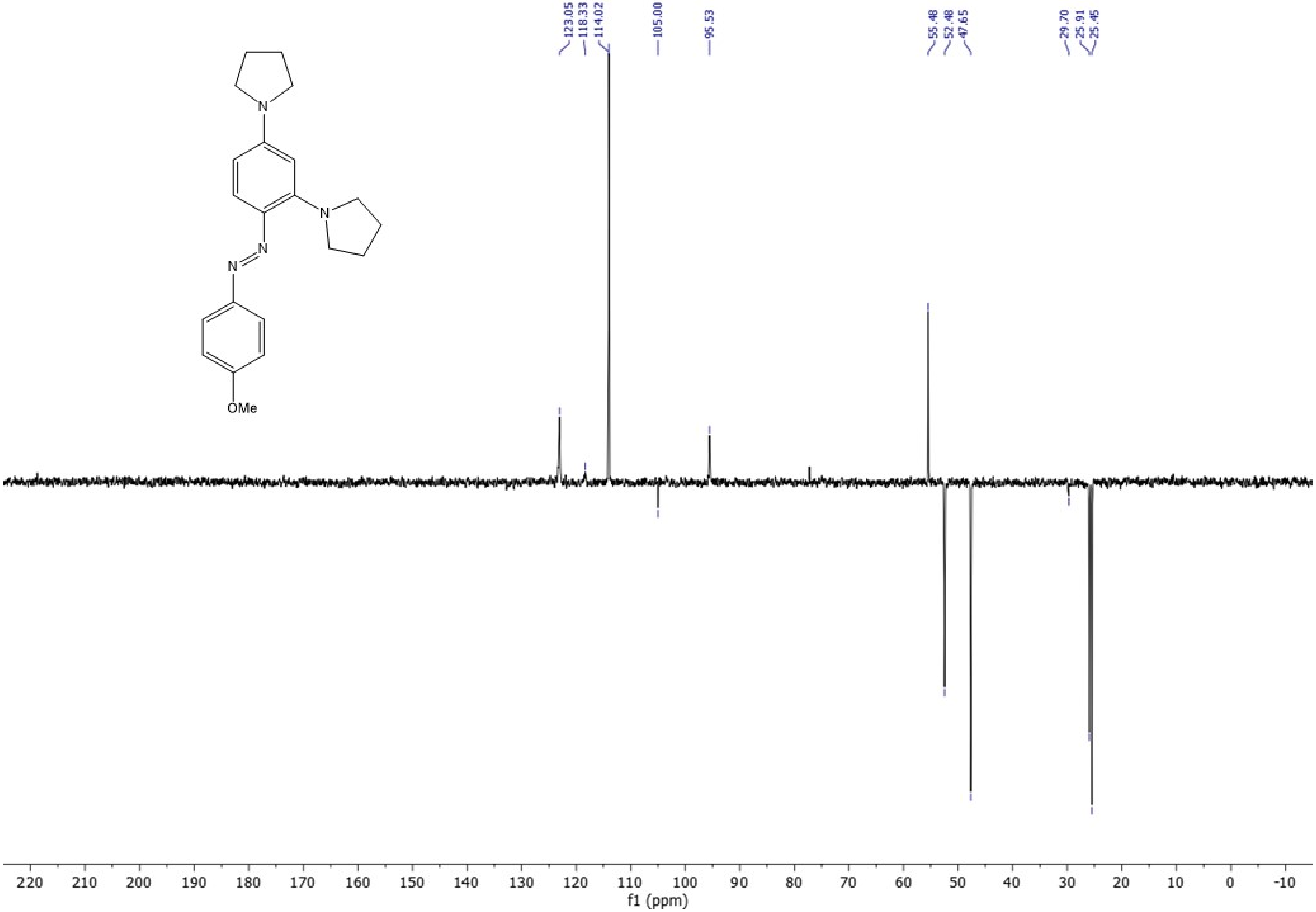

Fig. S45: DEPT spectrum of compound **8c** in $CDCl_3$.

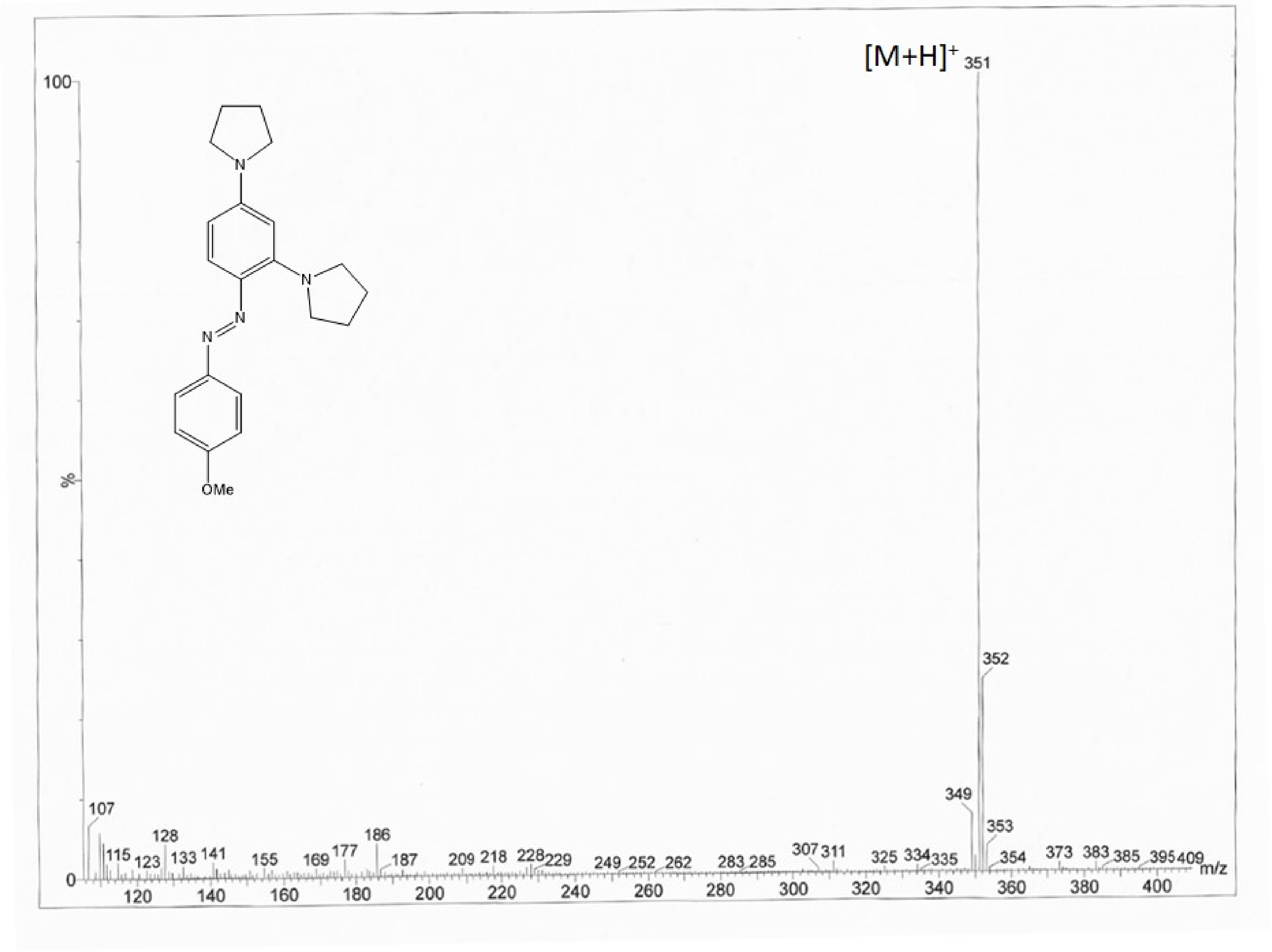

Fig. S46: ESI-MS+ (*m/z*) spectrum of compound **8c**.

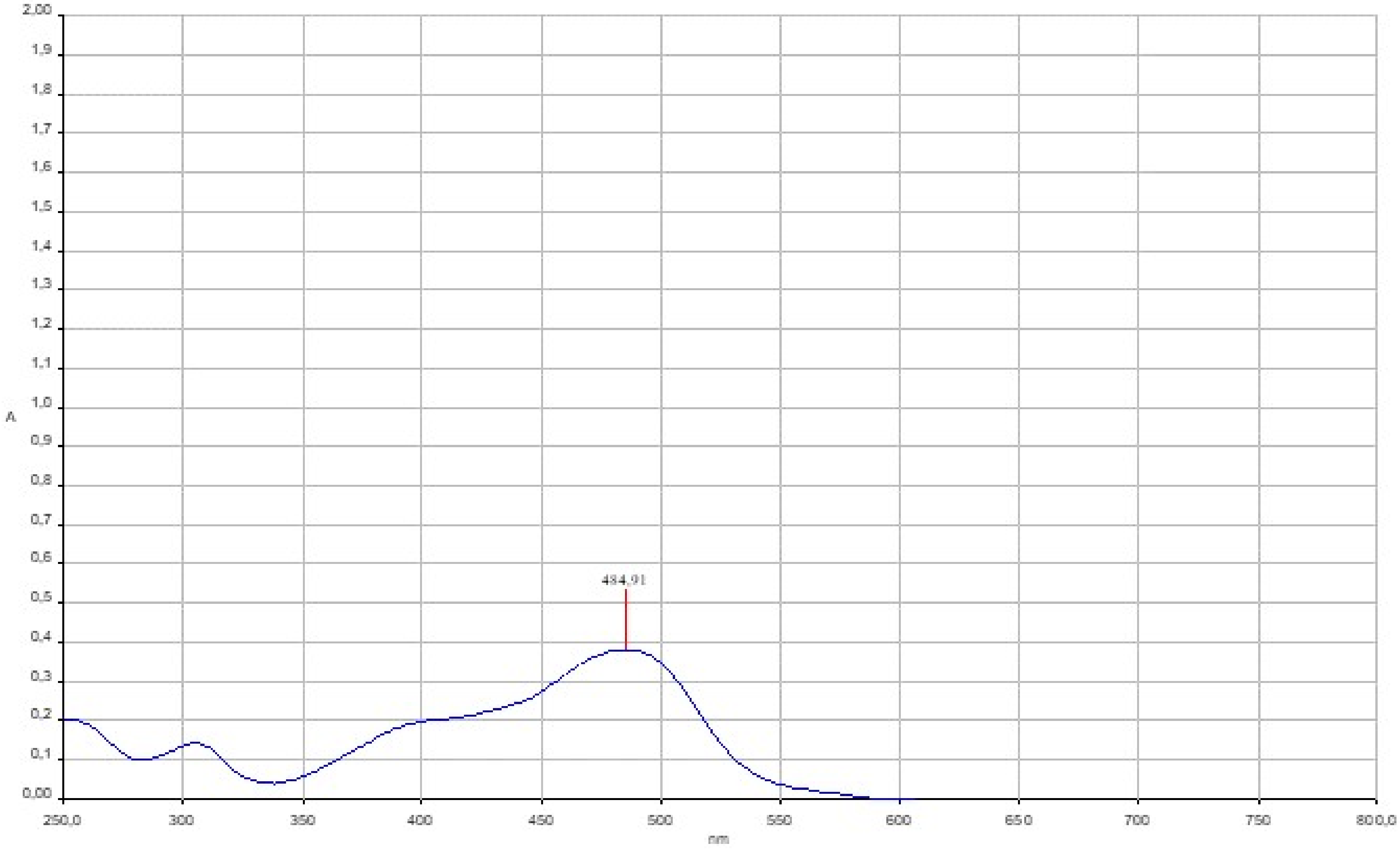

Fig. S47: UV-Vis spectrum of compound **8c** in $CHCl_3$.

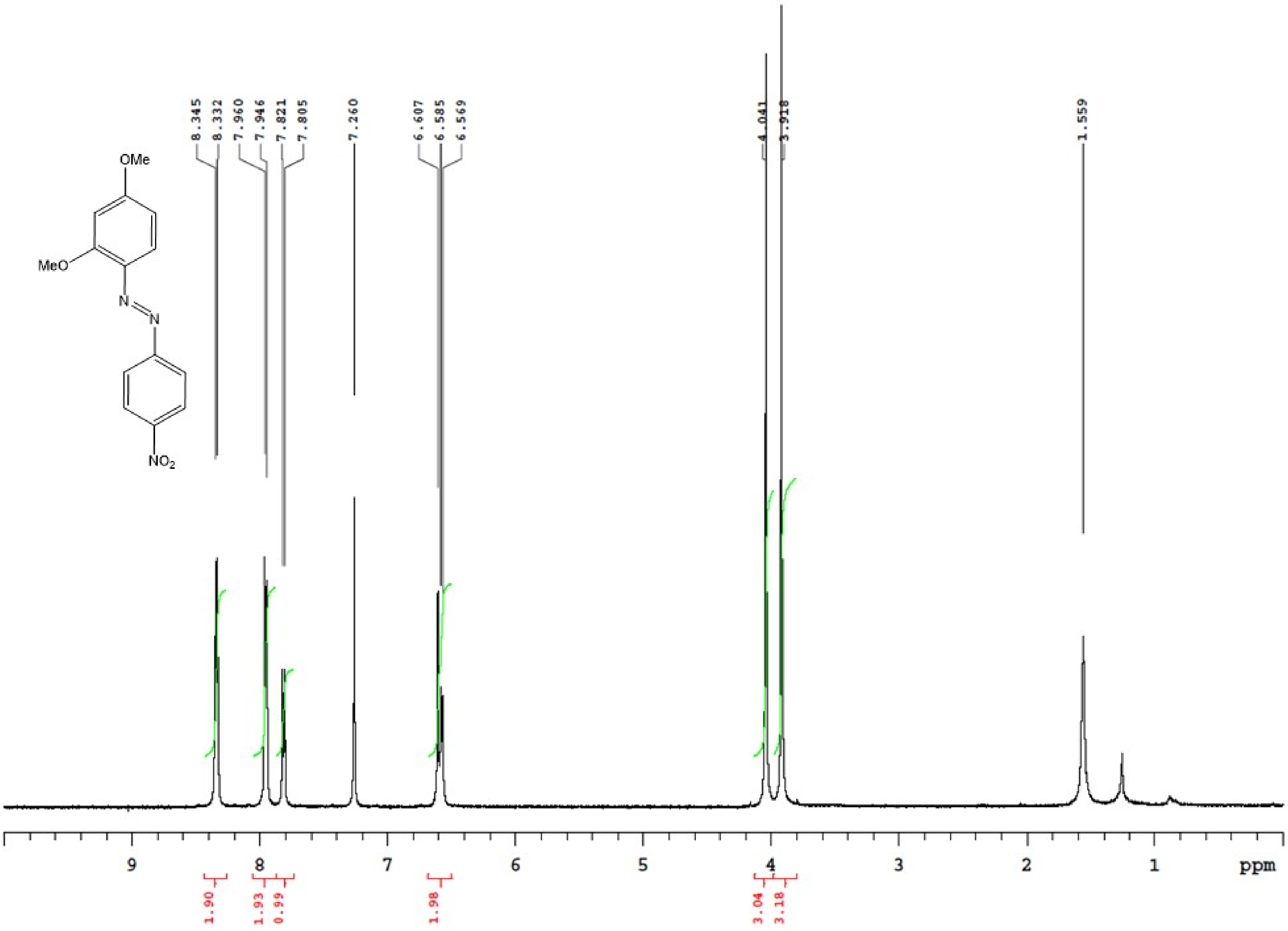

Fig. S48: ^{1}H-NMR spectrum of compound **9a** in $CDCl_3$.

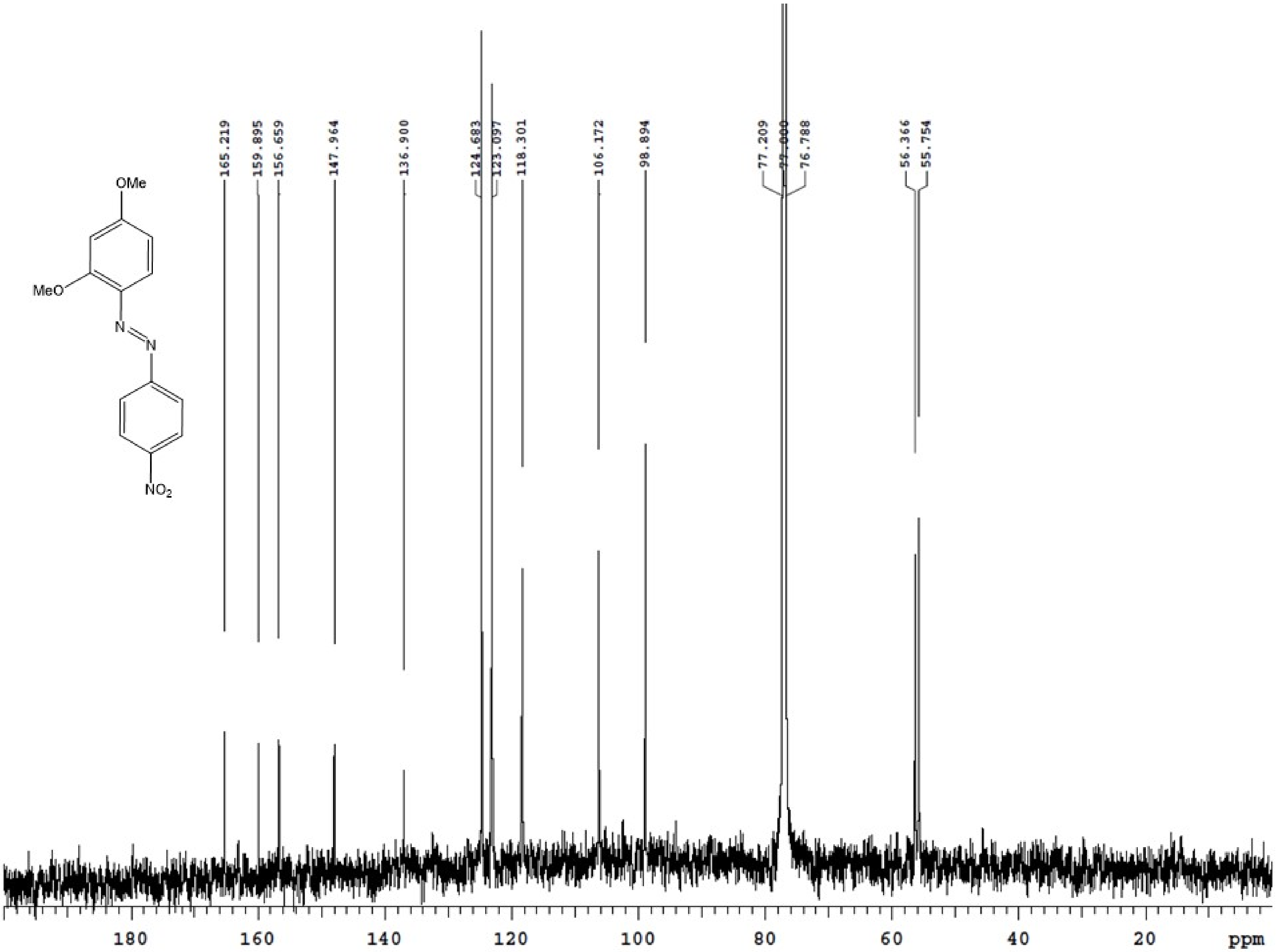

Fig. S49: ^{13}C-NMR spectrum of compound **9a** in $CDCl_3$.

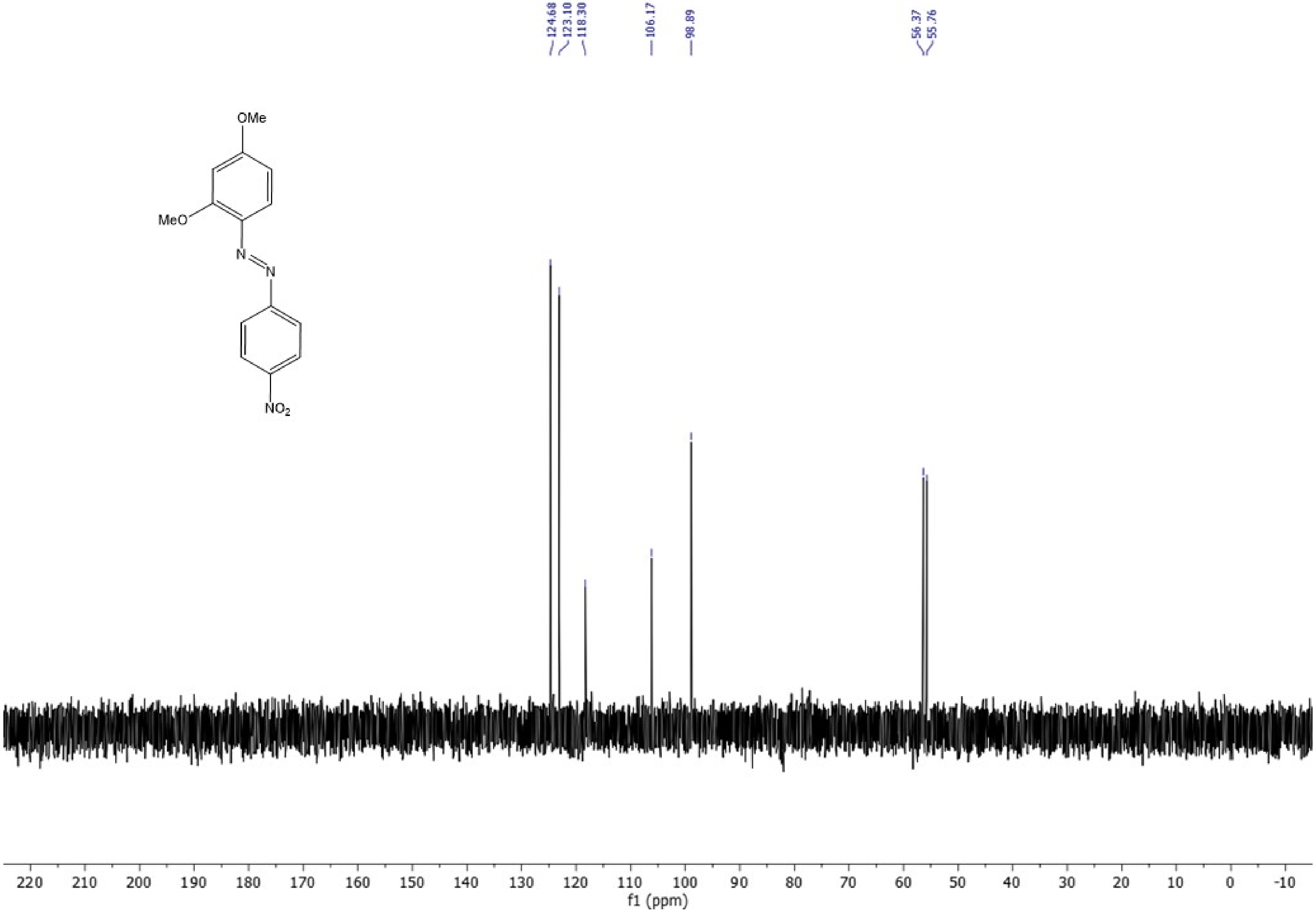

Fig. S50: DEPT spectrum of compound **9a** in $CDCl_3$.

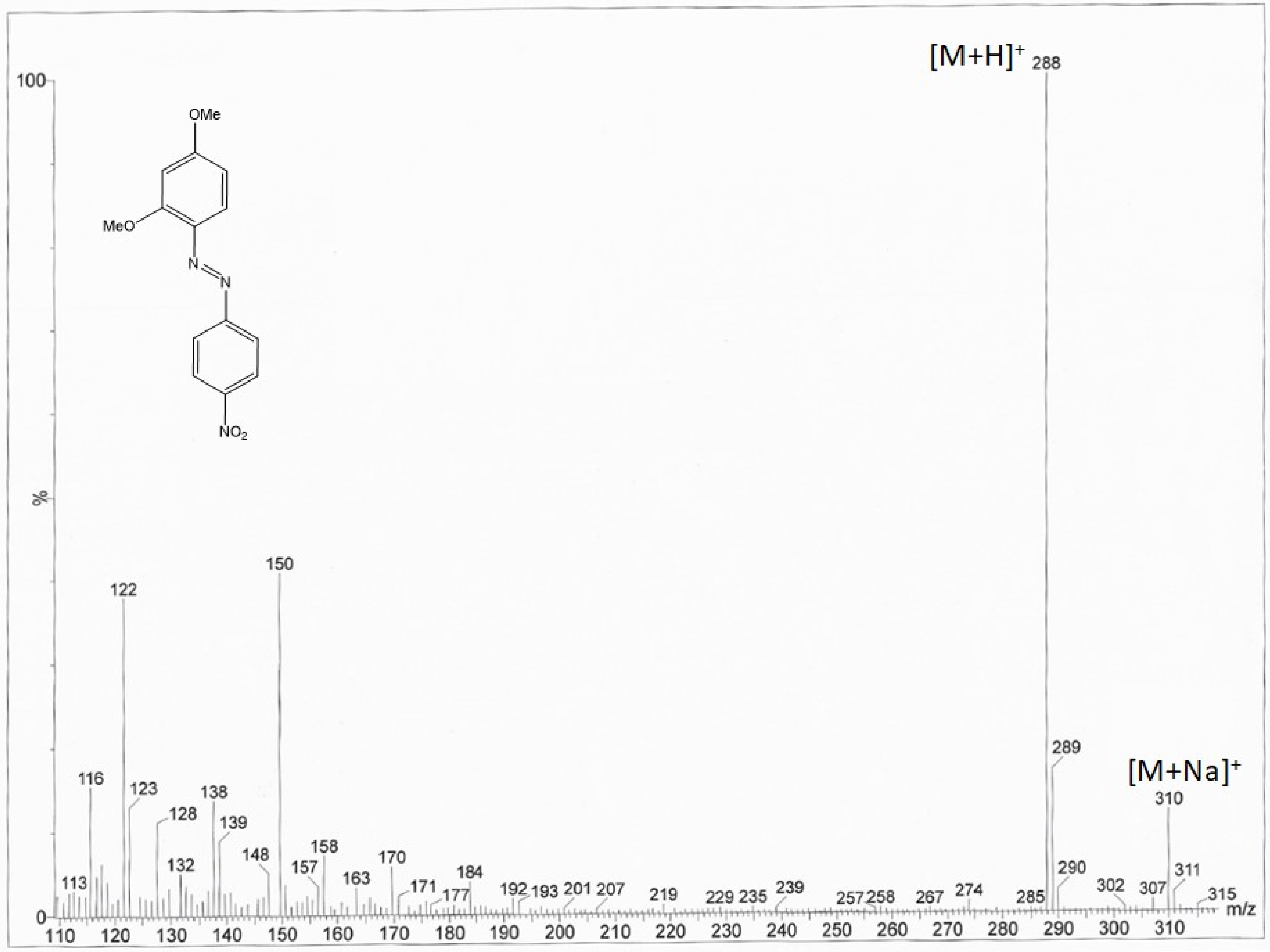

Fig. S51: ESI-MS$^+$ (*m/z*) spectrum of compound **9a**.

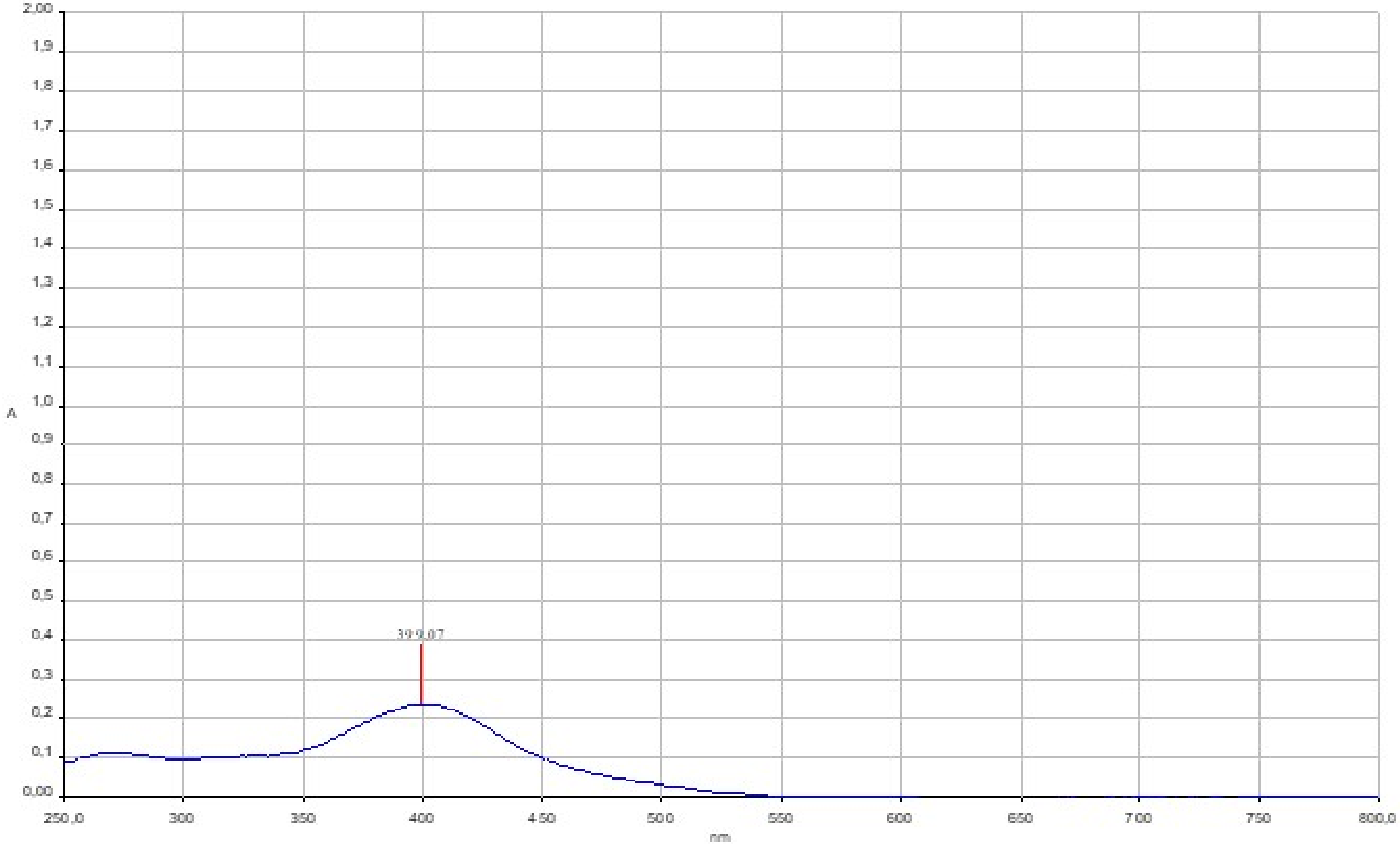

Fig. S52: UV-Vis spectrum of compound **9a** in $CHCl_3$.

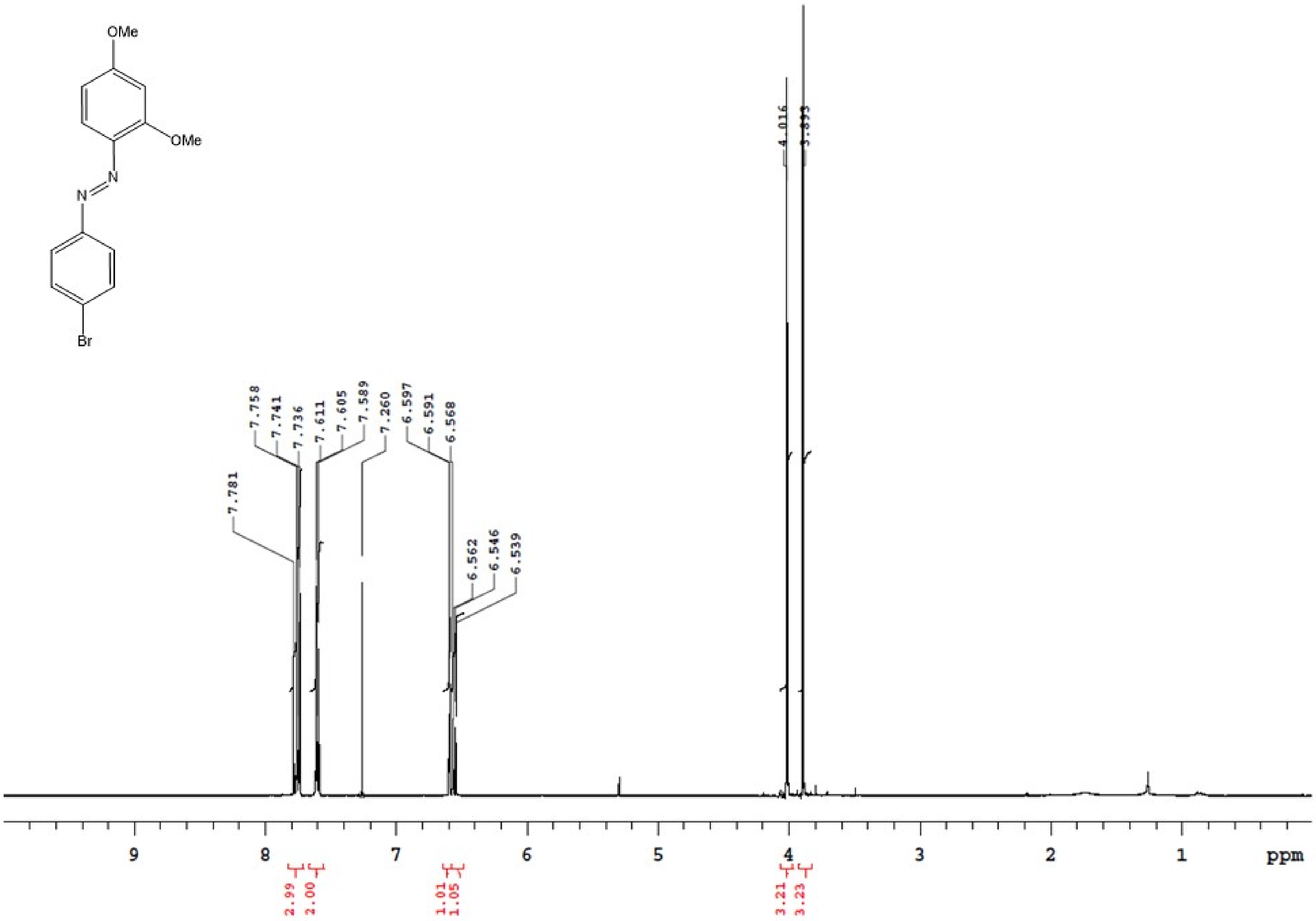

Fig. S53: ^{1}H-NMR spectrum of compound **9b** in $CDCl_3$.

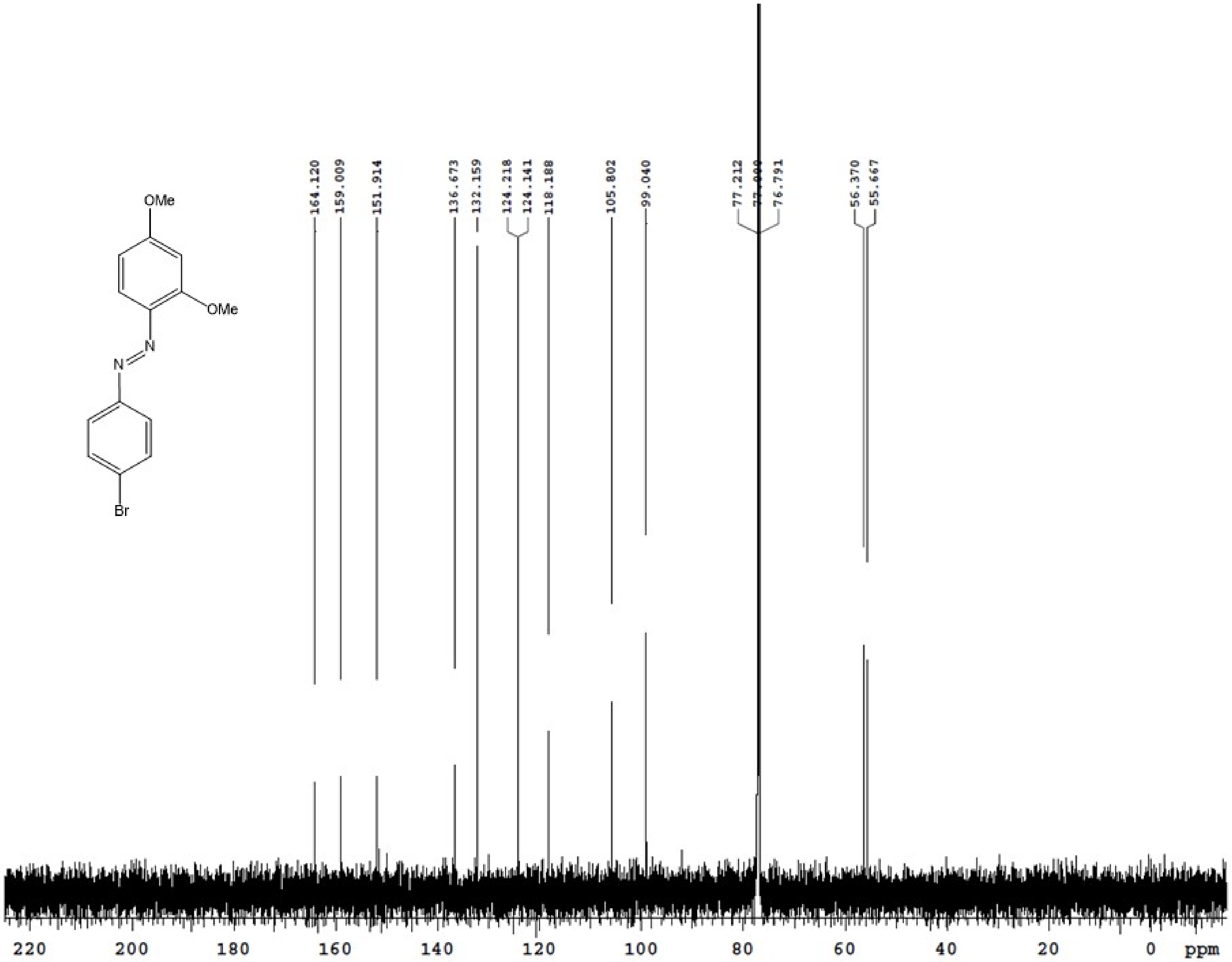

Fig. S54: ^{13}H-NMR spectrum of compound **9b** in $CDCl_3$.

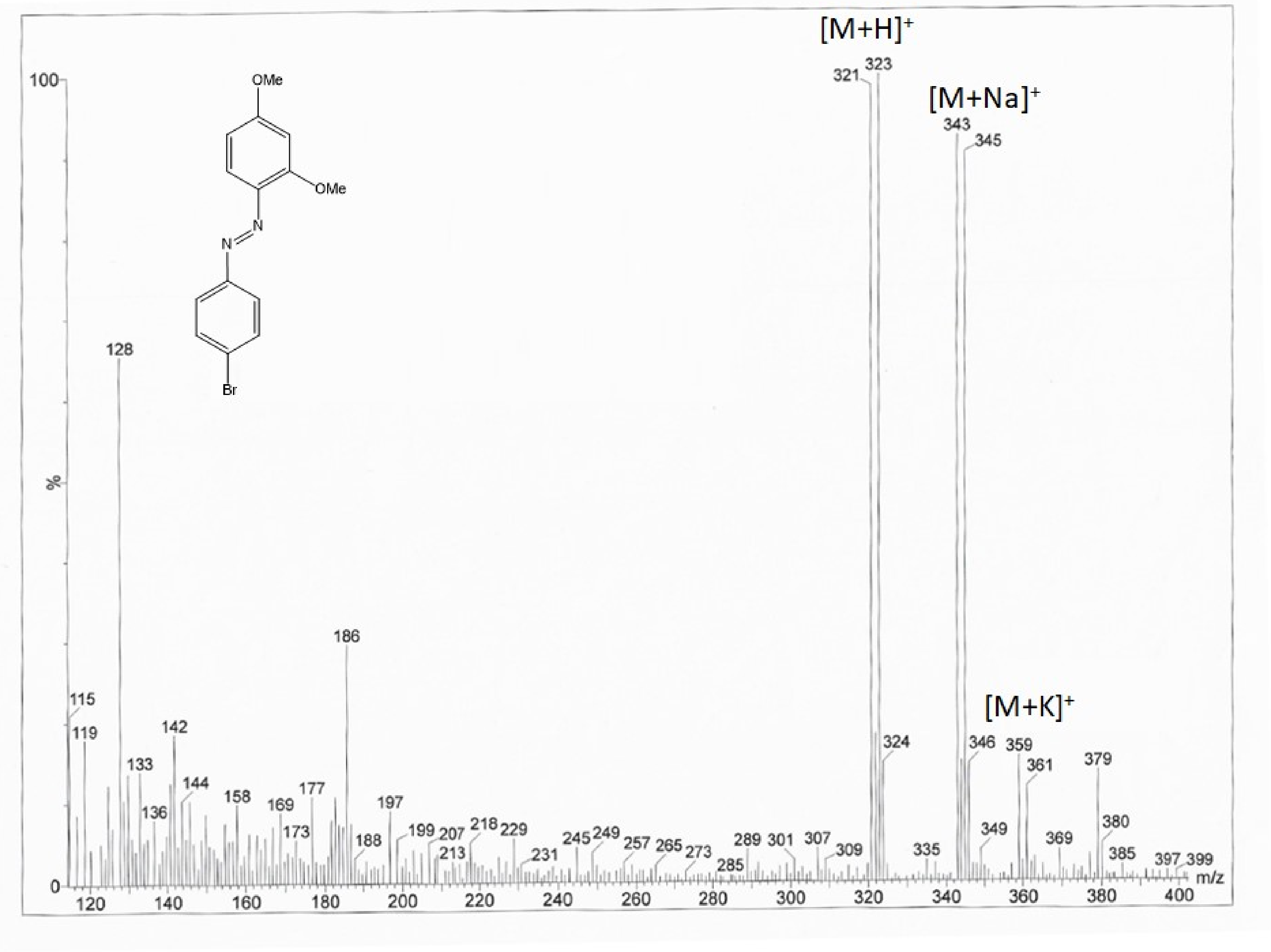

Fig. S55: ESI-MS+ (*m/*z) spectrum of compound **9b**.

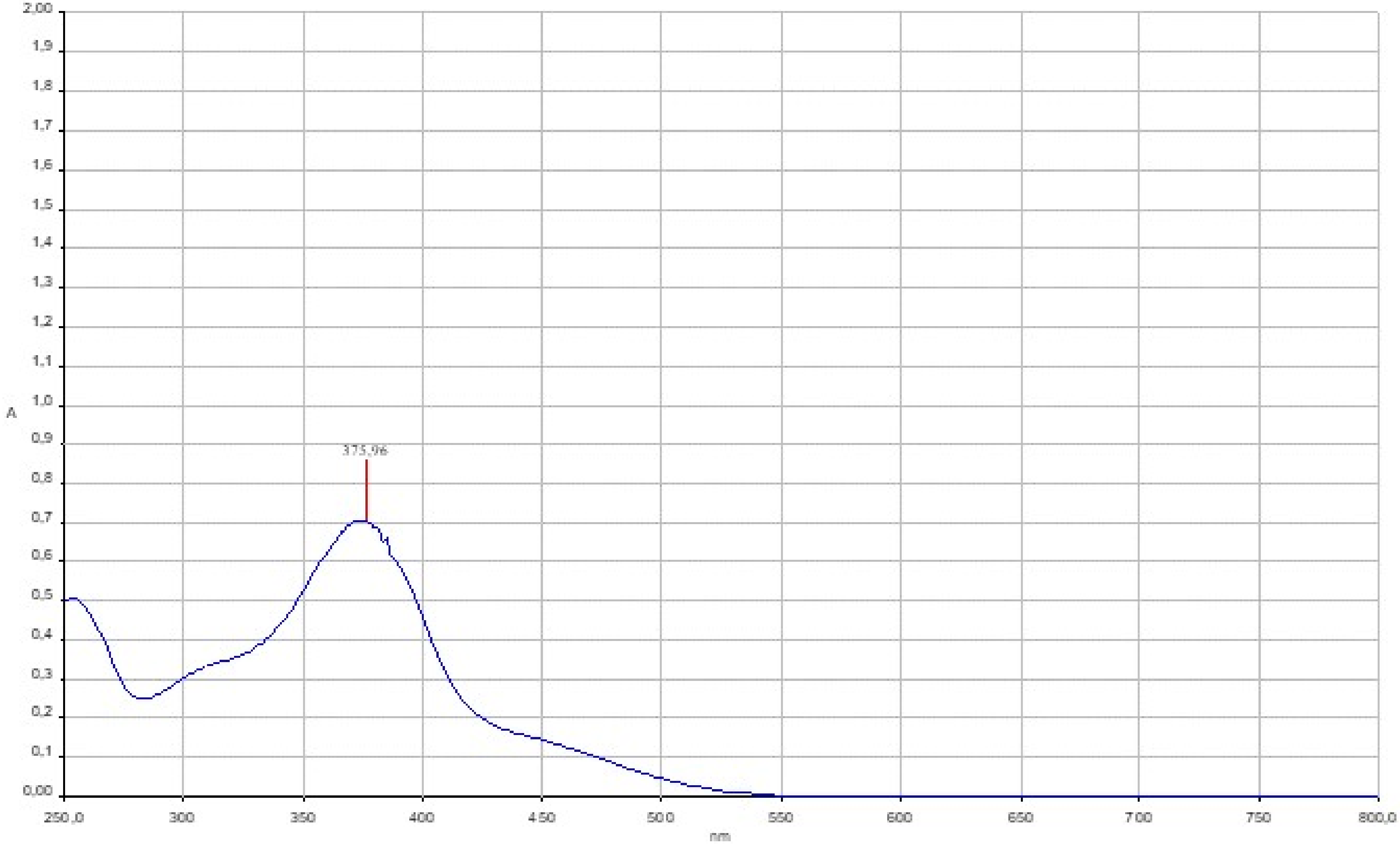

Fig. S56: UV-Vis spectrum of compound **9b** in $CHCl_3$.

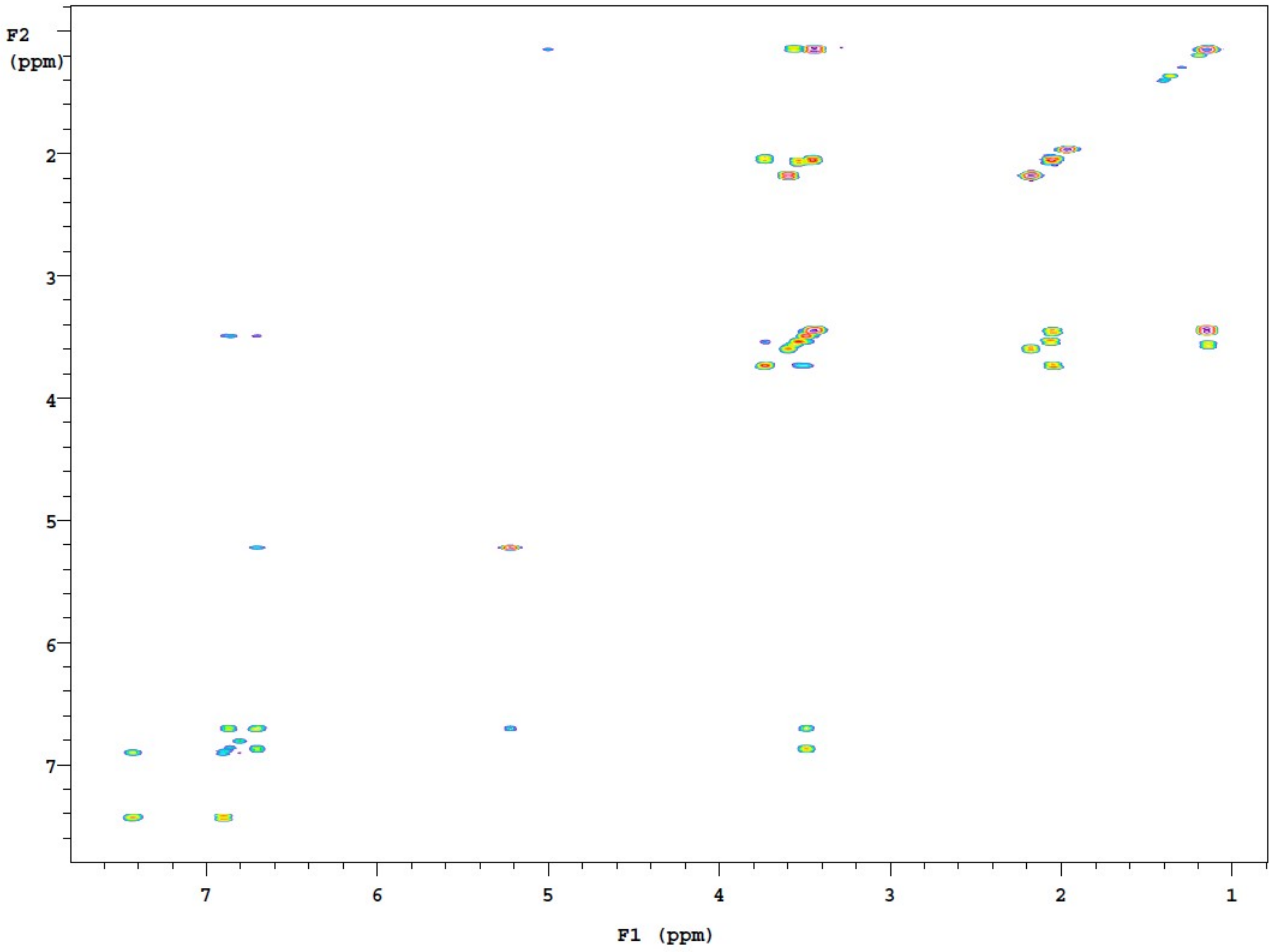

Fig. S57: COSY spectrum of the protonation reaction of compound **3**.

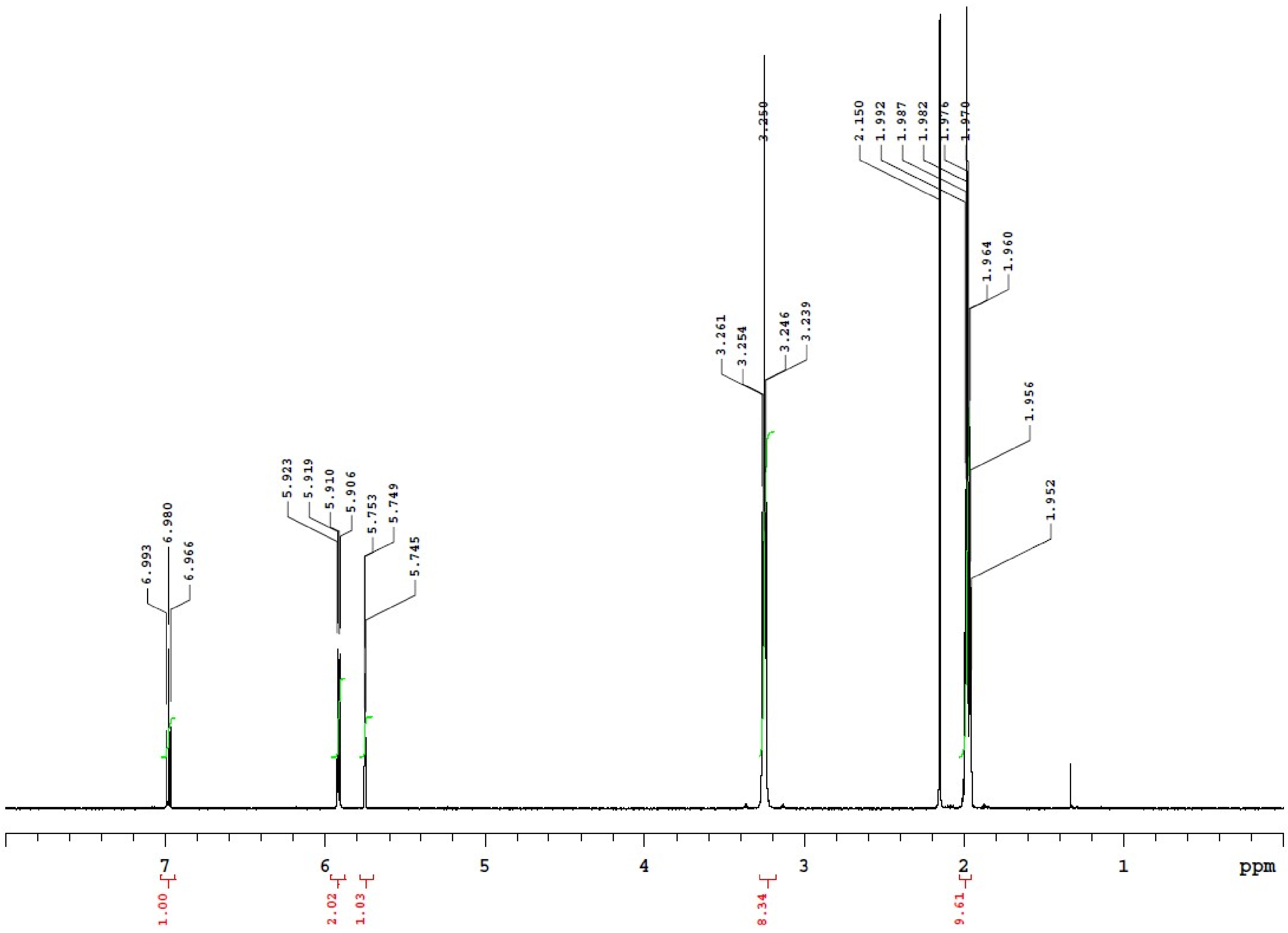

Fig. S58: ^{1}H-NMR spectrum of compound **3** in CD_3CN.
